# Genome-scale exploration of transcriptional regulation in the nisin Z producer *Lactococcus lactis* subsp. *lactis* IO-1

**DOI:** 10.1038/s41598-020-59731-8

**Published:** 2020-03-02

**Authors:** Naghmeh Poorinmohammad, Javad Hamedi, Ali Masoudi-Nejad

**Affiliations:** 1 Department of Microbial Biotechnology, School of Biology and Centre of Excellence in Phylogeny of Living Organisms, College of Science, University of Tehran, Tehran, Iran; 2 Microbial Technology and Products (MTP) Research Center, University of Tehran, Tehran, Iran; 3 Laboratory of Systems Biology and Bioinformatics (LBB), Institute of Biochemistry and Biophysics, University of Tehran, Tehran, Iran

**Keywords:** Computational models, Gene ontology

## Abstract

Transcription is of the most crucial steps of gene expression in bacteria, whose regulation guarantees the bacteria’s ability to adapt to varying environmental conditions. Discovering the molecular basis and genomic principles of the transcriptional regulation is thus one of the most important tasks in cellular and molecular biology. Here, a comprehensive phylogenetic footprinting framework was implemented to predict maximal regulons of *Lactococcus lactis* subsp. *lactis* IO-1, a lactic acid bacterium known for its high potentials in nisin Z production as well as efficient xylose consumption which have made it a promising biotechnological strain. A total set of 321 regulons covering more than 90% of all the bacterium’s operons have been elucidated and validated according to available data. Multiple novel biologically-relevant members were introduced amongst which *arsC*, *mtlA* and *mtl* operon for BusR, MtlR and XylR regulons can be named, respectively. Moreover, the effect of riboflavin on nisin biosynthesis was assessed *in vitro* and a negative correlation was observed. It is believed that understandings from such networks not only can be useful for studying transcriptional regulatory potentials of the target organism but also can be implemented in biotechnology to rationally design favorable production conditions.

## Introduction

Regulation at transcription level is one of the main mechanisms by which bacteria adapt their metabolism to changing environments. In response to these environmental or internal dynamics, controlling the gene expression is mainly exerted using transcription factors (TFs), proteins that specifically binds TF binding sites (TFBSs) or *cis*-regulatory motifs and in turn change the expression pattern of the downstream gene(s)^1,2^.

Bacterial genomes are organized in the form of *operons*, a group of genes in tandem sharing a terminator and a promoter each containing one or more TFBSs. The maximal number of co-regulated operons under the direct control of certain TF(s) is then called a regulon, a term which was firstly introduced by Maas *et al*. in 1964^3^. Studying the coordination of cellular behavior in response to internal or external signals in transcription level as a crucial aspect in microbial genomics depends on our ability to elucidate correctly all these regulons or by other means, the global transcriptional regulatory network (TRN) of a bacterium.

Although the most desired way of elucidating the entire set of regulons in a bacterium is experiment, serious challenges exist. Apart from being highly expensive and time-consuming^4^, laboratory-based elucidation of all regulons requires researchers to know exactly what conditions will trigger which regulons. Therefore, all conditions that will lead to activating all regulons must be known, a procedure which seems to be yet impossible^5^. Moreover, many bacterial TRN elucidation studies use gene expression profiles to predict the interactions of TFs and their target TFBSs^6–8^. However, there are two problems in this assumption: (a) not always a co-expressed situation denotes co-regulation^9^; and (b) post-transcriptional regulation of TFs can also occur and in this situation the cellular level of the TF is constant however the activity is altered. These issues can also compromise the whole predictions.

To this end, computational tools have been designed to elucidate all the co-regulated operons encoded in bacterial genomes with their main aim being to find the maximal sets of operons that share conserved *cis*-regulatory motifs (or in short, motifs)^10–12^. For this *ab initio* regulon inference, motifs must be scanned for all operons via phylogenetic footprinting technique. Since being introduced in 1988^13^, phylogenetic footprinting has greatly improved the computational motif identification process via using a comparative genomics analysis of closely related organisms^14^. Finding motifs of all operons in bacterial genomes then allows for further analysis to cluster conserved and similar motifs with the opinion that they will show similar regulation pattern, thus being classified as a regulon. Regulon prediction or TRN reconstruction is a key to understand gene function and evolution of the bacterium being studied.

*Lactococcus lactis* is one of the best-studied members of lactic acid bacteria (LAB) and can be classified into different subspecies. Of these, *L. lactis* subsp. *lactis* and *L. lactis* subsp. *cremoris* shows high biotechnological potentials ranging from their wide applications in production of fermented dairy products^15^ as well as flavor formation^16^ and lactic acid production (subsp. *lactis* and *cremoris*)^17^ to their implementation as drug delivery systems for many therapeutic proteins (subsp. *lactis*)^18,19^. Moreover, different strains of *L. lactis* subsp. *lactis* are known as bacteriocin producers. Lantibiotic nisin, the most intensively studied and used bacteriocin, is the first antimicrobial peptide approved by the FDA to be used as food preservative and is produced by many strains of *L. lactis* subsp. *lactis*^20^. According to other researches, new applications have been also proposed for nisin in biomedical field, such as its potential in treatment of colon intestinal infections^21^, bovine subclinical mastitis^22^ as well as its synergistic role in treatment of head and neck tumors and colorectal cancer^23,24^ and pan drug-resistant infections^25–27^. Due to the aforementioned potentials, lots of studies have been conducted to reveal the molecular basis of nisin production as well as other important features of this bacterium to both understand the biological basics and improvement of these potentials such as studies on nisin production or the bacterial cell growth improvement in different conditions ambient to various industries.

*L. lactis* subsp. *lactis* IO-1 is a highly potent strain in producing L-lactate from xylose as well as glucose being suggested as a promising candidate to be utilized in green plastic industry for the production of polylactic acid (PLA)^28^. The strain also produces a natural variant of nisin, nisin Z^29^, which differs from the typical nisin A peptide in only one amino acid which results in more solubility of nisin Z at physiological pH making it a more suitable candidate for biomedical applications.

Since the production of antibiotics is also proved to be regulated tightly at the transcriptional level^30^, in the present work we have considered reconstructing the global TRN of *L. lactis* subsp. *lactis* IO-1 by a successful phylogenetic footprinting-based approach which has provided an opportunity to better understand and study the regulatory behaviors of the organism by predicting all its regulons from the genomic data.

## Results and Discussion

### Computational TRN inference

#### Reference genome selection and operon identification

Among the phylum firmicutes, 74 bacterial genomes were finally selected as reference genomes set (Supplementary Table S1). Our analysis indicated that the addition of more genomes to this list will provide negligible benefit to the regulon predictions. Although a set containing as few as 25 genomes were shown to be able to cover near 89% of regulons inferred with the 74 genomes (Fig. S1), using a larger selection of organisms was considered as beneficial rather than the most appropriate organisms.

Accurate curation of predicted operons can greatly increase the regulon prediction. This is since the promoter upstream the first gene in an operon will be considered for motif discovery and mispredicted operons may result whether in false positive or false negative motifs. For instance, if *gene A* naturally forms a single membered operon within a bacterium’s genome and the prediction algorithm put it wrongly in the middle of a multi-membered operon, the motif of *gene A* will be missed and will not be taken into account in next steps of regulon prediction. The final set of 1373 operons of *L. lactis* subsp. *lactis* IO-1 are listed in Supplementary Table S2.

#### Motif finding

In a phylogenetic footprinting framework for motif discovery, finding sufficient orthologous operons for *all* operons in the target genome is another key step since an effective regulon inference relies directly on a sufficient and appropriate number of promoters containing corresponding motifs. Therefore, selecting the maximum number of non-redundant reference genomes (here 74) can benefit the regulon inference in this approach by giving more informative promoters for motif discovery.

Based on OrthoFinder results, the average number of orthologous operons for all operons of *L. lactis* subsp. *lactis* IO-1 is 34 with 81.3% of them having more than 6 orthologus operons. More than 95% of operons possessed over 2 orthologous operons, which were further explored to find conserved motifs. The total of over 5000 motifs was predicted while 89% of operons were predicted with at least 1 significant motif (*p*-value < 0.05). Therefore, genes without orthology or promoters showing no conservation at sequence level is underestimated which is regarded as an internal limitation of phylogenetic footprinting approach. However, here a relatively high percentage of operons were predicted with motifs. This is even higher than the percentage gained by the MP^3^ authors while evaluating the algorithm on *E. coli*^31^.

#### Regulon prediction and evaluation

**Figure 1:**
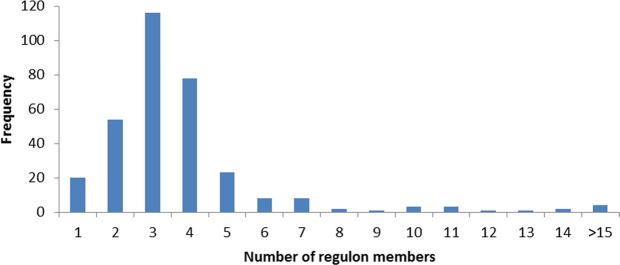
Frequency of predicted regulons with different size.

Co-regulation relationship between a pair of operons has been predicted via similarity comparison of their predicted motifs based on co-regulation score (CRS) (see methods) and as a result, a co-regulated graph and generated operon clusters for regulon prediction was built. Consequently, the reconstructed TRN of *L. lactis* subsp. *lactis* IO-1 includes 321 regulons covering 90.3% of all operons (Tables S3 and S4) with 232 of the regulons having more than 3 operons. Most of the predicted regulons include 2–4 operons (mostly 3) and 21 regulons were single-membered which does not comply with the definition for a regulon and thus a set of 300 final regulons were predicted (Fig. 1). However, although with lower scores, these operons (or single-membered regulons) are connected to other regulons’ members using their lower-score motifs (Table S5). To this end, they can be used for network analysis and thus cannot be permanently omitted from the final TRN.

Most of the regulons having more than 15 operons were predicted to be regulons regulated by global TFs such as CodY, CcpA, PurR. The analysis was performed by creating the positional weight matrices (PWM) of the consensus motifs for operons of these regulons and further finding the most statistically significant TF for each PWM by Tomtom motif comparison tool^32^. Table 1 shows the global TF regulated regulons and details on target operons can be found in Table S2. The presence of such regulons can be a positive point for evaluation of the present regulon prediction framework showing the success of the implemented procedure in clustering a large number of operons believed to be regulated by global TFs.

**Table 1:** Regulons with more than 15 members and their predicted TFs/TFBSs.

Regulon ID	Number of operons	Functions enriched	Predicted TF	Tomtom *P-value*
Regulon_309	30	Global catabolite repression	CcpA	1.02e-6
Regulon_91	15	Amino acid metabolism	CodY	5.54e-07
Regulon_323	16	Purine metabolism	PurR	1.37e-04

The presence of other orthologous lactococcal TF regulons was also studied to further evaluate the regulon prediction. Such data were mostly available for *L. lactis* subsp. *lactis* IL1403 from literature and RegPrecise database and to a lesser extent some literature data were used for *L. lactis* subsp. *lactis* KF147^33^ and *L. lactis* subsp. *lactis* F44^34^. Focusing on the known TF regulons in these organisms, motifs of orthologous operons in *L. lactis* subsp. *lactis* IO-1 were used to verify that they can be governed by the same TF as in the other *L. lactis* strains under study, using Tomtom (*p-value* < 0.05).

As a result, from 31 known lactococcal regulons studied, 24 regulons were assigned with TFs similar to those regulating the orthologous regulon. In most cases, the orthologus regulon in *L. lactis* subsp. *lactis* IO-1 were significantly similar to the known regulons meaning that in some cases there were some differences in terms of the number of constituting operons. Accordingly, the IO-1 orthologous regulons often possessed a similar or higher number of operons which can both be regarded to as either a false positive or a new member of a previously-unknown regulon, the verification of which needs whether experimental verification or having sufficient biological basis.

Finding novel potential target operons/genes or generally, novel regulons using computational regulon prediction is an accepted routine. For instance, using a knowledge-driven comparative-genomics approach, Leyn *et al*. have found about 140 new target genes for 93 previously studied TF regulons in *B. subtilis* genome which provides useful data with potentials to be readily used in genetic experiments, metabolic modeling, and evolutionary analysis^35^. Here, totally 30 new regulon members have been predicted for BirA, BusR, CmbR, CodY, CopR, FacT, hrcA, MtlR, NrdR, Rex, and XylR orthologous TF regulons in *L. lactis* subsp. *lactis* TRN (Table 2).

**Table 2:** Orthologous TF regulons in *L. lactis* subsp. *lactis* IO-1 compared to known lactococcal regulons.

Reference strain^*^	TF assigned	RegulonID	Biological process	Ratio of the number operons^**^ (in IO-1/ in reference strain)	Novel predicted target genes
IL1403	AdcR	Regulon_212	Zinc homeostasis	2/2	—
	ArgR	Regulon_308	Arginine biosynthesis; Arginine degradation	5/6	—
	BirA	Regulon_155	Biotin biosynthesis	2/2	*yseC, yseD,fabG2,yseE*
	BusR	Regulon_52	Betaine utilization	4/1	*arsC, hom, thrB*
	CcpA	Regulon_309	Global catabolite repression	30/31	—
	CmbR	Regulon_310		10/9	*ymhA*
	CodY	Regulon_91	Amino acid metabolism	15/12	*optA, dppB, lilo_0313, optD, optF, yuaE, sdaB, gltA, citB, icd*
	CopR	Regulon_312	Copper homeostasis	5/5	*yuhE, yihD*
	FabT	Regulon_313	Fatty acid biosynthesis	4/3	*ymdE*
	GlnR	Regulon_314	Nitrogen assimilation	3/3	—
	HomR	Regulon_315	Methionine metabolism; Cysteine metabolism	2/2	—
	HrcA	Regulon_316	Heat shock response	4/3	*yudG, yudH*
	MleR	Regulon_297	Malate utilization	1/1	—
	MntR	Regulon_318	Manganese homeostasis	2/2	—
	MtlR	Regulon_319	Mannitol utilization	1/1	*mtlA*
	NagR	Regulon_320	N-acetylglucosamine utilization	4/3	
	NrdR	Regulon_321	Deoxyribonucleotide biosynthesis	2/2	*rpsP*
	PerR	Regulon_137	Iron homeostasis; Manganese homeostasis; Oxidative stress response	1/1	—
	PurR	Regulon_323	Purine metabolism	14/16	—
	Rex	Regulon_324	Energy metabolism	7/7	*ylaG, nadE*
F44	YthA	Regulon_325	(Acid) stress response	10/10	—
KF147	XylR	Regulon_10	Xylose metabolism	4/3	*mtlA, mtlR, mtlF, mtlD*,

*IL1403: *L. lactis* subsp. *lactis* IL1403; F44: *L. lactis* subsp. *lactis* F44*, L. lactis* subsp. *lactis* KF147.

**The operons in reference strains’ regulons are assumed to include same set of genes as in IO-1 except when underlined. If there are novel genes in the regulons with same number of operons due to difference in operon structure, they are shown in the leftmost column.

BusR is a repressor of the expression of osmotically inducible glycine‐betaine uptake system at low osmolarity in *L. lactis*, thus having a key role in osmoregulation^36^. Available studies on BusR regulon of lactococci introduce it as a small regulon consisting of *busA* and *busB* genes which encodes transporter for glycine betaine^36^ which is one of the most important of compatible solutes and osmostress protectants in *L. lactis*^37^.

Among novel members of the BusR regulon, we found *arsC* which codes for arsenic reductase and is potentially involved in arsenic detoxification^38^. It is reported that in a different organism such as plants and microorganisms, changes in osmolytes such as glycine betaine content occurs accompanying the exposure of the organism to arsenic toxicity^39,40^. On the other hand, the biosynthesis of arsenobetaine which is homologue glycine betaine and known as an osmoprotectant, also starts with the *arsC* activity in microorganisms but the exact enzymatic and genetic data on its biosynthesis is not clear^41^.

On the other hand, Cheung *et al*. have studied the expression pattern of genes in *E.coli* under osmotic stress amongst which *hom(thrA)* and *thrB* expressions showed 6-fold change denoting their apparent role in osmotic stress^42^. Since there are basic overlaps in osmotic stress responses among bacteria, further study of the role of mentioned genes in osmotic stress response in *L. lactis* can be advantageous in better understanding its physiology. Moreover, *hom(thrA)* and *thrB* participate in the biosynthesis of amino acids such as threonine, they can also directly affect osmopotectants production. This is due to some evidence that threonine metabolites have been also shown to play roles in defenses against salt stress^43^. Accordingly, the presence of both *arsC* and the operon of *thrAB* can be meaningful in the BusR regulon.

For CodY regulon which includes mainly the amino acid metabolism components, genes encoding oligopeptide transporter (*optA, optD, optF, dppB, lilo_0313*), L-serine dehydratase (*sdaB*), putative aspartate protease (*yuaE*) and enzymes of citrate cycle (*gltA, citB, icd)* all of which are apparently participated in amino acid metabolism were predicted as new targets. *gltA, citB and icd* are also reported to be a member of CodY regulon of *Bacillus subtilis*^44^.

Among other new predicted targets, *yuhE* (encoding a copper transpoter), *yudG* (encoding a heat shock peotein), *mtlA* (encoding a mannitol-specific PTS system component) and *nadE* (encoding NAD-synthetase) in CopR, HrcA, MtlR and NrdR regulons, respectively, are genes whose potential annotated functions are related to their corresponding regulon’s biological process.

Generally, determination of TFs and their specificity to a target (TFBS or motif) is highly complicated by the layers of complexity due to the existence of many features modulating this relationship beyond sequence motifs^45^. Of the most important complexities are: relative incapability of experimental techniques to characterize the specificities of all TFs due to the need of expressing soluble and active TFs of interest, possibility of multiple specificities for an individual transcription factor^46^, possibility of cooperative binding by multiple TFs as well as TFs-small molecules or TFs-proteins which in turn can also give rise to new motifs^45^, and finally the effects of contextual and epigenetic information of DNA that may affect TF binding^47^. Moreover, the direct transposition of a model organism’s (e.g. using *Bacillus subtilis* in this case) regulatory information does not always allow building a reliable regulatory network in the target genome since many regulators have functions that could not be proposed by transposition of the knowledge currently available in other bacteria^48^.

Consequently, computational assignment of a known TF to predicted regulons usually is not straightforward and more data is needed to evaluate and analysis the regulons. However, elucidating all regulons of a genome can give useful insight into potential cooperating operons/genes. Noteworthy in this respect is that not all the predicted regulons should be regarded as being available at the same time in a bacterial cell, rather a regulon or a subset of the whole predicted TRN is triggered in a specific environmental of physiological condition. Such data on potential transcriptional responses in different bacteria can also be used in comparative omics analyses to differentiate close taxa.

#### Findings on xylose metabolism regulation at the transcriptional level

Very few strains of *L. lactis* from non-dairy sources (plant-based habitats) can ferment xylose; and to-date the most potent one is *L. lactis* subsp. *lactis* IO-1^49^. In biotechnological productions, using cheap raw material as carbon sources greatly increase the economic feasibility of the production. Xylose can be derived from the hemicellulose abundant in agricultural wastes and could be an alternative carbon source for potent biotechnological strains. *L. lactis* subsp. *lactis* IO-1, well-known for its potential in producing nisin Z and lactic acid is reported to perform better than the type strain *L. lactis* in its industrial application in *L-lactate* fermentation^50^, thus the xylose fermentation in this bacteria have been studied mostly via fermentation analysis^51^. The data from the present study can be complementary with experimental results which can help better understand the xylose metabolism in this organism.

According to Tables 2 and S3, apart from *xylA, xylB and xylR* as the main genes in xylose metabolism, the predicted XylR regulon in *L. lactis* subsp. *lactis* IO-1, is shown to possess genes involved in mannitol transport and metabolism which shows a potential overlapping genetic responses to xylose and mannitol. Accordingly, in the plant-associated *Pseudomonas fluorescens* SBW25 which is also able to grow on xylose as a sole carbon source, expression of the xylulokinase was shown to be mediated by the mannitol-responsive regulator *MtlR* by xylulose being the inducer and therefore both MtlR and xylR were shown to regulate xylose utilization^52^. The mechanism seems to be reasonable since as shown in the present study, there is the possibility of such novel mechanism in which two AraC-type TFs (XylR and MtlR) being involved in xylose utilization in *L. lactis* subsp. *lactis* IO-1 in terms of the mode of regulation. The fact that xylose and mannitol are mostly co-present in the plant environment can also help the idea as these will help the colonization of plant-associated bacteria^52^.

Although catabolite repression by glucose is a known feature for all xylose metabolizers, the genes of *xyl* operon was not clustered in CcpA regulon, even when tested the regulon prediction with lower motif similarity cut off. This is in line with a study by Erlandson *et al*. in which they could not find a *cis*-acting sites upstream of *xylA* or *xylB* and suggested that the strain might have a homologue of it^49^. To further verify this, we have implemented AME, a motif enrichment tool from MEME suit^53^ and analyzed the promoter of xylose operon to find any homologue CcpA motif with motif of *xyl* operon from *Lactobacillus pentosus*^54^, *Staphylococcus xylosus*^55^ and *Bacillus subtilis*^56^. Our results also show no homologue CcpA-specific motif upstream of these genes.

#### Findings on nisin biosynthesis regulation at the transcriptional level

**Figure 2:**
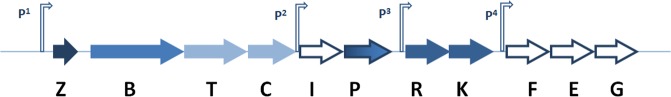
Schematic presentation of nisin Z biosynthesis gene cluster with four characterized operons: nisZBTC, nisIP, nisRK and nisFEG. (Promoters numbers are shown as p1 to p4, these also represents operons numbers).

Another important feature of *L. lactis subsp. lactis IO-1* is the production of nisin Z. Due to its widespread use mainly as an antimicrobial agent in the food industry, the research on its biology and biotechnology is of considerable interest. Unlike a large amount of studies on the effect of culture medium formulations, culture conditions and basic genetics study on nisin biosynthesis gene cluster, few reports exist on the molecular basis of nisin biosynthesis in relation with other components of the genome and its transcriptional regulation. Generally, *nisZBTCIPRKFEG* cluster encoding nisin Z peptide, modifying enzymes, transport, regulatory and immunity-related proteins which are organized in 4 operons (Fig. 2), is known to be regulated by a two-component system *nisRK* which triggers P^1^ and P^4^ promoters by autoinduction.

There is no information on the regulatory potential of P^2^ and P^3^ promoters and while the expression of NisZBTC and NisFEG are thought to be regulated by NisRK and since no specific regulator is encoded by the *nis* operon, other operons are believed to be under the control of a general regulator^57^. The role of alternative σ factors has been studied for subtilin, a lantibiotic produced by *B. subtilis* which is very closely related to nisin with respect to structure, activity and genetic traits. Subtilin biosynthesis was reported to be controlled by an alternative σ factor (σH)-mediated transcriptional regulation other than the known autoinduction regulatory mechanism^30^. Accordingly, nisin promoters were studied to see if the mechanism is potentially available. However, no orthologs for σH was found in *L. lactis* subsp *lactis* IO-1. Furthermore, no hits were found when Bioprospector^58^ was used to search for any enriched motif known of the two sigma factors of *L. lactis* subsp. *lactis* IO-1, RpoD (or σ39)^59^ and ComX^60^ within P^2^ and P^3^ sequences. Although the presence of (alternative) sigma factors is generally proposed for *L. lactis* (Araya-Kojima, Ishibashi *et al*. 1995), further experimental analysis is required to verify the presence or absence of such regulation on nisin operons.

Generally, from the regulon prediction results, 6 regulons (filtered with motif similarity cut off of more than 0.5) have been illustrated within which one or more nisin operons were present (Table 3). According to Table 3, nisin operons are shown to have potential co-regulatory relationships with operons responsible for cell wall, aminoacid, vitamin, and DNA metabolism. Except for a few operons without yet-meaningful explanation of their relationship to nisin operon, the predicted operons with potential co-regulation relations with nisin have been directly or indirectly introduced by other studies to be related to nisin biosynthesis.

**Table 3:** Predicted nisin regulons and their properties.

RegulonID	Members (operons)	Biological Function	Relevance to nisin^*^ (reference)
Regulon_303	Operon_362	Nisin biosynthesis	—
	Operon_1254	Arginine metabolism	Less acidic environment as a result of ammonium production from arginine metabolism increases nisin immunity^62^
	Operon_1378	Arginine metabolism	Less acidic environment as a result of ammonium production from arginine metabolism increases nisin immunity^62^
Regulon_304	Operon_362	Nisin biosynthesis	—
	Operon_364	Nisin biosynthesis	—
	Operon_620	Unclassified (hypothetical protein)	NYK
	Operon_586	Riboflavin metabolism	NYK
Regulon_305	Operon_364	Nisin biosynthesis	—
	Operon_362	Nisin biosynthesis	—
	Operon_745	Lipoteichoic acid (LTA) biosynthesis	Cell wall biosynthesis has positive correlation with resistance to cationic antimicrobial peptides^63^
	Operon_608	Pyrimidine biosynthesis	Improves cell growth by being involved in DNA recombination and repair. Nisin production is growth-dependent.
Regulon_306	Operon_362	Nisin biosynthesis	—
	Operon_366	Nisin biosynthesis	—
	Operon_1254	Arginine metabolism	Less acidic environment as a result of ammonium production from arginine metabolism increases nisin immunity^62^
	Operon_32	Unclassified	NYK
Regulon_307	Operon_362	Nisin biosynthesis	—
	Operon_29	Cysteine and methionine metabolism	Many amino acids (like cysteine) highly stimulate nisin production by playing a precursor role^89^
	Operon_1292	Glycine, serine and threonine metabolism	Many amino acids (like serine and threonine) highly stimulate nisin production by playing a precursor role^89^
	Operon_1201	Unclassified (hypothetical protein)	NYK
Regulon_326	Operon_362	Nisin biosynthesis	—
	Operon_586	Riboflavin biosynthesis	NYK

*NYK: Not Yet Known.

It has been long known that the level of nisin produced could be affected by the level of the producer’s immunity to nisin and there have been studies showing the improvement of nisin production by the overexpression of nisin immunity genes (*nisI*, *nisFEG*) in the producer strain^61^. Using transcriptome analysis, Kramer *et al*. reported different potential mechanisms, other than the direct effect of the nisin immunity genes, by which *L. lactis* acquires nisin resistance^62^. Many of their discovered mechanisms such as the role of arginine metabolism genes (*arc* operon) and LTA biosynthesis genes (*dlt* operon) have been also predicted to be correlated with nisin in our study. Accordingly, genes of *dlt* operon encodes enzymes for LTA biosynthesis, thus they are a part of cell wall biogenesis which is a deterministic factor in resistance to cationic antibacterial peptides^63,64^ and can provide the producer strain better immunity and in turn a higher threshold for production.

*arc* genes, on the other hand, are involved in the breakdown of arginine via the arginine deiminase pathway^65^ and their involvement in nisin resistance/biosynthesis could be due to the activity of enzymes in completely degrading arginine to ammonium which will result in a locally less acidic pH at the outer side of the cytoplasmic membrane and hinder its binding to its anchor on membrane^62^.

As another regulon prediction result, nisin and riboflavin operons has been shown to cluster in a regulon in multiple cases. Bacteria supply their versatile demands for riboflavin by encoding both biosynthetic enzymes and importer proteins. *L. lactis* subsp. *lactis* IO-1 possess both riboflavin biosynthesis and transport genes encoding in operons such as *ribGBAH* as well as other monocystronic genes like *ribC*, *ynaE and* ypaA. *ribGBAH* and *ribC* encodes biosynthetic enzyme to produce riboflavin, FMN and FAD from GTP and ribulose-5-phosphate while *ynaE* and *ypaA* code for transporter similar to riboflavin transporters known in *B. subtilis*^66^. Generally, it is proposed that in situations when riboflavin is sufficiently available in the environment, bacteria prefer to uptake rather than biosynthesis the vitamin and therefore they shut down the biosynthetic genes, thus avoiding waste of metabolic energy^67^. Here, regulon prediction results shows that *ribGBAH*, but not other *rib* genes have been in many cases clustered with *nis* operons in a regulon. Thus the TRN analysis suggests that riboflavin biosynthesis and nisin production may have regulatory correlation. There has been no report before on the probability of their correlation.

The most well-studied regulation mechanism on some of riboflavin biosynthesis genes is the FMN riboswitch whose alternative secondary structures can repress or induce the expression of downstream genes by sensing the flavins. Studies in different bacteria have later shown that the regulation is affected by role of different proteins^68–70^. These findings open the possibility for divergent regulatory mechanisms and suggest that there is still a long way to fully characterize riboflavin biosynthesis regulation mechanisms.

Moreover, there have been proteins such as RibR in *B. subtilis* found whose C-terminus shows regulatory roles on the FMN riboswitch, but no orthologs have been shown in any other bacteria (and also in IO-1). Therefore, search for similar regulatory proteins in other bacteria can be an interesting line of study. Although no statistically significant TF could be assigned to the similar motifs based on which *rib* and *nis* operons were clustered in a regulon, such regulatory proteins may elucidate the mechanism of this correlation.

On the other hand. there are a few reports about the role of transcription factors in regulating LAB’s lantibiotics^71,72^, one of which (LasX in *Lactobacillus sakei*) shows a bifunctional regulatory pattern by being both activator and repressor for different genes. Since there are many uncharacterized genes which are annotated as putative transcriptional regulators in the genome of IO-1 and other nisin-producing lactoccoci, similar mechanisms could also potentially describe the correlations such as what we have shown for *rib* and *nis* genes.

More relevant explanations on other co-clustered operons with nisin are summarized in Table 3.

### Effect of riboflavin on nisin Z production by *L. lactis* IO-1

Fermentation-based experiments were implemented to study the effect of riboflavin on nisin Z biosynthesis.To this end, the effect of varying concentrations of riboflavin in the culture medium as well as riboflavin over producer *L. lactis* subsp. *lactis* IO-1 strains were studied on nisin production.

**Figure 3:**
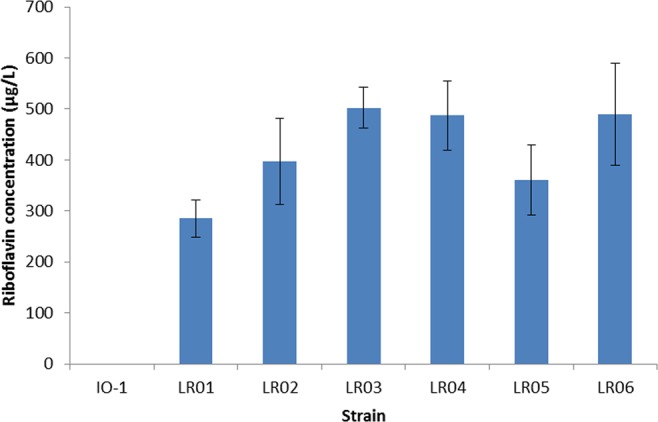
Riboflavin concentration produced by of roseoflavin-resistant strains (*L. lactis* subsp. *lactis* IO-1 is shown as control).

**Figure 4:**
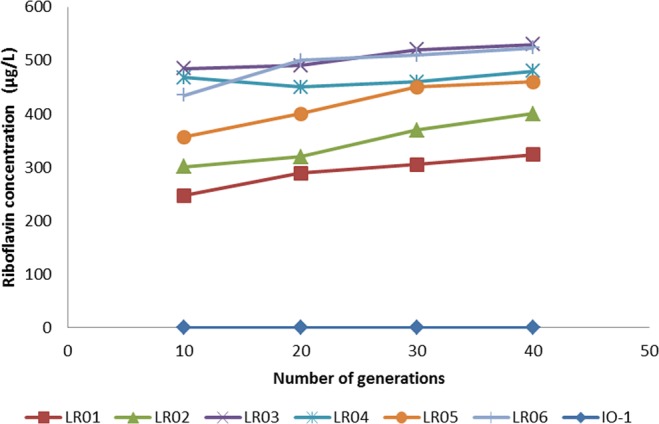
Stability of riboflavin production of the mutants after 40 generations (*L. lactis* subsp. *lactis* IO-1 is shown as control and concentration values are the average of three independent experiments).

**Figure 5:**
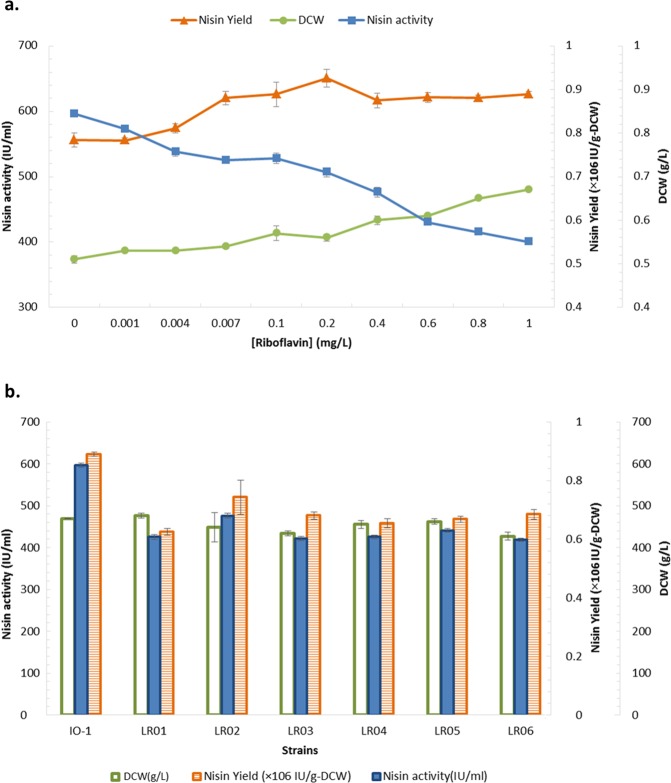
Effect of riboflavin on nisin Z production of *L. lactis* IO-1, studied in two conditions: (**a**) Different riboflavin concentrations in culture medium of *L. lactis* IO-1 and (**b**) assay using riboflavin overproducer mutant strains. (DCW: Dry cell wight).

HPLC assay of the cell-free supernatant of 6 roseoflavin-resistant mutants in riboflavin-free CDM after 8 hours showed the isolates are riboflavin overproducers (Fig. 3), ranging from about 280 to 500 µg/L riboflavin. All 6 strains were shown to be stable in overproducing riboflavin (Fig. 4). These strains were used to analyze their nisin production. The fermentation experiments were performed in two conditions: a. Different riboflavin concentration (from 0–1 mg/L) in culture media and effect on nisin production; b. Overproducing riboflavin mutant strains and effects on their nisin production (Fig. 5 and Table S6).

As can be seen in Fig. 5, in the media with decreasing concentrations of riboflavin, no significant difference can be observed until the near-zero riboflavin concentrations (0.1 mg/L). Since in sufficient concentrations of riboflavin, uptake is preferred by bacteria, it is believed that the biosynthetic genes have low or no activity until near zero riboflavin concentrations where these enzymes will be derepressed^67^. In this situation, there is a statistically significant decrease in nisin yield (*p*-value < 0.05) which can show the role of riboflavin biosynthesis on nisin production. The correlation seems to be negative as nisin biosynthesis decreased with increased riboflavin biosynthetic potential. To further study this negative correlation, stable riboflavin overproducers were isolated from roseflavin resistant mutants described in the methods and materials section.

All six riboflavin overproducers were shown to have lower nisin Z yield in comparison to the wild *L. lactis* subsp. *lactis* IO-1 under the same culture conditions. It is found that almost in all of the studied LABs, roseoflavin mutants with the riboflavin overproduction phenotype contain mutations in the regulatory region upstream of the *ribGBAH* operon and it is proposed that these mutations will disturb terminator structure stability which in turn allows for continued transcription of the rib operon^73^. Moreover, there have been also mutations found in *ribC* gene which play roles in converting riboflavin to FMN. Having continuously-active riboflavin biosynthetic genes, again nisin biosynthesis was confirmed with a negative correlation as seen with condition “a” (Fig. 5).

## Conclusions

In the present work, a phylogenetic footprinting-based approach was implemented to fully elucidate all potential regulons in a nisin Z producing *Lactococcus lactis* with the aim of providing a systems-level insight able to aid in understanding the underlying regulatory mechanisms in this bacterium. According to a comparison with the existing knowledge on the transcription regulation in its close strains, the TRN was validated to be accurate and reasonable. For many of the predicted regulons, TFs could be assigned and multiple new targets were also introduced with meaningful correlations to their corresponding regulons. Even for regulons without assigned TFs such as nisin and riboflavin-including regulons, experimental tests showed the accuracy of the prediction.

As of important findings of inherent properties of *L. lactis* subsp. *lactis* IO-1 such as its nisin Z production potential, we have found for the first time the correlation of riboflavin and nisin biosynthesis which was shown to be negative in fermentation-based studies. The predicted regulatory link proposed by the TRN, proposed a more complex regulation on nisin or riboflavin biosynthesis. Accordingly, further evaluatioations can fully elucidate the exact mechanism behind this relationship which is of interest to both microbiologists and microbial biotechnologists.

In conclusion, the collection of regulons for the *L. lactis* subsp*. lactis IO-1* obtained in this work not only can be used to better understand the unexplored physiology of this potent bacterium and rational implementation of the findings in biotechnology, but can be implemented as a template to reconstruct the TRN of other *L. lactis* strains with modifications based on comparative genomics.

## Methods

### TRN reconstruction

**Figure 6:**
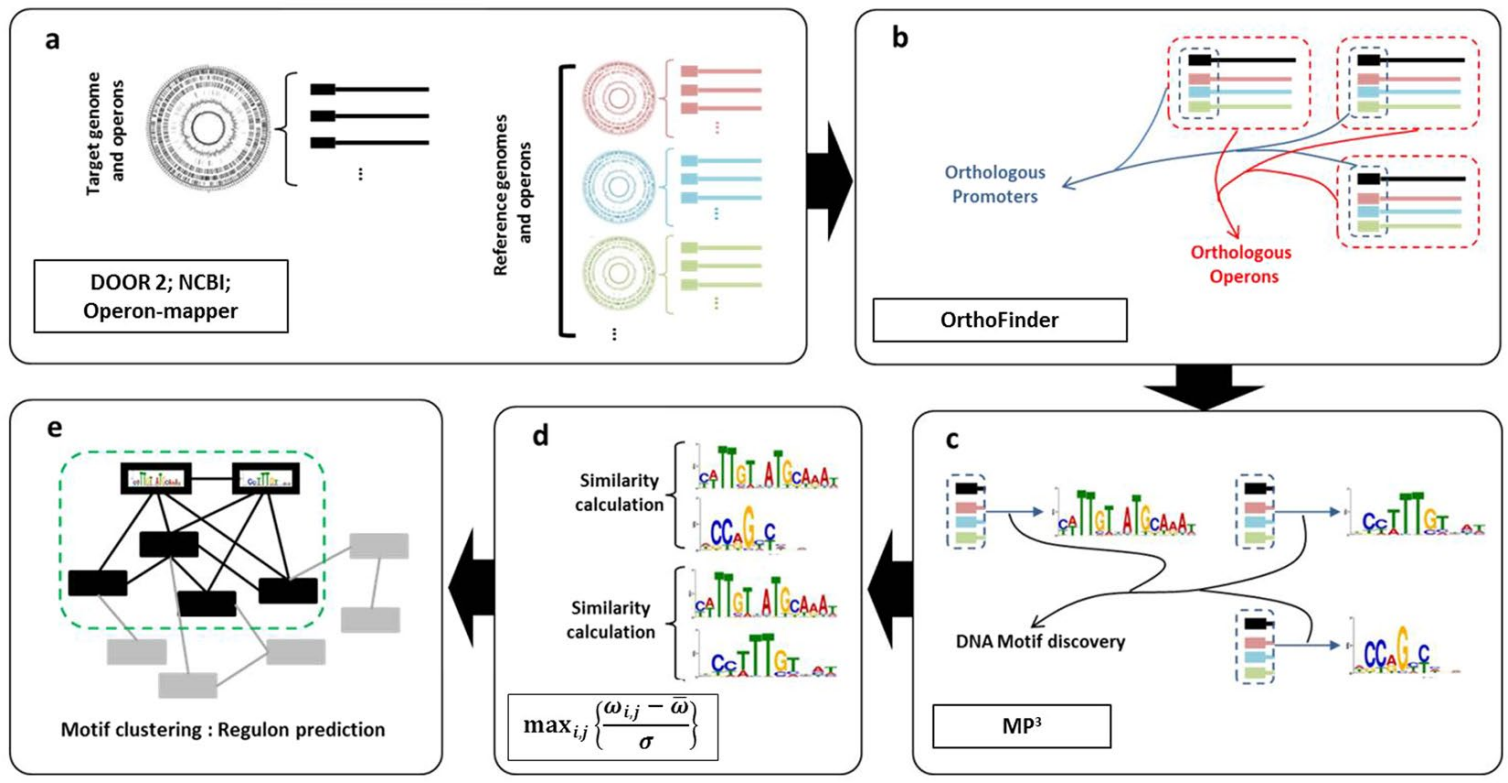
The comparative genomics-based regulon prediction framework used. (**a**) Reference genome selection and data retrieval, operons of the target and reference genome can be retrieved or predicted by proper tools. (**b**) Orthology identification for genes in the target genome. Relatively all of the genes in the target genome should have orthology among reference genomes to make phylogenetic footprinting feasible. Operons from target and reference genome sharing at least one orthologous gene is designated as an orthologous operon. The promoter sequence of these operons are used for motif finding (**c**). Phylogenetic foot printing’s final step is to find DNA cis-regulatory motifs (**d**). Similarity score calculation (co-regulation score: see text) based on which interactions between motifs of operons were made (**e**). Regulons prediction by clustering the resulting network, each cluster is a regulon.

The reconstruction of a genome-based global TRN for *L. lactis* subsp. *lactis* IO-1 was performed using a four-step comparative genomics-based approach which is described stepwise below and is depicted in Fig. 6.

#### Target and reference genome set preparation

Our phylogenetic footprinting-based workflow for TRN reconstruction initiates with the exploitation of genomic information from closely-related strains to *L. lactis* subsp. *lactis* IO-1. Accordingly, genome and protein sequences, as well as annotation files of *L. lactis* IO-1 and other 74 selected Firmicutes, were downloaded from the NCBI RefSeq database at (ftp://ftp.ncbi.nlm.nih.gov/genomes) to be used as target and reference organisms, respectively.

Selection of the reference genome set directly affects the phylogenetic footprinting procedure primarily in terms of the quantity and quality of the resulting orthologous genes^74^. Hence, four factors were considered for the selection of reference genomes: (i) selected genomes should be relatively close to *L. lactis* subsp. *lactis* IO-1 as phylogenetically-close organisms are more likely to share conserved regulatory mechanisms; (ii) Too closely-related organism should be excluded since analysis of upstream sequences will become less informative and returns many false positives due to the high sequence similarity; (iii) Although relatively close species are favorable, metabolic and physiological diversity should at the same time be maintained in the reference genome set which will omit any bias which can negatively affect the upstream sequences analysis; (iv) Although time-consuming, building the reference genome set should be iterative and genomes can be added or removed from the dataset to reach the improved final results (*i.e*. until reaching significant quantity and quality for the resulting orthologous genes to be used for the motif prediction). Therefore, it is assured that the species which are related enough to detect orthology, but divergent enough to maximize non-alignment noise are picked which will, in turn, reduce the false positive rate of motif discovery.

#### Orthologous promoters identification

Orthologous genes of *L. lactis* subsp. *lactis* IO-1 in the reference genomes were inferred using OrthoFinder2 which has been proved to achieve higher accuracy than all other orthology inference methods^75^. Since genes are organized in operons in prokaryotes, the orthologous relationships inferred between genes were then extended to operon level in a way that operons which share at least one orthologous gene are designated as orthologous operons.

Operons of the target and reference genomes were predicted by both DOOR2 and Operon-mapper tools and the overlapping predicted operons by the two tools were accepted as final result. For those operons being differently predicted by the two tools, the corresponding literature data from the very close strains to *L. lactis* subsp. *lactis* IO-1 were used. DOOR2 predicts operons using a back-end prediction algorithm which is based on the features of intergenic distance, neighborhood conservation, short DNA motifs as well as length ratio between gene pairs and shows a prediction accuracy of 95%^76,77^ and Operon-mapper, on the other hand, takes into account the functional relationships of their protein-coding products^78^, thus, two different and complementary strategies were used for our operon prediction. Further, the 300-bp upstream sequences of all orthologous operons were extracted (orthologous promoters) to be used as the input for the motif discovery step.

#### Identification of phylogenetically conserved *cis*-regulatory motifs

Phylogenetic footprinting is a computational procedure of *cis*-regulatory motif identification in orthologous promoter regions of multiple genomes and is based on the principle that regulatory sequences in promoters are more conserved than the nonfunctional sequences since they evolve at a lower rate^79^. Generally, during this process the set of orthologous promoters are aligned and this alignment is then scanned for conserved motif finding.

Motif discovery was carried out using the motif prediction by phylogenetic footprinting (MP^3^) method^31^. MP^3^ includes a promoter scoring and pruning method and it integrates the six well-known *de novo* motif finding algorithms (Bioprospector^58^, BOBRO^80^, CONSENSUS^81^, CUBIC^82^, MDscan^83^, MEME^84^) as basic motif search engines and is evaluated as very accurate in predicting motifs.

The 300 nucleotides upstream each operon were then used as MP3 inputs with default parameters being used and the top five significant motifs (*p*-value < 0.05) were identified.

#### Regulon prediction

For the clustering of motifs to infer regulons the concept introduced in^31^ was used. According to them, a new scoring system, namely co-regulation score (CRS), is designed based on similarity relationship on operon level. For any pair of operons, A and B, in the target genome, CRS is defined as follows,

$$CRS(A,B)={{\rm{\max }}}_{i,j}\{\frac{{\omega }_{i,j}-\bar{\omega }}{\sigma }\}$$

where $\bar{\omega }$ and *σ* represent the average and variance of similarity scores between any pair of motifs from the two sets of identified motifs in their regulatory promoters. Larger CRS denote the higher probability of co-regulatory relationship these two operons A and B.

With this score, regulons were further inferred via clustering through a heuristic graph model instead of conventionally calculating a pair-wise similarity score for predicted motifs and then identifying regulons through motif clusters^12^.

According to authors, calculating CRS between any pair of operons, shows more accuracy for regulon prediction than other similar scores which are defined through various functional and evolutionary relationships, more details on the approach can be found in^31^.

Available data on regulons of the best-studied closely related strain to *L. lactis* subsp. *lactis* IO-1, namely *L. lactis* subsp. *lactis* IL1403 as well as scattered data on other *L. lactis* subsp. *lactis* strains were used to evaluate the regulon prediction. The concept is that for the most conserved regulation mechanisms such as amino acid biosynthesis, stress response as well as cell wall biosynthesis there should be conserved regulatory mechanisms among the members of same subspecies. Duly, RegPrecise database^85^ which collects manually regulated regulons of prokaryotes, as well as literature data, were implemented. The orthologous *L. lactis* IO-1 genes with all the genes and operons comprising the regulons of other *L. lactis* subsp. *lactis* were inferred. Where significant number of regulons in other *L. lactis* subsp. *lactis* was present with similar members in the final predicted *L. lactis* subsp. *lactis* IO-1 TRN, their orthologous TF in *L. lactis* IO-1 TF repertoire was assigned to corresponding IO-1 regulons (Tables 1 and S4).

The final set of elucidated regulons was used to majorly analyze the special features of *L. lactis* subsp. *lactis* IO-1 such as nisin Z production potential and xylose metabolism.

### Experimental procedure

#### Strains and culture conditions

*L. lactis* strain IO-1 (JCM7638), obtained from the Japan Collection of Microorganisms was grown on its specific chemically defined media (CDM)^86^ supplemented with 1% glucose and incubation temperature of 37 °C in flask in all fermentation experiments as it is the optimum for of *L. lactis* subsp. *lactis* IO-1 without aeration and pH control. When appropriate riboflavin was omitted. *Micrococcus luteus* PTCC 1169 (ATCC 10240), the indicator strain used in nisin bioassay was obtained from Persian Type Culture Collection (PTCC) and grown in nutrient broth/agar medium and grown at 30 °C.

#### Isolation of riboflavin overproducing mutants and analysis of the phenotype’s stability

Roseoflavin is a toxic analogue of riboflavin exposure to which has been reported to cause *L. lactis* strains with spontaneous resistance and this often coincides with a riboflavin-overproducing phenotype^87^. Therefore, exposure to roseoflavin was used to isolate riboflavin overproducing mutants.

*L. lactis* subsp. *lactis* IO-1 was plated at mid exponential growth phase on CDM agar while riboflavin was omitted and supplemented with 100 mg/L roseoflavin to isolate spontaneous roseoflavin-resistant IO-1 mutants. Stability of riboflavin overproduction phenotype was determined by subcluturing the selected mutant strains for 40 generations with periodically measuring extracellular riboflavin concentration.

#### Analysis of riboflavin in culture medium

In stationary phase, the cell free supernatant of the roseoflavin resistant isolates which were grown in CDM (with no riboflavin) was analyzed for riboflavin content. Accordingly, riboflavin concentrations were measured by reverse phase HPLC with fluorescent detection (excitation and emission wavelengths of 440 and 520 nm, respectively) and riboflavin was eluted with a linear gradient of acetonitrile from 3.6% to 30% at pH 3.2^85^. Commercially obtained riboflavin was used for preparing a standard curve for quantitative purposes (Sigma-Aldrich, Germany).

#### Nisin bioassay

For the assay of nisin activity, the growth inhibition of the indicator strain *Micrococcus flavus* ATCC 10240 was determined by the agar well diffusion method described by Tramer *et al*.^88^. Briefly, according to this method, Nutrient agar was inoculated with an appropriate amount of the indicator strain and after being solidified, holes of 7 mm were made to be filled with standard nisin solution and the pretreated broth being studied for its nisin bioactivity. Overnight incubated plates at 30 °C were measured in terms of diameters of the zones of inhibition. The measured diameter was then converted to unit IU/ml according to the standard curve. For making standard nisin solutions, nisin Z (TOKU-E, Osaka, Japan) was used.

#### Statistical analysis

The mean values obtained for nisin Z yields obtained throughout the fermentation analysis were compared using one-way ANOVA in SPSS software (version 19, IBM Co.). Probability (p) values < 0.05 were considered significant.

## Supplementary information

Supplementary materials.

## Data Availability

All data generated or analysed during this study are included in this published article (and its Supplementary Information Files).
