# Supplementary material for: Genome-scale exploration of transcriptional regulation in the nisin Z producer Lactococcus lactis subsp. lactis IO-1
Source: Sci Rep. 2020 Mar 2;10:3787. doi: 10.1038/s41598-020-59731-8 (PMC7051946; doi:10.1038/s41598-020-59731-8)
Supplement: Supplementary file 1 — Supplementary materials. [file 41598_2020_59731_MOESM1_ESM.pdf]

SUPPLEMENTARY FILE FOR:

Genome-scale exploration of transcriptional regulation in the nisin Z producer *Lactococcus lactis* subsp. *lactis* IO-1

Naghmeh Poorinmohammad[1,2], Javad Hamedi*[1,2], Ali Masoudi-Nejad*[3]

1 Department of Microbial Biotechnology, School of Biology and Centre of Excellence in Phylogeny of Living Organisms, College of Science, University of Tehran, Tehran, Iran.

2 Microbial Technology and Products (MTP) Research Center, University of Tehran, Tehran, Iran.

3 Laboratory of Systems Biology and Bioinformatics (LBB), Institute of Biochemistry and Biophysics, University of Tehran, Tehran, Iran.

*Corresponding authors: Javad Hamedi and Ali Masoudi-Nejad

CONTENTS OF THIS SUPPLEMENTARY FILE:

Table S1. Reference genome list (pp2-3)

Table S2. *L. lactis* IO-1 Operon list (pp4-53)

Table S3. *L. lactis* IO-1 Regulon list (pp54-62)

Table S4. Detail information on *L. lactis* IO-1 predicted Regulons. (pp56-86)

Table S5. Detail information on low-score co-regulatory connections of the single-membered regulons of *L. lactis* IO-1. (p 87)

Table S6. Nisin production affected by riboflavin production studied in two conditions (p 88)

Figure S1. Effect of number of selected reference genomes on the final number of predicted regulons in *L. lactis* IO-1 using phylogenetic footprinting approach. (p 89)

Table S1. Reference genome list.

Strain	NCBI assembly accession ID
Acetobacterium woodii DSM 1030	ASM24760v1
Acetohalobium arabaticum DSM 5501	ASM14469v1
Acidaminococcus fermentans DSM 20731	ASM2530v1
Aerococcus urinae ACS-120-V-Col10a	ASM19320v1
Alicyclobacillus acidocaldarius subsp. acidocaldarius DSM 446	ASM2428v1
Alkaliphilus metalliredigens QYMF	ASM1698v1
Ammonifex degensii KC4	ASM2460v1
Amphibacillus xylanus NBRC 15112	ASM30716v1
Anaerococcus prevotii DSM 20548	ASM2410v1
Anoxybacillus flavithermus WK1	ASM1904v1
Bacillus subtilis subsp. subtilis str. 168	ASM904v1
Brevibacillus brevis NBRC 100599	ASM1016v1
Butyrivibrio proteoclasticus B316	ASM14503v1
Caldicellulosiruptor obsidiansis OB47	ASM14521v1
Carboxydothermus hydrogenoformans Z-2901	ASM1286v1
Carnobacterium maltaromaticum LMA28	ASM31797v2
Clostridioides difficile 630	ASM920v1
Clostridium acetobutylicum ATCC 824	ASM876v1
Coprothermobacter proteolyticus DSM 5265	ASM2094v1
Dehalobacter sp. DCA	ASM30577v1
Desulfitobacterium hafniense DCB-2	ASM2192v1
Desulfosporosinus meridiei DSM 13257	ASM23138v3
Desulfotomaculum acetoxidans DSM 771	ASM2420v1
Enterococcus faecalis V583	ASM778v1
Erysipelothrix rhusiopathiae str. Fujisawa	ASM27008v1
Ethanoligenens harbinense YUAN-3	ASM17811v2
Eubacterium limosum KIST612	ASM15224v2
Exiguobacterium antarcticum B7	ASM29943v1
Fictibacillus phosphorivorans	ASM162970v2
Filifactor alocis ATCC 35896	ASM16389v2
Finegoldia magna ATCC 29328	ASM1018v1
Geobacillus thermodenitrificans NG80-2	ASM1574v1
Halanaerobium praevalens DSM 2228	ASM16546v1
Halobacillus halophilus DSM 2266	ASM28451v1
Halobacteroides halobius DSM 5150	ASM32862v1
Halothermothrix orenii H 168	ASM2048v1
dialister pneumosintes	ASM171750v1
Kyrpidia tusciae DSM 2912	ASM9290v1
Lactobacillus plantarum WCFS1	ASM20385v3
Lactococcus lactis subsp. lactis Il1403	ASM686v1
Lactococcus lactis subsp. lactis KF147	ASM2504v1
Lactococcus lactis subsp. lactis CV56	ASM19270v1
Leuconostoc mesenteroides subsp. mesenteroides ATCC 8293	ASM1444v1
Listeria monocytogenes EGD-e	ASM19603v1

Lysinibacillus fusiformis	ASM72477v3
Macrococcus caseolyticus JCSC5402	ASM1058v1
Mageeibacillus indolicus UPII9-5	ASM2522v1
Mahella australiensis 50-1 BON	ASM21325v1
Moorella thermoacetica ATCC 39073	ASM1310v1
Oceanobacillus iheyensis HTE831	ASM1124v1
Oenococcus oeni PSU-1	ASM1438v1
Oscillibacter valericigenes Sjm18-20	ASM28357v1
Paenibacillus sp. JDR-2	ASM2358v1
Parvimonas micra	ASM80029v1
Pediococcus pentosaceus ATCC 25745	ASM1450v1
Pelotomaculum thermopropionicum SI	ASM1056v1
Roseburia hominis A2-183	ASM22534v1
Ruminococcus albus 7 = DSM 20455	ASM17963v2
Selenomonas ruminantium subsp. lactilytica TAM6421	ASM28409v1
Solibacillus silvestris	ASM27132v1
Staphylococcus aureus subsp. aureus NCTC 8325	ASM1342v1
Streptococcus suis BM407	ASM2674v1
Symbiobacterium thermophilum IAM 14863	ASM990v1
Syntrophomonas wolfei subsp. wolfei str. Goettingen G311	ASM1472v1
Tepidanaerobacter acetatoxydans Re1	ASM32876v2
Terribacillus aidingensis	ASM72536v1
Tetragenococcus halophilus NBRC 12172	ASM28361v1
Thermacetogenium phaeum DSM 12270	ASM30593v1
Thermoanaerobacter italicus Ab9	ASM2564v1
Thermobacillus composti KWC4	ASM22770v3
Thermodesulfobium narugense DSM 14796	ASM21239v1
Thermosediminibacter oceani DSM 16646	ASM14464v1
Veillonella parvula DSM 2008	ASM2494v1
Weissella koreensis KACC 15510	ASM21980v1

Table S2. *L. lactis* IO-1 Operon list.

OperonID	Locus tag	Refseq Locus tag	Product
Operon_1	lilo_0001	LILO_RS00010	replication initiation protein DnaA
Operon_2	lilo_0002	LILO_RS00015	DNA polymerase III, beta chain
Operon_3	lilo_0003	LILO_RS00020	ATP-dependent nuclease subunit B
	lilo_0004	LILO_RS00025	ATP-dependent nuclease subunit A
	lilo_0005	LILO_RS00030	hypothetical protein
Operon_4	lilo_0006	LILO_RS00035	transcription regulator
Operon_1396	lilo_0007	LILO_RS00040	GTP-dependent nucleic acid-binding protein translation factor
Operon_1397	lilo_0008	LILO_RS00045	Cro/CI family transcriptional regulator
Operon_5	lilo_0010	LILO_RS00060	hypothetical protein
	lilo_0011	LILO_RS00065	peptidyl-tRNA hydrolase
	lilo_0012	LILO_RS00070	transcription-repair coupling factor
Operon_6	lilo_0013	LILO_RS00075	hypothetical protein
Operon_7	lilo_0014	LILO_RS00080	S4 domain-containing RNA-binding protein
	lilo_0015	LILO_RS00085	septum formation initiator
	lilo_0016	LILO_RS00090	hypothetical protein
Operon_8	lilo_0017	LILO_RS00095	beta-lactamase-type transpeptidase
	lilo_0018	LILO_RS00100	cell cycle protein/ tRNA(Ile)-lysidine synthetase
	lilo_0019	lilo_0019	hypoxantine-guanine phosphorybosyltransferase
Operon_9	lilo_0020	LILO_RS00110	cell division protein FtsH
Operon_10	lilo_0021	LILO_RS00155	PTS system, mannitol-specific IIBC component
	lilo_0022	LILO_RS00160	DeoR family transcriptional regulator
	lilo_0023	LILO_RS00165	mannitol-specific PTS system IIA component
	lilo_0024	LILO_RS00170	mannitol-1-phosphate 5-dehydrogenase
Operon_12	lilo_0025	lilo_0025	hypothetical protein
Operon_13	lilo_0027	LILO_RS00180	hypothetical protein
Operon_14	lilo_0028	LILO_RS00185	putative sulphate transporter
Operon_15	lilo_0029	LILO_RS00190	GNAT family acetyltransferase
Operon_16	lilo_0030	LILO_RS00200	aromatic amino acid specific aminotransferase
Operon_17	lilo_0031	LILO_RS00205	DNA repair protein RecO
Operon_18	lilo_0032	lilo_0032	teichoic acid biosynthesis protein B
Operon_19	lilo_0033	LILO_RS00210	pyruvate dehydrogenase complex dihydrolipoamide acetyltransferase
Operon_20	lilo_0034	LILO_RS00215	pyruvate dehydrogenase complex dihydrolipoamide acetyltransferase
	lilo_0035	LILO_RS00220	pyruvate dehydrogenase E1 component subunit beta
	lilo_0036	LILO_RS00225	pyruvate dehydrogenase E1 component subunit alpha
	lilo_0037	LILO_RS00230	lipoate-protein ligase
Operon_21	lilo_0038	LILO_RS00235	glycerophosphoryl diester phosphodiesterase
Operon_22	lilo_0039	LILO_RS00240	HD family metal-dependent phosphohydrolase
Operon_23	lilo_0040	LILO_RS00245	tryptophanyl-tRNA synthetase
Operon_24	lilo_0041	LILO_RS00250	osmotically inducible protein
Operon_25	lilo_0042	LILO_RS00255	amino acid permease
Operon_26	lilo_0044	LILO_RS00265	fatty acid/phospholipid synthesis protein
Operon_27	lilo_0045	LILO_RS00270	HAD superfamily hydrolase

Operon_28	lilo_0046	LILO_RS00275	ABC transporter ATP binding protein
Operon_29	lilo_0047	LILO_RS00280	O-acetylhomoserine (thiol)-lyase
Operon_30	lilo_0048	LILO_RS00285	short-chain type dehydrogenase
Operon_31	lilo_0049	LILO_RS00290	universal stress protein A
Operon_32	lilo_0050	LILO_RS00295	hypothetical protein
	lilo_0051	LILO_RS00300	alpha/beta hydrolase
	lilo_0052	LILO_RS00305	glyoxalase family protein
	lilo_0053	LILO_RS00310	flavin reductase family protein
Operon_33	lilo_0054	LILO_RS00315	50S ribosomal protein L33
	lilo_0055	LILO_RS00320	50S ribosomal protein L32
Operon_34	lilo_0056	LILO_RS00325	lead, cadmium, zinc, mercury transporting ATPase
Operon_35	lilo_0057	LILO_RS00330	chromosome partitioning protein
	lilo_0058	LILO_RS00335	chromosome segregation helicase
	lilo_0059	LILO_RS00340	acetyl transferase
Operon_36	lilo_0060	LILO_RS00345	hypothetical protein
	lilo_0061	LILO_RS00350	ABC transporter ATP binding protein
Operon_37	lilo_0062	LILO_RS00355	hypothetical protein
	lilo_0063	LILO_RS00360	hypothetical protein
	lilo_0064	LILO_RS00365	ribosomal protein L11 methyltransferase
Operon_38	lilo_0065	LILO_RS00370	hypothetical protein
	lilo_0066	LILO_RS00375	predicted xylanase/chitin deacetilase
Operon_39	lilo_0067	LILO_RS00380	GTP pyrophosphokinase
Operon_40	lilo_0069	LILO_RS00395	hypothetical protein
	lilo_0070	LILO_RS00400	HAD superfamily hydrolase
Operon_41	lilo_0071	LILO_RS00405	cationic amino acid transporter
Operon_42	lilo_0072	LILO_RS00410	1-acyl-sn-glycerol-3-phosphate acyltransferase
Operon_43	lilo_0073	LILO_RS00415	MarR family transcriptional regulator
	lilo_0074	LILO_RS00420	acyl carrier protein phosphodiesterase
Operon_44	lilo_0075	LILO_RS00425	preprotein translocase SecA subunit
Operon_45	lilo_0076	LILO_RS00430	phospho-2-dehydro-3-deoxyheptonate aldolase
Operon_46	lilo_0077	LILO_RS00435	phosphocarrier protein HPr
	lilo_0078	LILO_RS00440	phosphotransferase system, enzyme I/ phosphoenolpyruvate-protein phosphotransferase
Operon_47	lilo_0079	LILO_RS00445	hypothetical protein
Operon_48	lilo_0080	LILO_RS00450	hypothetical protein
Operon_49	lilo_0081	LILO_RS00460	alpha/beta hydrolase
	lilo_0082	lilo_0082	hypothetical protein
Operon_50	lilo_0083	LILO_RS00470	SugE protein
Operon_51	lilo_0084	LILO_RS00475	multidrug efflux transporter
Operon_52	lilo_0085	LILO_RS00480	glycosil transferase
	lilo_0086	LILO_RS00485	hypothetical protein
	lilo_0068	LILO_RS00385	glycosil transferase
Operon_53	lilo_0087	LILO_RS00495	argininosuccinate synthase
	lilo_0088	LILO_RS00500	argininosuccinate lyase
Operon_54	lilo_0089	LILO_RS00505	multidrug resistance efflux pump
Operon_55	lilo_0090	LILO_RS00510	TetR family transcriptional regulator
Operon_56	lilo_0091	LILO_RS00515	ribonuclease P protein component

	lilo_0092	LILO_RS00520	preprotein translocase subunit YidC
Operon_57	lilo_0093	LILO_RS00525	putative spoIIIJ-associated protein
Operon_58	lilo_0094	LILO_RS00530	50S ribosomal protein L34
Operon_59	lilo_0095	LILO_RS00535	aldo/keto reductase family enzyme
Operon_60	lilo_0096	LILO_RS00540	transcription regulator
Operon_61	lilo_0097	LILO_RS00545	hypothetical protein
	lilo_0098	lilo_0098	hypothetical protein
	lilo_0099	LILO_RS00555	hypothetical protein
Operon_62	lilo_0100	LILO_RS00560	hypothetical protein
	lilo_0101	LILO_RS00565	putative Hollyday junction resolvase
Operon_63	lilo_0102	LILO_RS00570	hypothetical protein
Operon_64	lilo_0103	LILO_RS00575	hypothetical protein
Operon_65	lilo_0104	LILO_RS00580	carbonyl reductase
	lilo_0105	LILO_RS00585	hypothetical protein
	lilo_0106	LILO_RS00590	oxidoreductase
Operon_66	lilo_0107	LILO_RS00595	TetR family transcriptional regulator
Operon_67	lilo_0108	LILO_RS00600	1,4-alpha-glucan branching enzyme
Operon_68	lilo_0109	LILO_RS00605	hypothetical protein
Operon_69	lilo_0110	LILO_RS00610	hypothetical protein
Operon_70	lilo_0111	LILO_RS00615	cell surface protein
Operon_71	lilo_0112	LILO_RS00620	hypothetical protein
Operon_72	lilo_0113	lilo_0113	cell surface protein
Operon_73	lilo_0114	LILO_RS00630	hypothetical protein
Operon_74	lilo_0115	LILO_RS00635	transcriptional regulator, xre family
Operon_75	lilo_0116	LILO_RS00645	queuine tRNA-ribosyltransferase
Operon_76	lilo_0117	LILO_RS00650	hypothetical protein
Operon_77	lilo_0118	LILO_RS00655	MF superfamily transporter
Operon_78	lilo_0119	LILO_RS00660	hypothetical protein
Operon_79	lilo_0120	LILO_RS00665	DNA/RNA non-specific endonuclease
Operon_80	lilo_0121	LILO_RS00670	hypothetical protein
	lilo_0122	LILO_RS00675	hypothetical protein
Operon_81	lilo_0123	LILO_RS00680	heme/copper-type cytochrome/quinol oxidase subunit 1
Operon_82	lilo_0124	LILO_RS00685	aspartate aminotransferase
Operon_83	lilo_0125	LILO_RS00690	transcriptional regulator
Operon_84	lilo_0126	LILO_RS00695	glutamyl-tRNA (Gln) amidotransferase subunit C
	lilo_0127	LILO_RS00700	Glu-tRNA amidotransferase subunit A
	lilo_0128	LILO_RS00705	hypothetical protein
	lilo_0129	LILO_RS00710	Glu-tRNA amidotransferase subunit B
Operon_85	lilo_0130	LILO_RS00715	amidase
Operon_86	lilo_0131	LILO_RS00720	MatE family Na+ driven multidrug efflux pump
Operon_87	lilo_0132	LILO_RS00725	cold shock protein E
Operon_88	lilo_0133	LILO_RS00730	5-formyltetrahydrofolate cyclo-ligase
	lilo_0134	LILO_RS00735	rhomboid family intramembrane serine protease
Operon_89	lilo_0135	LILO_RS00740	hypothetical protein
Operon_90	lilo_0136	LILO_RS00745	GNAT family acetyltransferase
Operon_91	lilo_0137	LILO_RS00750	hypothetical protein

Operon_92	lilo_0138	LILO_RS00755	cellobiose-specific PTS system IIC component
Operon_93	lilo_0139	LILO_RS00760	hypothetical protein
Operon_94	lilo_0140	LILO_RS00770	beta-glucosidase A
	lilo_0141	LILO_RS00775	deoxyuridine 5'-triphosphate nucleotidhydrolase
Operon_95	lilo_0142	LILO_RS00780	5'-nucleotidase
	lilo_0143	LILO_RS00785	hypothetical protein
Operon_96	lilo_0144	LILO_RS00790	radical SAM protein
Operon_97	lilo_0145	LILO_RS00795	oxidoreductase
Operon_98	lilo_0146	LILO_RS00800	prenyl transferase
Operon_99	lilo_0147	LILO_RS00805	1,4-dihydroxy-2-naphthoate polyprenyltransferase
Operon_100	lilo_0148	LILO_RS00810	CAAX amino terminal protease family protein
Operon_101	lilo_0149	LILO_RS00815	hypothetical protein
	lilo_0150	LILO_RS00820	ferrous ion transport protein B
	lilo_0151	LILO_RS00825	ferrous ion transport protein A
Operon_103	lilo_0152	LILO_RS00830	putative peptide methionine sulfoxide reductase
Operon_104	lilo_0153	LILO_RS00835	dihydroxyacetone kinase family protein
	lilo_0154	LILO_RS00840	Gls24 family general stress protein
Operon_105	lilo_0155	LILO_RS00845	50S ribosomal protein L28P
Operon_106	lilo_0156	LILO_RS00850	hypothetical protein
Operon_107	lilo_0157	LILO_RS00855	glucose-1-phosphate thymidylyltransferase
	lilo_0158	LILO_RS00860	hypothetical protein
	lilo_0159	LILO_RS00865	dTDP-4-keto-6-deoxyglucose-3,5-epimerase
	lilo_0160	LILO_RS00870	hypothetical protein
	lilo_0161	LILO_RS00875	dTDP-glucose 4,6-dehydratase
	lilo_0162	LILO_RS00880	predicted lactoylglutathione lyase
	lilo_0163	LILO_RS00885	dTDP-L-rhamnose synthase
Operon_108	lilo_0164	LILO_RS00890	rhamnosyltransferase
	lilo_0165	LILO_RS00895	rhamnosyltransferase
	lilo_0166	LILO_RS00900	polysaccharide ABC transporter permease protein
	lilo_0167	LILO_RS00905	polysaccharide ABC transporter ATP-binding protein
	lilo_0168	LILO_RS00910	predicted membrane protein
	lilo_0169	LILO_RS00915	glycosyltransferase/ beta-1,3-N-acetylglucosaminyltransferase
	lilo_0170	LILO_RS00920	lipopolysaccharide biosynthesis protein/ bifunctional alpha-L-Rha alpha-1,2-L-rhamnosyltransferase/alpha-L-Rha alpha-1,3-L-rhamnosyltransferase
Operon_109	lilo_1422	lilo_1422	hypothetical protein
	lilo_1423	LILO_RS07370	hypothetical protein
	lilo_0009	LILO_RS00055	glycerophosphoryl diester phosphodiesterase
	lilo_0171	LILO_RS00925	glycosyltransferase
	lilo_0172	LILO_RS00935	glycosyl transferase
	lilo_1347	LILO_RS06990	prophage pi3 protein 1
	lilo_0173	LILO_RS00940	similar to glycosyl transferase
	lilo_0174	LILO_RS00945	LPS biosynthesis protein
	lilo_1285	LILO_RS06695	hypothetical protein
	lilo_0175	LILO_RS00955	UDP-glucose 4-epimerase
Operon_110	lilo_0176	LILO_RS00960	putative galactofuranose transferase

	lilo_0177	LILO_RS00965	UDP-galactopyranose mutase
	lilo_0178	LILO_RS00970	polysaccharide transporter, PST family
	lilo_0179	LILO_RS00975	possible surface protein
Operon_111	lilo_0180	LILO_RS00985	hypothetical protein
Operon_112	lilo_0181	LILO_RS00990	IMP dehydrogenase
Operon_113	lilo_0182	LILO_RS00995	GTP-binding protein
	lilo_0183	LILO_RS01000	NUDIX family hydrolase
Operon_114	lilo_0184	LILO_RS01005	GTP-binding protein HflX
	lilo_0185	LILO_RS01010	CRS1/YhbY family RNA binding protein
	lilo_0186	LILO_RS01015	nicotinate-nucleotide adenylyltransferase
	lilo_0187	LILO_RS01020	HAD superfamily hydrolase
	lilo_0188	LILO_RS01025	GNAT family acetyltransferase
Operon_115	lilo_0189	LILO_RS01030	hypothetical protein
Operon_116	lilo_0190	LILO_RS01035	methyltransferase
	lilo_0191	LILO_RS01040	hypothetical protein
Operon_117	lilo_0192	LILO_RS01045	hypothetical protein
Operon_118	lilo_0193	LILO_RS01050	hypothetical protein
Operon_119	lilo_0194	LILO_RS01055	hypothetical protein
	lilo_0195	LILO_RS01060	uracil-DNA glycosilase
Operon_120	lilo_0196	LILO_RS01065	putative zinc-binding dehydrogenase
Operon_121	lilo_0197	LILO_RS01070	TetR family transcriptional regulator
Operon_122	lilo_0198	LILO_RS01075	transporter
Operon_123	lilo_0199	LILO_RS01080	MerR family transcriptional regulator
Operon_124	lilo_0200	LILO_RS01085	30S ribosomal protein S21
Operon_125	lilo_0201	LILO_RS01090	HAD superfamily hydrolase
Operon_126	lilo_0202	LILO_RS01095	phosphoglycerate kinase
Operon_127	lilo_0203	LILO_RS01100	dihydroxyacetone kinase
Operon_128	lilo_0204	LILO_RS01105	TetR family transcriptional regulator
Operon_129	lilo_0205	LILO_RS01110	dihydroxyacetone kinase
	lilo_0206	LILO_RS01115	dihydroxyacetone kinase
	lilo_0207	LILO_RS01120	hypothetical protein
	lilo_0208	LILO_RS01125	glycerol uptake facilitator
	lilo_0255	LILO_RS01385	glycerol uptake facilitator
Operon_130	lilo_0209	LILO_RS01130	dipeptidase
Operon_131	lilo_0210	LILO_RS01135	ABC transporter ATP binding protein
	lilo_0211	LILO_RS01140	ABC transporter permease protein
Operon_132	lilo_0212	LILO_RS01145	SAM-dependent methyltransferase
Operon_133	lilo_0213	LILO_RS01150	fructose-1,6-bisphosphatase
Operon_134	lilo_0214	LILO_RS01160	NAD(FAD)-utilizing dehydrogenase
Operon_135	lilo_0215	LILO_RS01165	carbohydrate kinase
Operon_136	lilo_0216	LILO_RS01170	hypothetical protein
Operon_137	lilo_0217	LILO_RS01175	ABC transporter ATP binding protein
	lilo_0218	LILO_RS01180	ABC transporter ATP binding and permease protein
Operon_138	lilo_0219	LILO_RS01185	hypothetical protein
Operon_139	lilo_0220	LILO_RS01190	oxidoreductase
Operon_140	lilo_0221	LILO_RS01195	autoinducer-2 production protein/ S-ribosylhomocysteinase

Operon_141	lilo_0222	LILO_RS01200	transcription activator
	lilo_0223	LILO_RS01205	oxidoreductase/ aldo/keto reductase
Operon_142	lilo_0224	LILO_RS01210	amidase/ cysteine hydrolase, isochorismatse family
Operon_143	lilo_0225	LILO_RS01215	hypothetical protein
	lilo_0226	LILO_RS01220	hypothetical protein
Operon_144	lilo_0227	LILO_RS01225	N-acetylmuramidase
Operon_145	lilo_0228	LILO_RS01230	anaerobic ribonucleoside-triphosphate reductase
	lilo_0229	LILO_RS01235	anaerobic ribonucleoside-triphosphate reductase activating protein
Operon_146	lilo_0230	LILO_RS01240	hypothetical protein
Operon_147	lilo_0231	LILO_RS01245	enolase (phosphopyruvate hydratase)
Operon_149	lilo_0232	LILO_RS01255	cobalt ABC transporter ATP-binding protein
	lilo_0233	LILO_RS01265	cobalt ABC transporter ATP-binding protein
	lilo_0234	LILO_RS01270	cobalt ABC transporter permease
	lilo_0235	LILO_RS01275	thiol-disulfide isomerase and thioredoxin
Operon_150	lilo_0236	LILO_RS01285	putative 2,3,4,5-tetrahydropyridine-2-carboxylate N-succinyltransferase
Operon_151	lilo_0237	LILO_RS01290	putative N-acetyldiaminopimelate deacetylase
Operon_152	lilo_0238	LILO_RS01295	endo-1,4-beta-xylanase D
Operon_153	lilo_0239	LILO_RS01310	30S ribosomal protein S4
Operon_154	lilo_0240	LILO_RS01315	transcription accessory protein (S1 RNA binding domain)
Operon_155	lilo_0241	LILO_RS01320	SprT-like protein
Operon_156	lilo_0242	LILO_RS01325	adenosine deaminase
Operon_157	lilo_0243	lilo_0243	hypothetical protein
Operon_158	lilo_0244	LILO_RS01335	mRNA degradation ribonuclease, metallo-beta-lactamase superfamily
Operon_159	lilo_0245	LILO_RS01340	predicted RNA binding protein, contains RRM domain
Operon_160	lilo_0246	lilo_0246	hypothetical protein
Operon_161	lilo_0247	LILO_RS01345	peptidase family M22 non-proteolytic protein
	lilo_0248	LILO_RS01350	acetyltransferase
	lilo_0249	LILO_RS01355	putative ribosomal-protein-alanine acetyltransferase
	lilo_0250	LILO_RS01360	O-sialoglycoprotein endopeptidase
	lilo_0251	LILO_RS01365	hypothetical protein
Operon_162	lilo_0252	LILO_RS11715	hypothetical protein
	lilo_0253	LILO_RS01375	cellobiose-specific PTS system IIC component
Operon_163	lilo_0254	LILO_RS01380	transcriptional regulator
Operon_164	lilo_0256	LILO_RS01390	hypothetical protein
Operon_165	lilo_0257	LILO_RS01395	phosphonate ABC transporter substrate-binding protein
Operon_166	lilo_0258	LILO_RS01400	phosphonate ABC transporter ATP-binding protein
	lilo_0259	LILO_RS01405	phosphonate ABC transporter permease protein
	lilo_0260	LILO_RS01410	phosphonate ABC transporter permease protein
	lilo_0261	LILO_RS01415	2,3-cyclic-nucleotide 2-phosphodiesterase/3-nucleotidase
Operon_167	lilo_0262	lilo_0262	hypothetical protein
	lilo_0263	LILO_RS01420	thioredoxin peroxidase
Operon_168	lilo_0264	LILO_RS01425	aminopeptidase N

Operon_169	lilo_0265	LILO_RS01430	multidrug-efflux transporter
Operon_170	lilo_0266	LILO_RS01435	MarR family transcriptional regulator
	lilo_0267	LILO_RS01440	co/Zn/cd efflux protein
Operon_171	lilo_0268	LILO_RS01445	PadR family transcriptional regulator
Operon_172	lilo_0269	LILO_RS01450	ABC transporter ATP binding and permease protein
	lilo_0270	LILO_RS01455	ABC transporter ATP binding and permease protein
Operon_173	lilo_0271	LILO_RS01460	UDP-N-acetylglucosamine 1-carboxyvinyltransferase
	lilo_0272	LILO_RS01465	hypothetical protein
Operon_174	lilo_0273	LILO_RS01470	Rgg/GadR/MutR family transcriptional regulator
Operon_175	lilo_0274	LILO_RS01475	homoserine dehydrogenase
	lilo_0275	LILO_RS01480	ATPase component of ABC transporter with duplicatedATPase domain
	lilo_0276	LILO_RS01485	hypothetical protein
	lilo_0277	LILO_RS01490	Na+-driven multidrug efflux pump
	lilo_0278	LILO_RS01495	UDP-galactopyranose mutase
Operon_176	lilo_0279	LILO_RS01500	ABC transporter ATP binding and permease protein
Operon_177	lilo_0280	LILO_RS01505	hypothetical protein
	lilo_0281	LILO_RS01510	putative biotin biosynthesis protein
Operon_178	lilo_0282	LILO_RS01515	transcriptional regulator
Operon_179	lilo_0283	LILO_RS01520	thiamine transporter
Operon_180	lilo_0284	LILO_RS01525	outer membrane lipoprotein precursor
	lilo_0285	LILO_RS01530	outer membrane lipoprotein precursor
	lilo_0286	LILO_RS01535	outer membrane lipoprotein precursor
	lilo_0287	LILO_RS01540	outer membrane lipoprotein precursor
	lilo_0288	LILO_RS01545	amino acid ABC transporter ATP binding protein
	lilo_0289	LILO_RS01550	amino acid ABC trasporter permease protein
Operon_181	lilo_0290	LILO_RS01555	cobalt ABC transporter permease
	lilo_0291	LILO_RS01560	cobalt ABC transporter ATP-binding protein
	lilo_0292	LILO_RS01565	cobalt ABC transporter permease
Operon_182	lilo_0293	LILO_RS01570	Cro/CI family transcriptional regulator
	lilo_0295	LILO_RS01580	ferrichrome ABC transporter permease protein
	lilo_0296	LILO_RS01585	ferrichrome ABC transporter permease protein
	lilo_0297	LILO_RS01590	ferrichrome ABC transporter substrate binding protein
	lilo_0298	LILO_RS01595	fhu operon transcription regulator
Operon_185	lilo_0299	LILO_RS01600	sugar efflux transporter
	lilo_0300	LILO_RS01605	oxidoreductase
Operon_186	lilo_0301	LILO_RS01610	MarR family transcriptional regulator
	lilo_0302	LILO_RS01615	glyoxalase family protein
Operon_187	lilo_0303	LILO_RS01620	phosphoglycerate mutase
Operon_188	lilo_0304	LILO_RS01625	alkyl hydroperoxide reductase
	lilo_0305	LILO_RS01630	alkyl hydroperoxide reductase
Operon_189	lilo_0306	LILO_RS01635	penicillin-binding protein 2B
Operon_190	lilo_0307	LILO_RS01640	RecM protein
	lilo_0308	LILO_RS01645	D-alanyl-alanine synthetase A
	lilo_0309	LILO_RS01650	UDP-N-acetylmuramoylalanyl-D-glutamyl-2,6-diaminopimelate--D-alanyl-D-alanine ligase
Operon_192	lilo_0310	LILO_RS01655	oligopeptide ABC transporter substrate binding

			protein
	lilo_0311	LILO_RS01660	oligopeptide ABC transporter substrate binding protein
	lilo_0312	LILO_RS01665	dipeptide transport system permease protein DppB
	lilo_0313	LILO_RS01670	ABC-type dipeptide/oligopeptide/nickel transport system, permease component
	lilo_0314	LILO_RS01675	oligopeptide ABC transporter ATP binding protein
	lilo_0315	LILO_RS01680	oligopeptide ABC transporter ATP binding protein
Operon_193	lilo_0316	LILO_RS01685	peptide chain release factor RF-3
Operon_194	lilo_0317	LILO_RS01690	ATP-dependent RNA helicase
Operon_195	lilo_0318	LILO_RS01695	GTP-binding protein Era
Operon_196	lilo_0319	LILO_RS01700	asparagine synthetase B
Operon_197	lilo_0320	LILO_RS01705	formamidopyrimidine-DNA glycosylase
Operon_198	lilo_0321	LILO_RS01710	DNA-dependent recombination protein RecA
Operon_199	lilo_0322	LILO_RS01715	amino acid permease
	lilo_0323	LILO_RS01720	amino acid permease
Operon_200	lilo_0324	LILO_RS01725	hypothetical protein
	lilo_0325	LILO_RS01730	hypothetical protein
Operon_201	lilo_0326	LILO_RS01735	dithiol-disulfide isomerase
Operon_202	lilo_0327	LILO_RS01740	hypothetical protein
Operon_203	lilo_0328	LILO_RS01745	hypothetical protein
Operon_204	lilo_0329	LILO_RS01750	GTP pyrophosphokinase
	lilo_0330	LILO_RS01755	probable inorganic polyphosphate/ATP-NAD kinase
	lilo_0331	LILO_RS01760	pseudouridine synthase
Operon_205	lilo_0332	LILO_RS01765	peptidyl-prolyl cis-trans isomerase
Operon_206	lilo_0333	LILO_RS01770	lysine specific permease
Operon_207	lilo_0334	LILO_RS01775	NADPH-dependent FMN reductase
	lilo_0335	LILO_RS01780	thiamine biosynthesis lipoprotein
Operon_208	lilo_0336	LILO_RS01785	lysyl-tRNA synthetase
Operon_209	lilo_0337	LILO_RS01790	LysR family transcription regulator
Operon_210	lilo_0338	LILO_RS01795	predicted membrane protein
Operon_211	lilo_0339	LILO_RS01800	L-lactate dehydrogenase
Operon_212	lilo_0340	LILO_RS01805	LysR family transcription regulator
Operon_213	lilo_0341	LILO_RS01810	C4-dicarboxylate transporter/malic acid transporter
Operon_214	lilo_0342	LILO_RS01815	thiamine biosynthesis protein ThiI
Operon_215	lilo_0343	LILO_RS01820	efflux pump antibiotic resistance protein
Operon_216	lilo_0344	LILO_RS01825	acyl carrier protein phosphodiesterase
Operon_217	lilo_0345	LILO_RS01830	osmotically inducible protein C
Operon_218	lilo_0346	LILO_RS01835	Na+/H+ antiporter
Operon_219	lilo_0347	LILO_RS01840	hypothetical protein
Operon_220	lilo_0348	LILO_RS01845	tyrosyl-tRNA synthetase
Operon_221	lilo_0349	LILO_RS01850	penicillin-binding protein 1B
Operon_222	lilo_0350	LILO_RS01855	glutamyl aminopeptidase
Operon_223	lilo_0351	LILO_RS01860	hypothetical protein
	lilo_0352	LILO_RS01865	thioredoxin H-type
Operon_224	lilo_0353	LILO_RS01870	tRNA binding domain protein
Operon_225	lilo_0354	LILO_RS01875	NADH oxidase

Operon_226	lilo_0355	LILO_RS01880	single-strand binding protein
Operon_227	lilo_0356	LILO_RS01885	10 KD chaperonin
Operon_228	lilo_0357	LILO_RS01890	60 KD chaperonin
Operon_229	lilo_0358	LILO_RS01895	beta-lactamase superfamily Zn-dependent hydrolase
Operon_230	lilo_0359	LILO_RS01900	two-component sensor histidine kinase
	lilo_0360	LILO_RS01905	two-component response regulator
Operon_231	lilo_0361	LILO_RS01910	dTMP kinase
Operon_232	lilo_0362	LILO_RS01915	DNA polymerase III, delta' subunit
	lilo_0363	LILO_RS01920	phosphorelay inhibitor
	lilo_0364	LILO_RS01925	DNA replication initiation-control protein
	lilo_0365	LILO_RS01930	tetrapyrrole methylase
Operon_233	lilo_0366	LILO_RS01935	hypothetical protein
Operon_234	lilo_0367	LILO_RS01940	mevalonate kinase
Operon_235	lilo_0368	LILO_RS01945	diphosphomevalonate decarboxylase
	lilo_0369	LILO_RS01950	phosphomevalonate kinase
	lilo_0370	LILO_RS01955	isopentenyl-diphosphate delta-isomerase
Operon_236	lilo_0371	LILO_RS01960	superoxide dismutase
Operon_237	lilo_0372	LILO_RS01965	carbon starvation protein
Operon_238	lilo_0373	LILO_RS01970	acyl carrier protein phosphodiesterase
Operon_239	lilo_0374	LILO_RS01975	similar to transcription regulator
Operon_240	lilo_0375	LILO_RS01980	ATP-dependent RNA helicase
	lilo_0376	LILO_RS01985	hypothetical protein
Operon_241	lilo_0377	LILO_RS01990	RpiR family transcriptional regulator
Operon_242	lilo_0378	LILO_RS01995	cellobiose-specific PTS system IIB component
Operon_243	lilo_0379	LILO_RS02000	cellobiose-specific PTS system IIA component
Operon_244	lilo_0380	LILO_RS02005	RpiR family transcriptional regulator
Operon_245	lilo_0381	LILO_RS02010	cellobiose-specific PTS system IIC component
	lilo_0382	LILO_RS02015	6-phospho-beta-glucosidase
Operon_246	lilo_0383	LILO_RS02020	hypothetical protein
Operon_247	lilo_0384	LILO_RS02030	DNA ligase
Operon_248	lilo_0385	LILO_RS02035	transcription regulator
Operon_249	lilo_0386	LILO_RS02040	multiple sugar ABC transporter ATP-binding protein
Operon_250	lilo_0387	LILO_RS02045	pyruvate ferredoxin oxidoreductase
Operon_251	lilo_0388	LILO_RS02050	hypothetical protein
Operon_252	lilo_0389	LILO_RS02055	hypothetical protein
	lilo_0390	LILO_RS02060	phosphoglucomutase
Operon_253	lilo_0391	LILO_RS02065	GntR family transcriptional regulator
Operon_254	lilo_0392	LILO_RS02070	PTS system, beta-glucosides-specific IIA component
Operon_255	lilo_0393	LILO_RS02075	beta-glucoside-specific PTS system IIBC component
	lilo_0394	LILO_RS02080	glycosyl hydrolase, putative
Operon_256	lilo_0395	LILO_RS02085	beta-phosphoglucomutase
Operon_257	lilo_0396	LILO_RS02090	sugar hydrolase
Operon_258	lilo_0397	LILO_RS02095	hypothetical protein
Operon_259	lilo_0398	LILO_RS02105	ATP/GTP hydrolase
	lilo_0399	LILO_RS02110	GNAT family acetyltransferase
Operon_260	lilo_0400	LILO_RS02115	LytR family transcriptional regulator

Operon_261	lilo_0401	LILO_RS02125	tRNA pseudouridine synthase A
	lilo_0402	LILO_RS02130	phosphomethylpyrimidine kinase
	lilo_0403	LILO_RS02135	hypothetical protein
	lilo_0404	LILO_RS02140	hypothetical protein
	lilo_0405	LILO_RS02145	serine--pyruvate aminotransferase
Operon_262	lilo_0406	LILO_RS02150	CTP synthase
Operon_263	lilo_0407	LILO_RS02155	L-2-hydroxyisocaproate/malate/lactate dehydrogenase
Operon_264	lilo_0408	LILO_RS02160	hypothetical protein
Operon_265	lilo_0409	LILO_RS02170	nucleoside 2-deoxyribosyltransferase
	lilo_0410	lilo_0410	Lysine decarboxylase family protein
	lilo_0411	LILO_RS02180	deoxyguanosine kinase
Operon_266	lilo_0412	LILO_RS02185	DNA polymerase III, alpha chain 2
Operon_267	lilo_0413	LILO_RS02190	hemolysin like protein
Operon_268	lilo_0414	LILO_RS02195	DegV family fatty acid-binding protein
	lilo_0415	LILO_RS02200	lipase/acylhydrolase
	lilo_0416	LILO_RS02205	beta-N-acetylhexosaminidase
	lilo_0417	LILO_RS02210	hypothetical protein
Operon_269	lilo_0418	LILO_RS02215	HU-like DNA-binding protein
Operon_270	lilo_0419	LILO_RS02220	transcription regulator PaiA
Operon_271	lilo_0420	LILO_RS02225	6-O-methylguanine-DNA methyltransferase
	lilo_0421	LILO_RS02230	methylphosphotriester-DNA alkyltransferase
Operon_272	lilo_0422	LILO_RS02235	ABC transporter ATP binding protein
	lilo_0423	LILO_RS02240	ABC transporter permease protein
	lilo_0424	LILO_RS02245	LytTr DNA-binding-domain containing response regulator
	lilo_0425	LILO_RS02250	hypothetical protein
Operon_273	lilo_0426	LILO_RS02255	pyridoxal-phosphate dependent aminotransferase
Operon_274	lilo_0427	LILO_RS02260	gamma-glutamyl-diamino acid-endopeptidase
Operon_275	lilo_0428	LILO_RS02265	peptide-binding protein
Operon_276	lilo_0429	LILO_RS02270	cysteine synthase
Operon_277	lilo_0430	LILO_RS02275	N-acetylmuramidase
Operon_278	lilo_0431	LILO_RS02280	hypothetical protein
	lilo_0432	LILO_RS02285	penicillin-binding protein 1A
	lilo_0433	LILO_RS02290	putative recombination protein U
Operon_279	lilo_0434	LILO_RS02295	hypothetical protein
Operon_280	lilo_0435	LILO_RS02300	arsenate reductase family protein
Operon_281	lilo_0436	LILO_RS02305	hypothetical protein
	lilo_0437	LILO_RS02310	myo-inositol-1(or 4)-monophosphatase
Operon_282	lilo_0438	LILO_RS02315	UDP-N-acetylglucosamine 1-carboxyvinyltransferase
	lilo_0439	LILO_RS02320	hypothetical protein
Operon_283	lilo_0440	LILO_RS02325	trigger factor
Operon_284	lilo_0441	LILO_RS02420	DNA primase
	lilo_0442	lilo_0442	major RNA polymerase sigma factor
Operon_285	lilo_0443	lilo_0443	RpiR family transcriptional regulator
Operon_286	lilo_0444	LILO_RS02435	glycerol-3-phosphatase transporter
Operon_287	lilo_0445	LILO_RS02440	hypothetical protein

	lilo_0446	LILO_RS02445	ATP-dependent protease ATP-binding subunit
Operon_288	lilo_0447	LILO_RS02450	Rrf2 family transcriptional regulator
Operon_289	lilo_0448	LILO_RS02455	glyceraldehyde 3-phosphate dehydrogenase
Operon_290	lilo_0449	LILO_RS02460	polypeptide deformylase
Operon_291	lilo_0450	LILO_RS02465	diadenosine 5',5'''-P1,P4-tetraphosphate hydrolase
	lilo_0451	LILO_RS02470	excinuclease ABC subunit B
Operon_292	lilo_0452	LILO_RS02475	glutamate or arginine ABC transporter substrate binding protein
	lilo_0453	LILO_RS02480	acetylornithine deacetylase
Operon_293	lilo_0454	LILO_RS02485	hydroxymyristoyl-acyl carrier protein dehydratase
Operon_294	lilo_0455	LILO_RS02490	enoyl-[acyl-carrier protein] reductase (NADH)
Operon_295	lilo_0456	LILO_RS02500	OxaA/YidC family membrane protein
Operon_296	lilo_0457	LILO_RS02505	HAD superfamily hydrolase
Operon_297	lilo_0458	LILO_RS02510	hypothetical membrane protein
	lilo_0459	LILO_RS02515	phosphopantothenoylcysteine synthase/decarboxylase
	lilo_0460	LILO_RS02520	phosphopantothenate--cysteine ligase
Operon_298	lilo_0461	LILO_RS02525	4-oxalocrotonate tautomerase
Operon_299	lilo_0462	LILO_RS02530	cation-transporting ATPase
	lilo_0463	LILO_RS02535	putative acetyltransferase
Operon_300	lilo_0464	LILO_RS02540	hypothetical protein
Operon_301	lilo_0465	LILO_RS02545	hypothetical protein
Operon_302	lilo_0466	LILO_RS02550	carotenoid biosynthetic protein CrtK
Operon_303	lilo_0467	LILO_RS02555	hypothetical protein
	lilo_0468	LILO_RS02560	dihydroxyacetone kinase family protein
	lilo_0469	LILO_RS02565	hypothetical protein
Operon_304	lilo_0470	LILO_RS02570	glyoxalase family protein
Operon_305	lilo_0471	LILO_RS02575	hypothetical protein
Operon_306	lilo_0472	LILO_RS02580	hypothetical protein
Operon_307	lilo_0473	LILO_RS02585	hypothetical protein
	lilo_0474	LILO_RS02590	SOS responce UmuC protein
Operon_308	lilo_0475	LILO_RS02595	GNAT family acetyltransferase
Operon_309	lilo_0476	LILO_RS02600	phage protein
Operon_310	lilo_0477	LILO_RS02605	MarR family transcriptional regulator
Operon_311	lilo_0478	LILO_RS02610	hypothetical protein
Operon_312	lilo_0479	LILO_RS02615	thymidine kinase
	lilo_0480	LILO_RS02620	hypothetical protein
	lilo_0481	LILO_RS02625	peptide chain release factor RF-1
	lilo_0482	LILO_RS02630	hypothetical protein
	lilo_0483	LILO_RS02635	NADPH-flavin oxidoreductase
Operon_313	lilo_0484	LILO_RS02640	protoporphyrinogen oxidase
	lilo_0485	LILO_RS02645	GNAT family acetyltransferase
	lilo_0486	lilo_0486	Sua5/YciO/YrdC/YwlC family RNA-binding protein
Operon_314	lilo_0487	LILO_RS02655	serine hydroxymethyltransferase
Operon_315	lilo_0488	LILO_RS02660	transposon-related protein
Operon_316	lilo_0489	LILO_RS02665	phosphoserine aminotransferase
	lilo_0490	LILO_RS02670	D-3-phosphoglycerate dehydrogenase
	lilo_0491	LILO_RS02675	phosphoserine phosphatase

Operon_317	lilo_0492	LILO_RS02680	acylphosphate phosphohydrolase
Operon_318	lilo_0493	LILO_RS02685	tRNA/rRNA methyltransferase
Operon_319	lilo_0494	LILO_RS02695	flavodoxin
Operon_320	lilo_0495	LILO_RS02700	membrane-bound transport protein
Operon_321	lilo_0496	LILO_RS02705	putative transcriptional regulator, TetR family
Operon_322	lilo_0497	LILO_RS02710	mathionine aminopeptidase
	lilo_0498	LILO_RS02715	putative tRNA-processing ribonuclease BN
Operon_323	lilo_0499	LILO_RS02720	hypothetical protein
Operon_324	lilo_0500	LILO_RS02725	hypothetical protein
	lilo_0501	LILO_RS02730	hypothetical protein
Operon_325	lilo_0502	LILO_RS02735	hypothetical protein
Operon_326	lilo_0503	LILO_RS02740	hpr(ser) kinase / phosphatase
	lilo_0504	LILO_RS02745	prolipoprotein diacylglycerol transferase
Operon_327	lilo_0505	LILO_RS02750	hypothetical protein
	lilo_0506	LILO_RS02755	hypothetical protein
Operon_328	lilo_0507	LILO_RS02760	6-phosphogluconate dehydrogenase, decarboxylating
Operon_329	lilo_0508	LILO_RS02765	potassium uptake protein
Operon_330	lilo_0509	LILO_RS02770	potassium uptake protein
Operon_331	lilo_0510	lilo_0510	hypothetical protein
Operon_332	lilo_0511	LILO_RS02775	hypothetical protein
Operon_333	lilo_0512	LILO_RS02780	tRNA isopentenyltransferase
Operon_334	lilo_0513	LILO_RS02785	hypothetical protein
Operon_335	lilo_0514	LILO_RS02790	hypothetical protein
Operon_336	lilo_0515	LILO_RS02800	hypothetical protein
Operon_337	lilo_0516	LILO_RS02810	ribonuclease Z
	lilo_0517	LILO_RS02815	oxidoreductase
Operon_338	lilo_0518	LILO_RS02820	single-stranded DNA specific exonuclease
	lilo_0519	LILO_RS02825	adenine phosphoribosyltransferase
Operon_339	lilo_0520	LILO_RS02830	DNA-directed RNA polymerase delta chain
Operon_340	lilo_0521	LILO_RS02835	aminodeoxychorismate lyase
	lilo_0522	LILO_RS02840	transcription elongation factor GreA
Operon_341	lilo_0523	LILO_RS02845	class III stress genes transcriptional repressor CstR
	lilo_0524	LILO_RS02850	ATP-dependent protease ATP-binding subunit
Operon_342	lilo_0525	LILO_RS02855	ribosome-associated factor Y
Operon_343	lilo_0526	LILO_RS02860	enolase
Operon_344	lilo_0527	LILO_RS02865	ABC transporter ATP-binding protein
	lilo_0528	LILO_RS02870	ABC transporter permease protein
	lilo_0529	LILO_RS02875	TetR family transcriptional regulator
Operon_345	lilo_0530	LILO_RS02880	long-chain acyl-CoA synthetase
Operon_346	lilo_0531	LILO_RS02885	divalent cation transport-related protein
Operon_347	lilo_0532	LILO_RS02890	pyruvate-formate lyase
Operon_348	lilo_0533	LILO_RS02895	dephospho-CoA kinase
	lilo_0534	LILO_RS02900	multidrug resistance efflux pump
	lilo_0535	LILO_RS02905	50S ribosomal protein L33
Operon_349	lilo_0536	LILO_RS02910	cell division protein FtsW
	lilo_0537	LILO_RS02915	pyruvate carboxylase

Operon_350	lilo_0538	LILO_RS02920	citrate synthase
	lilo_0539	LILO_RS02925	aconitate hydratase
	lilo_0540	LILO_RS02930	isocitrate dehydrogenase
Operon_351	lilo_0541	LILO_RS02935	ATP-dependent Clp protease proteolytic subunit
Operon_352	lilo_0542	LILO_RS02940	hypothetical protein
Operon_353	lilo_0543	LILO_RS02945	arsenate reductase family protein
Operon_354	lilo_0544	lilo_0544	putative secreted protein
Operon_355	lilo_0545	LILO_RS02950	ABC transporter permease
Operon_356	lilo_0546	LILO_RS02955	calcium-transporting ATPase
Operon_357	lilo_0547	LILO_RS02960	hypothetical protein
	lilo_0548	LILO_RS02965	similar to intercellular adhesion protein A
Operon_358	lilo_0549	LILO_RS02975	intercellular adhesion protein IcaB
	lilo_0550	LILO_RS02980	collagen adhesin
Operon_359	lilo_0552	LILO_RS02995	branched-chain amino acid transport system carrier protein
Operon_360	lilo_0553	LILO_RS03000	GNAT family acetyltransferase
	lilo_0554	LILO_RS03005	transposase of IS904I
Operon_361	lilo_0555	LILO_RS03010	transposase
Operon_362	lilo_0556	LILO_RS03015	GTNG_0265 lantibiotic antimicrobial precursor peptinisin Ade
	lilo_0557	LILO_RS03020	could encode enzyme catalyzing modification reactions
	lilo_0558	LILO_RS03025	nisin transport protein
	lilo_0559	LILO_RS03030	could encode enxyme catalyzing modification reactions
Operon_363	lilo_0560	lilo_0560	anthionine synthetase N-terminal
Operon_364	lilo_0561	LILO_RS03035	encodes a protein involved in immunity against nisin
	lilo_0562	LILO_RS03040	cleave leader peptide/cell wall-associated serine proteasee proteinase
Operon_365	lilo_0563	LILO_RS03045	cytoplasmatic regulatory protein
	lilo_0564	LILO_RS03050	two component system histidine kinase
Operon_366	lilo_0565	LILO_RS03055	ABC transporter ATPase component
	lilo_0566	LILO_RS03060	major facilitator superfamily permease
	lilo_0567	LILO_RS03065	two component system histidine kinase
Operon_367	lilo_0568	LILO_RS03070	transposase ISXB
	lilo_0569	LILO_RS03075	transposase ISXA
	lilo_0570	LILO_RS03080	transposase
	lilo_0827	lilo_0827	transposase
Operon_368	lilo_0571	LILO_RS03085	LacI family sucrose operon repressor
	lilo_0572	LILO_RS03090	sucrase-6-phosphate hydrolase (sucrase, invertase)
Operon_369	lilo_0573	LILO_RS03095	sucrose-specific phosphotransferase system, enzymeIIBCA
	lilo_0574	LILO_RS03100	fructokinase
Operon_370	lilo_0575	lilo_0575	polysaccharide transporter
Operon_371	lilo_0576	LILO_RS03110	hypothetical protein
Operon_372	lilo_0577	LILO_RS03115	cold shock protein cspC
	lilo_0578	LILO_RS03120	cold shock protein CspD
	lilo_0579	LILO_RS03125	hypothetical protein
Operon_373	lilo_0581	LILO_RS03135	hypothetical protein

	lilo_0582	LILO_RS03140	hypothetical protein
	lilo_0583	lilo_0583	hypothetical protein
	lilo_1179	LILO_RS06135	hypothetical protein
Operon_374	lilo_0585	LILO_RS03155	putative DNA-binding protein, XRE family
Operon_375	lilo_0586	LILO_RS03160	transcription regulator
Operon_376	lilo_0587	LILO_RS03165	UDP-N-acetylglucosamine 2-epimerase
	lilo_0588	LILO_RS03170	hypothetical protein
Operon_377	lilo_0589	LILO_RS03175	glycosyl transferase, family 2
	lilo_0590	LILO_RS03180	putative beta-glucanase precursor
Operon_378	lilo_0591	lilo_0591	hypothetical protein
	lilo_0592	lilo_0592	hypothetical protein
	lilo_0593	LILO_RS03195	hypothetical protein
Operon_379	lilo_0594	LILO_RS03200	ABC transporter permease protein
	lilo_0595	LILO_RS03205	ABC transporter ATP-binding protein
Operon_380	lilo_0596	LILO_RS03215	hypothetical protein
	lilo_0597	LILO_RS03220	Cro/CI family transcriptional regulator
Operon_381	lilo_0598	lilo_0598	membrane-associated serine protease
Operon_382	lilo_0599	LILO_RS03230	Cro/CI family transcriptional regulator
	lilo_0600	LILO_RS03235	hypothetical protein
	lilo_0601	LILO_RS03240	ribosomal protein L5
	lilo_0602	LILO_RS03245	putative nucleolar protein
	lilo_0603	LILO_RS03250	putative transposon protein
Operon_383	lilo_0604	lilo_0604	cysteine desulfurase
Operon_384	lilo_0605	LILO_RS03260	Cro/CI family transcriptional regulator
	lilo_0606	LILO_RS03265	ATP-dependent deoxyribonuclease subunit B
	lilo_0607	LILO_RS03270	hypothetical protein
	lilo_0608	LILO_RS03275	histidinol-phosphate/aromatic aminotransferase and cobyric acid decarboxylase
	lilo_0611	LILO_RS03295	hypothetical cell surface protein precursor
	lilo_0612	LILO_RS03305	translation initiation factor 2
	lilo_0613	LILO_RS03310	hypothetical protein
	lilo_0614	LILO_RS03320	putative conjugative transposon protein
Operon_385	lilo_0609	LILO_RS03285	hypothetical protein
	lilo_0610	LILO_RS03290	ABC transporter ATP-binding protein
Operon_386	lilo_0615	LILO_RS03325	putative metal-dependent phosphohydrolase precursor
	lilo_0616	lilo_0616	cell wall binding domain protein
Operon_387	lilo_0617	LILO_RS03335	hypothetical protein
	lilo_0618	LILO_RS03340	Antirestriction protein
Operon_388	lilo_0619	LILO_RS03345	putative Tn5276 excisionase
	lilo_0620	LILO_RS03350	IntTn5276 protein
Operon_389	lilo_0621	LILO_RS03355	TatD family Dnase
Operon_390	lilo_0622	LILO_RS03360	DNA topology modulation protein
	lilo_0623	LILO_RS03365	ribonuclease M5
Operon_391	lilo_0624	LILO_RS03370	dimethyladenosine transferase
	lilo_0625	LILO_RS03375	Xaa-Pro-(Xaa)n proline peptidase
Operon_392	lilo_0626	LILO_RS03380	elongation factor EF-P
Operon_393	lilo_0627	LILO_RS03385	Gls24 family general stress protein

	lilo_0628	LILO_RS03390	transcription termination protein NusB
Operon_394	lilo_0629	LILO_RS03395	4-alpha-glucanotransferase
	lilo_0630	LILO_RS03400	glucose-1-phosphate adenylyltransferase
	lilo_0631	LILO_RS03405	glucose-1-phosphate adenylyltransferase
	lilo_0632	LILO_RS03410	glycogen synthase
	lilo_0633	LILO_RS03415	glycogen phosphorylase
	lilo_0634	LILO_RS03420	amylopullulanase/ glucan 1,4-alpha-maltohydrolase
Operon_395	lilo_0635	LILO_RS03425	di-/tripeptide transporter
Operon_396	lilo_0636	lilo_0636	hypothetical protein
Operon_397	lilo_0637	LILO_RS03430	cytochrome bd-I oxidase subunit I
	lilo_0638	LILO_RS03435	cytochrome d ubiquinol oxidase subunit II
Operon_398	lilo_0639	LILO_RS03440	cytochrome D ABC transporter ATP binding and permease protein
	lilo_0640	LILO_RS03445	cytochrome D ABC transporter ATP binding and permease protein
Operon_399	lilo_0641	lilo_0641	hypothetical protein
	lilo_0642	LILO_RS03450	MarR family transcriptional regulator
	lilo_0643	LILO_RS03455	putative flavodoxin
	lilo_0644	LILO_RS03460	GDSL-like lipase/acylhydrolase family protein
Operon_400	lilo_0645	LILO_RS03465	multidrug resistance ABC transporter ATP binding and permease protein
Operon_401	lilo_0646	LILO_RS03470	predicted integral membrane protein
Operon_402	lilo_0647	LILO_RS03475	alkaline phosphatase
Operon_403	lilo_0648	LILO_RS03480	ABC transporter ATP binding and permease protein
Operon_404	lilo_0649	LILO_RS03485	Zn-ribbon nucleic-acid-binding protein
Operon_405	lilo_0650	LILO_RS03490	hypothetical protein
Operon_406	lilo_0651	LILO_RS03495	quinone oxidoreductase
Operon_407	lilo_0652	LILO_RS03500	5-methyltetrahydropteroyltriglutamate--homocysteinemethyltransferase
Operon_408	lilo_0653	LILO_RS03505	putative metal-dependent hydrolase
	lilo_0654	LILO_RS03510	hypothetical protein
	lilo_0655	LILO_RS03515	tRNA-specific adenosine deaminase
	lilo_0656	LILO_RS03520	hypothetical protein
Operon_409	lilo_0657	LILO_RS03525	hypothetical protein
Operon_410	lilo_0658	lilo_0658	1,4-dihydroxy-2-naphthoyl-CoA thioesterase
	lilo_0659	LILO_RS03530	O-succinylbenzoate-CoA synthase
	lilo_0660	LILO_RS03535	O-succinylbenzoic acid--CoA ligase
Operon_411	lilo_0661	LILO_RS03540	naphthoate synthase
Operon_412	lilo_0662	LILO_RS03545	YtxM-like protein/ menaquinone biosynthesis related protein
	lilo_0663	LILO_RS03550	2-oxoglutarate decarboxylase / 2-succinyl-6-hydroxy-2,4-cyclohexadiene-1-carboxylate synthase
	lilo_0664	LILO_RS03555	menaquinone-specific isochorismate synthase
Operon_413	lilo_0665	LILO_RS03560	ribosomal-protein-alanine acetyltransferase
	lilo_0666	LILO_RS03565	cBS domain containing protein
	lilo_0667	LILO_RS03570	phosphoesterase, DHH family protein
Operon_414	lilo_0668	LILO_RS03575	L-asparaginase
Operon_415	lilo_0669	LILO_RS03580	L-asparaginase
Operon_416	lilo_0670	LILO_RS03585	hypothetical protein

	lilo_0671	LILO_RS03590	flotillin-like protein
Operon_417	lilo_0672	LILO_RS03595	aspartate kinase
Operon_418	lilo_0673	LILO_RS03600	predicted sugar phosphatase of HAD family
Operon_419	lilo_0674	LILO_RS03605	signaling protein
	lilo_0675	LILO_RS03610	50S ribosomal protein L9
	lilo_0676	LILO_RS03615	replicative DNA helicase
Operon_420	lilo_0677	LILO_RS03620	hypothetical protein
Operon_421	lilo_0678	LILO_RS03625	putative kinase
Operon_422	lilo_0679	LILO_RS03630	tRNA (guanine-N7-)-methyltransferase
Operon_423	lilo_0680	LILO_RS03635	ATP-cone domain protein
Operon_424	lilo_0681	LILO_RS03640	replication protein DnaB
	lilo_0682	LILO_RS03645	primosomal protein DnaI
	lilo_0683	LILO_RS03650	oxidoreductase
	lilo_0684	LILO_RS03655	uncharacterized low-complexity protein
Operon_425	lilo_0685	LILO_RS03660	GTP-binding protein
Operon_426	lilo_0686	LILO_RS03665	Rgg/GadR/MutR family transcriptional regulator
Operon_427	lilo_0687	LILO_RS03670	cell surface protein
Operon_428	lilo_0688	LILO_RS03685	hypothetical protein
Operon_429	lilo_0689	LILO_RS03690	hypothetical protein
	lilo_0690	LILO_RS03695	sortase, putative
	lilo_0691	LILO_RS03700	hypothetical protein
Operon_430	lilo_0692	LILO_RS03705	murein hydrolase export regulator
	lilo_0693	LILO_RS03710	murein hydrolase exporter
Operon_431	lilo_0694	LILO_RS03715	hypothetical protein
Operon_432	lilo_0695	LILO_RS03720	transcription termination protein NusA
Operon_433	lilo_0696	LILO_RS03725	hypothetical protein
	lilo_0697	LILO_RS03730	50S ribosomal protein L7AE
	lilo_0698	LILO_RS03735	translation initiation factor IF-2
Operon_434	lilo_0699	LILO_RS03740	ribosome-binding factor A
Operon_435	lilo_0700	LILO_RS03745	mannose-6-phosphate isomerase
Operon_436	lilo_0701	LILO_RS03750	MarR family transcriptional regulator
	lilo_0702	LILO_RS03755	3-oxoacyl-[acyl-carrier-protein] synthase III
Operon_437	lilo_0703	LILO_RS03760	acyl carrier protein
Operon_438	lilo_0704	LILO_RS03765	malonyl CoA-acyl carrier protein transacylase
	lilo_0705	LILO_RS03770	3-oxoacyl-[acyl-carrier protein] reductase
	lilo_0706	LILO_RS03775	3-oxoacyl-[acyl-carrier-protein] synthase II
	lilo_0707	LILO_RS03780	acetyl-CoA carboxylase biotin carboxyl carrier protein
Operon_439	lilo_0708	LILO_RS03785	(3R)-hydroxymyristoyl-(acyl carrier protein) dehydratase
Operon_440	lilo_0709	LILO_RS03790	acetyl-CoA carboxylase biotin carboxylase
	lilo_0710	LILO_RS03795	acetyl-CoA carboxylase carboxyl transferase subunit beta
	lilo_0711	LILO_RS03800	acetyl-CoA carboxylase,carboxyl transferase subunit alpha
Operon_441	lilo_0712	LILO_RS03805	LacI family transcriptional regulator
Operon_442	lilo_0713	LILO_RS03810	hypothetical protein
	lilo_0714	LILO_RS03815	hypothetical protein

	lilo_0715	LILO_RS03820	hypothetical protein
	lilo_0716	LILO_RS03825	hypothetical protein
	lilo_0717	LILO_RS03830	hypothetical protein
Operon_443	lilo_0718	LILO_RS03835	cystathionine beta-lyase
Operon_444	lilo_0719	LILO_RS03840	cysteine synthase
Operon_445	lilo_0720	LILO_RS03845	hypothetical protein
Operon_446	lilo_0721	LILO_RS03850	putative rhodanese-related sulfurtransferase
	lilo_0722	LILO_RS03855	putative rhodanese-related sulfurtransferase
	lilo_0723	LILO_RS03860	NADH dehydrogenase
	lilo_0724	LILO_RS03865	hypothetical protein, nickel resistance determinant
Operon_447	lilo_0725	LILO_RS03870	arsenate reductase related protein, glutaredoxin family
Operon_448	lilo_0726	LILO_RS03875	DeoR family transcriptional regulator
Operon_449	lilo_0727	LILO_RS03880	acetyl transferase
Operon_450	lilo_0728	LILO_RS03885	exodeoxyribonuclease III
Operon_451	lilo_0729	LILO_RS03890	methionyl-tRNA synthetase
Operon_452	lilo_0730	lilo_0730	hypothetical protein
Operon_453	lilo_0731	LILO_RS03895	hypothetical protein
	lilo_0732	lilo_0732	3-oxoacyl-[acyl-carrier protein] reductase
	lilo_0733	LILO_RS03900	3-oxoacyl-[acyl-carrier protein] reductase
Operon_455	lilo_0734	LILO_RS03905	queuosine transporter
Operon_456	lilo_0735	LILO_RS03910	putative NADH-flavin reductase
Operon_457	lilo_0736	LILO_RS03915	N-acetyl-gamma-glutamyl-phosphate reductase
	lilo_0737	LILO_RS03920	glutamate N-acetyltransferase / amino-acid N-acetyltransferase
	lilo_0738	LILO_RS03925	acetylornithine aminotransferase
	lilo_0739	LILO_RS03930	acetylglutamate kinase
	lilo_0740	LILO_RS03935	ornithine carbamoyltransferase
Operon_458	lilo_0741	LILO_RS03940	ribonuclease III
	lilo_0742	LILO_RS03945	chromosome segregation protein smc
Operon_459	lilo_0743	LILO_RS03950	alkaline phosphatase superfamily protein
Operon_460	lilo_0744	LILO_RS03955	hypothetical protein
	lilo_0745	LILO_RS03960	YibE/F superfamily transporter
	lilo_0746	LILO_RS03965	YibE/F superfamily transporter
Operon_461	lilo_0747	LILO_RS03970	GABA-specific permease
Operon_462	lilo_0748	LILO_RS03975	predicted hydrolase of the HAD superfamily
	lilo_0749	LILO_RS03980	hypothetical protein
Operon_463	lilo_0750	LILO_RS03985	cell division protein FtsY
Operon_464	lilo_0751	LILO_RS03990	ribose-phosphate pyrophosphokinase
Operon_465	lilo_0752	LILO_RS03995	putative regulator
Operon_466	lilo_0753	LILO_RS04000	leucyl-tRNA synthetase
Operon_467	lilo_0754	LILO_RS04005	HTH-type transcriptional regulator
Operon_468	lilo_0755	LILO_RS04010	cellobiose-specific PTS system IIC component
Operon_469	lilo_0756	LILO_RS04015	beta-glucosidase
Operon_470	lilo_0757	LILO_RS04020	non-heme chloride peroxidase
Operon_471	lilo_0758	LILO_RS04025	hypothetical protein
Operon_472	lilo_0759	LILO_RS04030	NADH dehydrogenase

Operon_473	lilo_0760	LILO_RS04035	NADH dehydrogenase
Operon_474	lilo_0761	LILO_RS04040	L-serine dehydratase beta subunit
	lilo_0762	LILO_RS04045	L-serine dehydratase alpha subunit
Operon_475	lilo_0763	LILO_RS04050	copper transport repressor
	lilo_0764	LILO_RS04055	copper chaperone CopZ
	lilo_0765	LILO_RS04060	copper-translocating P-type ATPase
Operon_477	lilo_0766	LILO_RS04065	ABC transporter permease
Operon_478	lilo_0767	LILO_RS04070	putative galactose-1-phosphate uridylyltransferase
Operon_479	lilo_0768	LILO_RS04075	tRNA (5-methylaminomethyl-2-thiouridylate)-methyltransferase
Operon_480	lilo_0769	LILO_RS04080	30S ribosomal protein S1
	lilo_0770	LILO_RS04085	hypothetical protein
	lilo_0771	LILO_RS04090	DNA-binding response regulator, OmpR family
Operon_481	lilo_0772	LILO_RS04095	signal transduction histidine kinase
Operon_482	lilo_0773	LILO_RS04100	uridine phosphorylase
Operon_483	lilo_0774	LILO_RS04105	nicotinamide mononucleotide transporter
Operon_484	lilo_0775	LILO_RS04110	excinuclease ABC subunit C
Operon_485	lilo_0776	LILO_RS04115	A/G-specific adenine glycosylase
Operon_486	lilo_0777	LILO_RS04120	dipeptidase
Operon_487	lilo_0778	LILO_RS04125	holo-[acyl-carrier protein] synthase
	lilo_0779	LILO_RS04130	alanine racemase
Operon_488	lilo_0780	LILO_RS04135	arsenate reductase family protein
Operon_489	lilo_0781	LILO_RS04140	glutathione reductase
Operon_490	lilo_0782	LILO_RS04145	choline ABC transporter ATP binding protein
	lilo_0783	LILO_RS04150	choline ABC transporter permease and substrate binding protein
Operon_491	lilo_0784	LILO_RS04155	hypothetical protein
Operon_492	lilo_0785	LILO_RS04160	hypothetical protein
Operon_493	lilo_0786	LILO_RS04165	hypothetical protein
Operon_494	lilo_0787	LILO_RS04170	copper-potassium transporting ATPase B
	lilo_0788	LILO_RS04175	hypothetical protein
Operon_495	lilo_0789	LILO_RS04180	methylenetetrahydrofolate dehydrogenase (NADP+) / methenyltetrahydrofolate cyclohydrolase
Operon_496	lilo_0790	LILO_RS04185	exonuclease VII large subunit
	lilo_0791	LILO_RS04190	exonuclease VII small subunit
Operon_497	lilo_0792	LILO_RS04195	glycerate kinase
	lilo_0793	LILO_RS04200	geranyltranstransferase/ dimethylallyltransferase
	lilo_0794	LILO_RS04205	rRNA methylase
	lilo_0795	LILO_RS04210	transcriptional repressor, arginine deiminase pathway
Operon_498	lilo_0796	LILO_RS04215	DNA repair protein RecN
Operon_499	lilo_0797	LILO_RS04220	hypothetical protein
Operon_500	lilo_0798	LILO_RS04225	hypothetical protein
Operon_501	lilo_0799	LILO_RS04230	transporter
	lilo_0800	LILO_RS04235	hypothetical protein
Operon_502	lilo_0801	LILO_RS04240	S-adenosyl-methyltransferase
Operon_503	lilo_0802	LILO_RS04245	cell division protein FtsL
	lilo_0803	LILO_RS04250	penicillin-binding protein
	lilo_0804	LILO_RS04255	phospho-N-acetylmuramoyl-pentapeptide-transferase

Operon_504	lilo_0805	LILO_RS04260	hypothetical protein
Operon_506	lilo_0806	LILO_RS04265	ABC transporter permease protein
	lilo_0807	LILO_RS04270	ABC transporter ATP-binding protein
Operon_507	lilo_0808	LILO_RS04275	malolactic fermentation system transcriptional activator
Operon_508	lilo_0809	LILO_RS04280	GTP pyrophosphokinase, RelA/SpoT superfamily
Operon_509	lilo_0810	LILO_RS04285	50S ribosomal protein L19
Operon_510	lilo_0811	LILO_RS04290	glutamate synthase (NADPH) small chain
	lilo_0812	LILO_RS04295	glycosyl transferase
Operon_511	lilo_0813	LILO_RS04300	hypothetical protein
Operon_512	lilo_0814	LILO_RS04305	nicotinamide mononucleotide transporter
Operon_513	lilo_0815	lilo_0815	hypothetical protein
Operon_514	lilo_0816	LILO_RS04310	universal stress protein family
Operon_515	lilo_0817	LILO_RS04315	HU-like DNA-binding protein
Operon_516	lilo_0818	LILO_RS04320	transcription regulator
Operon_517	lilo_0819	LILO_RS04325	ABC transporter phage infection protein
Operon_518	lilo_0820	LILO_RS04330	hypothetical protein
Operon_519	lilo_0821	LILO_RS04335	phage integrase
Operon_520	lilo_0822	lilo_0822	putayive integrase/recombinase
Operon_521	lilo_0823	LILO_RS04345	hypothetical protein
Operon_522	lilo_0824	LILO_RS04350	hypothetical protein
	lilo_0825	lilo_0825	hypothetical protein
Operon_523	lilo_0826	LILO_RS04355	hypothetical protein
Operon_524	lilo_0828	LILO_RS04370	type I restriction enzyme R protein
	lilo_0829	LILO_RS04375	type I restriction enzyme M protein
	lilo_0830	LILO_RS04380	type I restriction enzyme, S subunit
Operon_525	lilo_0832	LILO_RS04395	hypothetical protein
Operon_526	lilo_0833	LILO_RS04400	hypothetical protein
Operon_527	lilo_0834	LILO_RS04405	hypothetical protein
Operon_528	lilo_0835	LILO_RS04410	cell division protein FtsW
	lilo_0836	LILO_RS04415	LytR family transcriptional regulator
Operon_529	lilo_0837	LILO_RS04420	30S ribosomal protein S14
Operon_530	lilo_0838	LILO_RS04425	hypothetical protein
	lilo_0839	LILO_RS04430	two-component sensor protein kinase
	lilo_0840	LILO_RS04435	two-component response regulator
	lilo_0841	LILO_RS04440	HAD superfamily hydrolase
Operon_531	lilo_0842	LILO_RS04445	peptidyl-prolyl cis-trans isomerase
	lilo_0843	LILO_RS04450	general stress protein GSP13
	lilo_0844	lilo_0844	transporter, drug/metabolite exporter family
	lilo_0845	LILO_RS04455	hypothetical protein
Operon_532	lilo_0846	LILO_RS04460	rod-shape determining protein
Operon_533	lilo_0847	LILO_RS04465	2,3-butanediol dehydrogenase
	lilo_0848	LILO_RS04470	acetoin reductase
Operon_534	lilo_0849	lilo_0849	hypothetical protein
Operon_535	lilo_0850	LILO_RS04475	ABC transporter ATP binding protein
Operon_536	lilo_0851	LILO_RS04480	malolactic enzyme
	lilo_0852	LILO_RS04485	malate/lactate antiporter

Operon_537	lilo_0853	LILO_RS04490	drug/metabolite exporter family transporter
Operon_538	lilo_0854	LILO_RS04495	hypothetical protein
	lilo_0855	LILO_RS04500	HAD superfamily hydrolase
Operon_539	lilo_0856	LILO_RS04505	DNA gyrase subunit B
Operon_540	lilo_0857	LILO_RS04510	hypothetical protein
Operon_541	lilo_0858	LILO_RS04515	SAM-dependent methyltransferase
Operon_542	lilo_0859	LILO_RS04520	hypothetical protein
Operon_543	lilo_0860	LILO_RS04525	MarR family transcriptional regulator
	lilo_0861	LILO_RS04530	multidrug resistance protein
	lilo_0862	LILO_RS04535	hypothetical protein
Operon_544	lilo_1018	LILO_RS05310	hypothetical protein
	lilo_0863	LILO_RS04540	N-acetylglucosamine-1-phosphodiester alpha-N-acetylglucosaminidase related protein
	lilo_0864	LILO_RS04545	teichoic acid biosynthesis protein
	lilo_0866	LILO_RS04555	teichoic acid ABC transporter ATP binding protein
	lilo_0867	LILO_RS04560	teichoic acid ABC transporter permease protein
Operon_545	lilo_0868	LILO_RS04565	lipopolysaccharide biosynthesis glycosyltransferase
Operon_546	lilo_0869	lilo_0869	prophage pi1 protein 12
Operon_547	lilo_0870	LILO_RS11725	putative acetyltransferase
	lilo_0871	lilo_0871	ATPase associated with chromosome architecture/replication
Operon_548	lilo_0872	LILO_RS04595	hypothetical protein
	lilo_0865	LILO_RS04550	hypothetical protein
	lilo_0873	lilo_0873	glycerol-3-phosphate cytidylyltransferase
	lilo_0874	lilo_0874	glycerol-3-phosphate cytidylyltransferase
	lilo_0875	LILO_RS04605	teichoic acid biosynthesis protein F
	lilo_0876	LILO_RS04610	teichoic acid biosynthesis protein B
	lilo_0877	LILO_RS04615	LytR family transcriptional regulator
Operon_549	lilo_0878	LILO_RS04620	phosphopentomutase
	lilo_0879	LILO_RS04625	hypothetical protein
Operon_550	lilo_0880	LILO_RS04630	purine-nucleoside phosphorylase
Operon_551	lilo_0881	LILO_RS04635	hypothetical protein
Operon_552	lilo_0882	LILO_RS04640	hypothetical protein
Operon_553	lilo_0883	LILO_RS04645	formyltetrahydrofolate synthetase
Operon_554	lilo_0884	LILO_RS04650	gamma-D-glutamyl-meso-diaminopimelate peptidase I, NlpC/P60 family
Operon_555	lilo_0885	LILO_RS04655	amino acid ABC transporter substrate binding protein
Operon_556	lilo_0886	LILO_RS04660	amino acid ABC transporter permease protein
	lilo_0887	LILO_RS04665	amino acid ABC transporter ATP binding protein
	lilo_0888	LILO_RS04670	hypothetical protein
Operon_557	lilo_0889	LILO_RS04675	thioredoxin reductase
Operon_558	lilo_0890	LILO_RS04680	protein-export protein SecG
Operon_559	lilo_0891	LILO_RS04685	ribonuclease
	lilo_0892	LILO_RS04690	isochorismatase family protein
Operon_560	lilo_0893	LILO_RS04695	endonuclease/exonuclease/phosphatase family protein
Operon_561	lilo_0894	LILO_RS04700	prenyltransferase, UbiA superfamily
Operon_562	lilo_0895	LILO_RS04705	ACT domain-containing protein
Operon_563	lilo_0896	LILO_RS04710	hypothetical protein

Operon_564	lilo_0897	LILO_RS04715	hypothetical protein
Operon_565	lilo_0898	LILO_RS04720	phosphoglycerate mutase
	lilo_0899	LILO_RS04725	D-alanyl-D-alanine carboxypeptidase
	lilo_0900	LILO_RS04730	hypothetical protein
Operon_566	lilo_0901	LILO_RS04735	heat-inducible transcription repressor HrcA
	lilo_0902	LILO_RS04740	stress responce protein GrpE
	lilo_0903	LILO_RS04745	DnaK protein
Operon_567	lilo_0904	LILO_RS04750	myosin-crossreactive antigen
	lilo_0905	LILO_RS04755	amino acid aminohydrolase
Operon_568	lilo_0906	LILO_RS04760	lactose transport regulator
	lilo_0907	LILO_RS04765	putative 1-phosphofructokinase
	lilo_0908	LILO_RS04770	fructose-specific PTS system enzyme IIBC component
Operon_1376	lilo_0909	LILO_RS04775	cardiolipin synthase
	lilo_0910	LILO_RS04780	P-loop ATPase family
	lilo_0911	LILO_RS04785	hypothetical protein
Operon_569	lilo_0912	LILO_RS04790	hypothetical protein
Operon_570	lilo_0913	LILO_RS04795	TetR family transcriptional regulator
Operon_571	lilo_0914	LILO_RS04800	ABC transporter ATP-binding protein
	lilo_0915	LILO_RS04805	ABC transporter permease protein
Operon_572	lilo_0916	LILO_RS04810	hypothetical protein
Operon_573	lilo_0917	LILO_RS04815	formate/nitrite transporter, FNT family
Operon_574	lilo_0918	LILO_RS04820	hypothetical protein
Operon_575	lilo_0919	LILO_RS04825	calcineurin-like phosphoesterase
Operon_576	lilo_0920	LILO_RS04830	peptide chain release factor RF-2
Operon_577	lilo_0921	LILO_RS04835	cell-division ATP-binding protein FtsE
	lilo_0922	LILO_RS04840	cell division protein
Operon_578	lilo_0923	LILO_RS04845	ribonucleoside-diphosphate reductase beta chain
	lilo_0924	LILO_RS04850	ribonucleoside-diphosphate reductase alpha chain
	lilo_0925	LILO_RS04855	ribonucleotide reductase
	lilo_0926	LILO_RS04860	glutaredoxin-like protein
Operon_581	lilo_0927	LILO_RS04865	hypothetical protein
Operon_582	lilo_0928	LILO_RS04870	topoisomerase IV subunit B
Operon_583	lilo_0929	LILO_RS04875	hypothetical protein
	lilo_0930	LILO_RS04880	DNA polymerase III, epsilon chain
	lilo_0931	LILO_RS04885	ElaA protein
Operon_584	lilo_0932	LILO_RS04890	topoisomerase IV subunit B
Operon_585	lilo_0933	LILO_RS04895	CorA family Mg2+/Co2+ transporter
Operon_586	lilo_0934	LILO_RS04900	diaminohydroxyphosphoribosylaminopyrimidine deaminase / 5-amino-6-(5-phosphoribosylamino)uracil reductase
	lilo_0935	LILO_RS04905	riboflavin synthase alpha chain
	lilo_0936	LILO_RS04910	GTP cyclohydrolase II / 3,4-dihydroxy-2-butanone 4-phosphate synthase
	lilo_0937	LILO_RS04915	riboflavin synthase beta chain
	lilo_1219	LILO_RS06365	hypothetical protein
Operon_587	lilo_0938	LILO_RS04920	lipoprotein signal peptidase
	lilo_0939	LILO_RS04925	pseudouridine synthase

Operon_588	lilo_0940	LILO_RS04930	hypothetical protein
Operon_589	lilo_0941	LILO_RS04935	hypothetical protein
	lilo_0942	LILO_RS04940	polyprenyl-phosphate glycosyltransferase
Operon_590	lilo_0943	LILO_RS04945	two-component system regulator
	lilo_0944	LILO_RS04950	hypothetical protein
	lilo_0945	LILO_RS04955	sensor protein kinase
	lilo_0946	LILO_RS04960	PAP2 family membrane-associated phospholipid phosphatase
Operon_591	lilo_0947	LILO_RS04965	glucosamine--fructose-6-phosphate aminotransferase
Operon_592	lilo_0948	LILO_RS04970	DNA repair protein RadC
Operon_593	lilo_0949	lilo_0949	endolysin
Operon_594	lilo_0950	lilo_0950	lyzozyme M1
	lilo_0951	lilo_0951	lyzozyme M1 (1,4-beta-N-acetylmuramidase)
Operon_595	lilo_0952	LILO_RS04985	hypothetical protein
Operon_596	lilo_0953	LILO_RS04990	branched-chain amino acid permease
Operon_597	lilo_0954	lilo_0954	prophage protein
Operon_598	lilo_0955	LILO_RS05000	prophage protein
	lilo_0956	LILO_RS05005	hypothetical protein
Operon_599	lilo_0957	lilo_0957	hypothetical protein
	lilo_0958	LILO_RS05010	hypothetical protein
	lilo_1680	LILO_RS08705	hypothetical protein
Operon_600	lilo_0959	lilo_0959	cytochrome bd ubiquinol oxidase, subunit I
	lilo_0960	LILO_RS05015	Na(+)/H(+) antiporter
Operon_601	lilo_0961	LILO_RS05020	redox-sensing transcriptional repressor REX
Operon_602	lilo_0962	LILO_RS05025	arsenate reductase family protein
Operon_603	lilo_0963	LILO_RS05030	ABC transporter ATP binding protein
Operon_604	lilo_0964	LILO_RS05035	hypothetical protein
	lilo_0965	LILO_RS05040	hypothetical protein
Operon_605	lilo_0967	LILO_RS05045	hypothetical protein
Operon_606	lilo_0968	LILO_RS05050	Rgg/GadR/MutR family transcriptional regulator
Operon_607	lilo_0969	LILO_RS05055	hypothetical protein
Operon_608	lilo_0970	LILO_RS05065	orotate phosphoribosyltransferase
	lilo_0971	LILO_RS05070	dihydroorotase
Operon_610	lilo_0972	LILO_RS05075	surface protein
Operon_611	lilo_0973	LILO_RS05080	DNA replication protein DnaD
	lilo_0974	LILO_RS05085	endonuclease III
	lilo_0975	LILO_RS05090	hypothetical protein
	lilo_0976	LILO_RS05095	NIF3 (NGG1p interacting factor 3) family protein
Operon_612	lilo_0977	LILO_RS05100	hypothetical protein
Operon_613	lilo_0978	LILO_RS05105	HAD superfamily hydrolase
	lilo_0979	LILO_RS05110	dGTP triphosphohydrolase
Operon_614	lilo_0980	LILO_RS05115	hypothetical protein
	lilo_0981	LILO_RS05120	ABC transporter permease
Operon_615	lilo_0982	LILO_RS05125	50S ribosomal protein L21
	lilo_0983	LILO_RS05130	hypothetical protein
	lilo_0984	LILO_RS05135	50S ribosomal protein L27
Operon_616	lilo_0985	LILO_RS05140	putative proton-dependent manganese transporter

			group C beta
Operon_617	lilo_0986	LILO_RS05145	phosphate starvation inducible protein
	lilo_0987	LILO_RS05150	MutT/nudix family phosphohydrolase
Operon_618	lilo_0988	LILO_RS05155	hypothetical protein
	lilo_0989	LILO_RS05160	diacylglycerol kinase
	lilo_0990	LILO_RS05165	hypothetical protein
Operon_619	lilo_0991	LILO_RS05170	competence protein ComFC
	lilo_0992	LILO_RS05175	competence protein ComFA
Operon_620	lilo_0993	LILO_RS05180	hypothetical protein
Operon_621	lilo_0994	LILO_RS05185	VanZ family protein
	lilo_0995	LILO_RS05190	hypothetical protein
Operon_622	lilo_0996	LILO_RS05195	5'-nucleotidase
Operon_623	lilo_0997	LILO_RS05200	glycyl-tRNA synthetase subunit alpha
	lilo_0998	LILO_RS05205	glycyl-tRNA synthetase beta chain
Operon_624	lilo_0999	LILO_RS05210	hypothetical protein
Operon_625	lilo_1000	LILO_RS05215	chloride channel protein
Operon_626	lilo_1001	LILO_RS05220	nicotinate-nucleotide adenylyltransferase
Operon_627	lilo_1002	LILO_RS05225	hypothetical protein
Operon_628	lilo_1003	LILO_RS05230	putative nicotinate phosphoribosyltransferase
	lilo_1004	LILO_RS05235	GNAT family acetyltransferase
	lilo_1005	LILO_RS05240	NAD-synthetase
Operon_629	lilo_1006	LILO_RS05245	ABC transporter ATP-binding protein
	lilo_1007	LILO_RS05250	ABC transporter permease protein
Operon_630	lilo_1008	LILO_RS05255	cobyric acid synthase
	lilo_1009	LILO_RS05260	hypothetical protein
Operon_631	lilo_1010	LILO_RS05265	alpha-acetolactate decarboxylase
Operon_632	lilo_1011	LILO_RS05270	GTP-binding protein LepA
	lilo_1012	LILO_RS05275	oxidoreductase
Operon_633	lilo_1013	LILO_RS05280	amino acid permease
Operon_634	lilo_1014	LILO_RS05285	DNA gyrase subunit A
	lilo_1015	LILO_RS05290	thiamine biosynthesis lipoprotein
Operon_635	lilo_1016	LILO_RS05295	similar to sortase
Operon_636	lilo_1017	lilo_1017	hypothetical protein
Operon_637	lilo_1019	LILO_RS05315	lysyl-tRNA synthetase
	lilo_1020	LILO_RS05320	hypothetical protein
Operon_638	lilo_1021	LILO_RS05325	hypothetical protein
Operon_639	lilo_1022	LILO_RS05330	ATP-dependent helicase PcrA
	lilo_1023	LILO_RS05335	mutator protein MutT
	lilo_1024	LILO_RS05340	DNA-3-methyladenine glycosidase I
Operon_640	lilo_1025	LILO_RS05345	PhnB protein
Operon_641	lilo_1026	LILO_RS05350	fumarate reductase flavoprotein subunit
Operon_642	lilo_1027	LILO_RS05355	hypothetical protein
Operon_643	lilo_1028	LILO_RS05360	tRNA pseudouridine synthase B
	lilo_1029	LILO_RS05365	riboflavin kinase / FMN adenylyltransferase
	lilo_1030	LILO_RS05370	L-lactate dehydrogenase
Operon_644	lilo_1031	LILO_RS05375	hypothetical protein

	lilo_1032	LILO_RS05380	N-acetylmuramic acid-6-phosphate etherase
	lilo_1033	LILO_RS05385	sucrose-specific PTS system IIBC component
	lilo_1034	LILO_RS05390	RpiR family transcriptional regulator
Operon_645	lilo_1035	LILO_RS05395	triosephosphate isomerase
Operon_646	lilo_1036	LILO_RS05400	penicillin acylase
	lilo_1037	LILO_RS05405	hypothetical protein
Operon_647	lilo_1038	LILO_RS05410	hypothetical protein
	lilo_1039	LILO_RS05415	hypothetical protein
Operon_648	lilo_1040	LILO_RS05420	oxygen-independent coproporphyrogen III oxidase
Operon_649	lilo_1041	LILO_RS05425	putative autolytic lysozyme
Operon_650	lilo_1042	LILO_RS05430	dCMP deaminase
Operon_651	lilo_1043	LILO_RS05435	acyl-ACP thioesterase
	lilo_1044	LILO_RS05440	N-acetylglucosamine catabolic protein
Operon_652	lilo_1045	LILO_RS05445	hypothetical protein
Operon_653	lilo_1046	LILO_RS05450	GMP reductase
Operon_654	lilo_1047	LILO_RS05455	xanthine phosphoribosyltransferase
	lilo_1048	LILO_RS05460	xanthine permease
Operon_655	lilo_1049	LILO_RS05465	hypothetical protein
Operon_656	lilo_1050	LILO_RS05470	putative RNA methylase
Operon_657	lilo_1051	LILO_RS05475	dihydropholate reductase
	lilo_1052	LILO_RS05480	hypothetical protein
Operon_658	lilo_1053	LILO_RS05485	ATP dependent Clp protease
	lilo_1054	LILO_RS05490	GTP-binding protein
	lilo_1055	LILO_RS05495	dihydroneopterin aldolase
Operon_659	lilo_1056	LILO_RS05500	GTP cyclohydrolase I / 2-amino-4-hydroxy-6-hydroxymethyldihydropteridine pyrophosphokinase
	lilo_1057	LILO_RS05505	dihydropteroate synthase
	lilo_1058	LILO_RS05510	hypothetical protein
	lilo_1059	LILO_RS05515	folylpolyglutamate synthase
Operon_660	lilo_1060	LILO_RS05520	deoxynucleoside kinase
Operon_661	lilo_1061	LILO_RS05525	homoserine dehydrogenase
	lilo_1062	LILO_RS05530	homoserine kinase
Operon_662	lilo_1063	LILO_RS05535	hypothetical protein
	lilo_1064	LILO_RS05540	UDP-N-acetylenolpyruvoylglucosamine reductase
Operon_663	lilo_1065	LILO_RS05545	spermidine/putrescine ABC transporter ATP-binding protein
	lilo_1066	LILO_RS05550	spermidine/putrescine ABC transporter permease protein
	lilo_1067	LILO_RS05555	spermidine/putrescine ABC transporter permease protein
	lilo_1068	LILO_RS05560	spermidine/putrescine ABC transporter substrate binding protein
Operon_664	lilo_1069	LILO_RS05565	ATP-dependent exonuclease subunit A
Operon_665	lilo_1070	LILO_RS05575	exonuclease
Operon_666	lilo_1071	LILO_RS05585	positive transcription regulator
Operon_667	lilo_1072	LILO_RS05590	hypothetical protein
Operon_668	lilo_1073	LILO_RS05595	hypothetical protein
	lilo_1074	LILO_RS05600	hypothetical protein

	lilo_1075	LILO_RS05605	hypothetical protein
Operon_669	lilo_1076	LILO_RS05610	hypothetical protein
Operon_670	lilo_1077	LILO_RS05615	cardiolipin synthase
Operon_671	lilo_1078	LILO_RS05620	cation-transporting ATPase
Operon_672	lilo_1079	LILO_RS05625	putative N-acylglucosamine-6-phosphate 2-epimerase
Operon_673	lilo_1080	LILO_RS05630	hypothetical protein
Operon_674	lilo_1081	LILO_RS05635	putative RNA methyltransferase
	lilo_1082	LILO_RS05640	NAD(FAD)-utilizing dehydrogenase
	lilo_1083	LILO_RS05645	SAM-dependent methyltransferase
	lilo_1084	LILO_RS05650	predicted permease
	lilo_1085	LILO_RS05655	hypothetical protein
Operon_675	lilo_1086	LILO_RS05660	acetolactate synthase large subunit
Operon_677	lilo_1087	LILO_RS05665	histidine kinase
	lilo_1088	LILO_RS05670	two-component sensor kinase YycG
Operon_678	lilo_1089	LILO_RS05680	similar to potassium-transporting atpaseA chain
	lilo_1090	LILO_RS05685	putative copper-transporting P-type ATPase
Operon_679	lilo_1091	LILO_RS05695	transcriptional regulator, xre family
Operon_680	lilo_1092	LILO_RS05700	hypothetical protein
Operon_681	lilo_1093	LILO_RS05705	ribonuclease
Operon_682	lilo_1094	LILO_RS05710	histidinol-phosphate aminotransferase
	lilo_1095	LILO_RS05715	ATP phosphoribosyltransferase regulatory subunit
	lilo_1096	LILO_RS05720	ATP phosphoribosyltransferase
	lilo_1097	LILO_RS05725	histidinol dehydrogenase
	lilo_1098	LILO_RS05730	SAM-dependent methyltransferase
	lilo_1099	LILO_RS05735	imidazoleglycerol-phosphate dehydratase
	lilo_1100	LILO_RS05740	kanamycin kinase
	lilo_1101	LILO_RS05745	amidotransferase
	lilo_1102	LILO_RS05750	phosphoribosylformimino-5-aminoimidazole carboxamideribotide isomerase
	lilo_1103	LILO_RS05755	imidazoleglycerol-phosphate synthase cyclase
	lilo_1104	LILO_RS05760	phosphoribosyl-AMP cyclohydrolase / phosphoribosyl-ATP pyrophosphohydrolase
	lilo_1105	LILO_RS05765	histidinol phosphatase
Operon_683	lilo_1106	LILO_RS05770	hypothetical protein
Operon_684	lilo_1107	LILO_RS05775	isopropylmalate synthase
	lilo_1108	LILO_RS05780	3-isopropylmalate dehydrogenase
Operon_685	lilo_1109	LILO_RS05785	3-isopropylmalate dehydratase large subunit
	lilo_1110	LILO_RS05790	3-isopropylmalate dehydratase small subunit
	lilo_1111	LILO_RS05795	ABC transporter ATP binding protein
Operon_686	lilo_1112	LILO_RS05800	dihydroxy-acid dehydratase
	lilo_1113	LILO_RS05805	acetolactate synthase large subunit
	lilo_1114	LILO_RS05810	acetolactate synthase small subunit
	lilo_1115	LILO_RS05815	ketol-acid reductoisomerase
	lilo_1116	LILO_RS05820	threonine deaminase
Operon_687	lilo_1117	LILO_RS05825	alpha-acetolactate decarboxylase
	lilo_1118	LILO_RS05830	regulatory protein AldR
Operon_688	lilo_1119	LILO_RS05835	smf protein

Operon_689	lilo_1120	LILO_RS05840	DNA topoisomerase I
	lilo_1121	LILO_RS05845	glucose inhibited division protein
Operon_690	lilo_1122	LILO_RS05850	tyrosine recombinase
Operon_691	lilo_1123	LILO_RS05855	hypothetical protein
Operon_692	lilo_1124	LILO_RS05860	tyrosine recombinase
	lilo_1167	LILO_RS06080	tyrosine recombinase
Operon_693	lilo_1125	LILO_RS05865	muramidase
Operon_694	lilo_1126	LILO_RS05870	RecA protein
	lilo_1127	LILO_RS05880	SOS responce UmuC protein
Operon_695	lilo_1128	LILO_RS05885	exonuclease
Operon_696	lilo_1129	LILO_RS05890	hypothetical protein
Operon_697	lilo_1130	LILO_RS05895	transposase of IS1077G
Operon_698	lilo_1131	LILO_RS05900	IS3/IS911transposase, N-terminal fragment
Operon_699	lilo_1132	LILO_RS05905	putative acyltransferase, family 3
Operon_700	lilo_1133	lilo_1133	peptidase T
Operon_701	lilo_1134	LILO_RS05915	molybdopterin biosynthesis protein MoeB
Operon_702	lilo_1135	lilo_1135	integral membrane protein TerC
	lilo_1136	LILO_RS05920	hypothetical protein
Operon_703	lilo_1137	lilo_1137	kap P-loop domain protein
Operon_704	lilo_1138	LILO_RS11740	type I restriction enzyme, S subunit
Operon_705	lilo_1139	lilo_1139	integrase
Operon_706	lilo_1140	LILO_RS05940	probable specificity determinant HsdS
	lilo_1141	LILO_RS05945	putative type I site-specific deoxyribonuclease
	lilo_1142	LILO_RS05950	type I restriction-modification system restrictionsubunit
Operon_707	lilo_1143	LILO_RS05955	adenine-specific DNA methylase-like protein
Operon_708	lilo_1144	LILO_RS05960	topoisomerase IV subunit B
Operon_709	lilo_1145	lilo_1145	relaxase
Operon_710	lilo_1146	lilo_1146	cystathionine beta-lyase / cystathionine gamma-lyase
Operon_711	lilo_1147	LILO_RS05975	ATPase for DNA repair
	lilo_1148	LILO_RS11745	LtrC protein
Operon_712	lilo_1149	LILO_RS05990	hypothetical protein
Operon_713	lilo_1150	LILO_RS05995	hypothetical protein
Operon_714	lilo_1151	LILO_RS06005	hypothetical protein
	lilo_1152	LILO_RS06010	hypothetical protein
	lilo_1153	LILO_RS06015	hypothetical protein
Operon_715	lilo_1154	lilo_1154	penicillin-binding protein
	lilo_1155	LILO_RS06025	hypothetical protein
Operon_716	lilo_1156	LILO_RS06035	conjugal transfer protein TraD
	lilo_1157	lilo_1157	replicative DNA helicase
Operon_717	lilo_1158	LILO_RS06045	hypothetical protein
	lilo_1159	lilo_1159	hypothetical protein
	lilo_1160	LILO_RS06050	cell surface antigen I/II precursor
Operon_718	lilo_1161	LILO_RS06055	hypothetical protein
Operon_719	lilo_1162	lilo_1162	two-component sensor histidine kinase
Operon_720	lilo_1163	LILO_RS06060	hypothetical protein
Operon_721	lilo_1164	lilo_1164	tRNA delta(2)-isopentenylpyrophosphate transferase

Operon_722	lilo_1165	LILO_RS06065	hypothetical protein
Operon_723	lilo_1166	LILO_RS06075	hypothetical protein
Operon_724	lilo_1168	lilo_1168	subtilisin-like serine protease
Operon_725	lilo_1169	LILO_RS06085	saccharopine dehydrogenase related protein
	lilo_1170	LILO_RS06090	intracellular protease/amidase
Operon_726	lilo_1171	LILO_RS06100	hypothetical protein
Operon_727	lilo_1172	lilo_1172	LysR family transcription regulator
Operon_728	lilo_1173	lilo_1173	LysR family transcription regulator
Operon_729	lilo_1174	lilo_1174	LysR family transcription regulator
Operon_730	lilo_1175	LILO_RS06115	N5-carboxyethyl-ornithine synthase
Operon_731	lilo_1176	LILO_RS06120	hypothetical protein
	lilo_1177	LILO_RS06125	hypothetical protein
	lilo_1178	LILO_RS06130	hypothetical protein
	lilo_0584	LILO_RS03150	N5-carboxyethyl-ornithine synthase
Operon_732	lilo_0580	LILO_RS03130	hypothetical protein
	lilo_1180	LILO_RS06140	hypothetical protein
Operon_733	lilo_1181	LILO_RS06145	oxidoreductase
Operon_734	lilo_1182	LILO_RS06150	glycerol uptake facilitator
	lilo_1183	LILO_RS06155	alpha-glycerophosphate oxidase
	lilo_1184	LILO_RS06160	glycerol kinase
Operon_735	lilo_1185	LILO_RS06165	trans-acting positive regulator
Operon_736	lilo_1186	LILO_RS06170	hypothetical protein
Operon_737	lilo_1187	LILO_RS06175	Na(+)/H(+) antiporter
Operon_738	lilo_1188	LILO_RS06180	hypothetical protein
Operon_739	lilo_1189	LILO_RS06185	alpha-amylase
Operon_740	lilo_1190	LILO_RS06190	L-lactate oxidase
Operon_741	lilo_1191	LILO_RS06195	phospho-2-dehydro-3-deoxyheptonate aldolase
Operon_742	lilo_1192	LILO_RS06200	5,10-methylenetetrahydrofolate reductase
	lilo_1193	LILO_RS06205	5-methionine synthase
Operon_743	lilo_1194	LILO_RS06210	iron-dependent repressor
Operon_744	lilo_1195	LILO_RS06215	cation-transporting P-ATPase
Operon_745	lilo_1196	LILO_RS06220	D-alanine transfer protein DltD
	lilo_1197	LILO_RS06225	D-alanyl carrier protein
	lilo_1198	LILO_RS06230	peptidoglycan biosynthesis protein
	lilo_1199	LILO_RS06235	D-alanine activating enzyme
	lilo_1200	lilo_1200	D-Ala-teichoic acid biosynthesis protein
Operon_746	lilo_1201	LILO_RS06240	thiamin-phosphate pyrophosphorylase
	lilo_1202	LILO_RS06245	phosphomethylpyrimidine kinase
	lilo_1203	LILO_RS06250	hydroxyethylthiazole kinase
Operon_747	lilo_1204	LILO_RS06255	glycosyl transferase
	lilo_1205	LILO_RS06260	UDP-N-acetylglucosamine 2-epimerase
Operon_748	lilo_1206	LILO_RS06265	hypothetical protein
Operon_749	lilo_1207	LILO_RS06270	lactococcin A ABC transporter ATP binding and permease protein
	lilo_1208	LILO_RS06275	response regulator of the LytR/AlgR family
	lilo_1209	LILO_RS06280	ABC transporter permease protein
Operon_750	lilo_1210	LILO_RS11750	phage Cro/CI transcriptional regulator

Operon_751	lilo_1211	LILO_RS06325	50S ribosomal protein L7/L12
	lilo_1212	LILO_RS06330	50S ribosomal protein L10
Operon_752	lilo_1213	LILO_RS06335	hypothetical protein
	lilo_1214	LILO_RS06340	transcription regulator
Operon_753	lilo_1215	LILO_RS06345	ABC transporter ATP binding and permease protein
	lilo_1216	LILO_RS06350	ABC transporter ATP binding and permease protein
	lilo_1217	LILO_RS06355	hypothetical protein
	lilo_1218	LILO_RS06360	hypothetical protein
Operon_754	lilo_1220	LILO_RS06370	pseudouridylate synthase
	lilo_1221	LILO_RS06375	segregation and condensation protein B
	lilo_1222	LILO_RS06380	segregation and condensation protein A
	lilo_1223	LILO_RS06385	site-specific tyrosine recombinase XerD
	lilo_1224	LILO_RS06390	cBS domain containing protein
	lilo_1225	LILO_RS06395	phosphoesterase family protein
	lilo_1226	LILO_RS06400	purine NTP pyrophosphatase
	lilo_1227	LILO_RS06405	glutamate racemase
Operon_755	lilo_1228	LILO_RS06410	hypothetical protein
Operon_756	lilo_1229	LILO_RS06415	diaminopimelate decarboxylase
Operon_757	lilo_1230	LILO_RS06420	glutamate synthase (NADPH) small chain
	lilo_1231	LILO_RS06425	glutamate synthase (NADPH) large chain
Operon_758	lilo_1232	LILO_RS06430	O-acetyltransferase
Operon_759	lilo_1233	LILO_RS06435	branched-chain amino acid aminotransferase
Operon_760	lilo_1234	LILO_RS06440	hypothetical protein
Operon_761	lilo_1236	LILO_RS06450	glutamate-gamma-aminobutyrate antiporter
Operon_762	lilo_1237	LILO_RS06455	positive regulator
Operon_763	lilo_1238	lilo_1238	N5-carboxyethyl-ornithine synthase
Operon_764	lilo_1239	LILO_RS06460	ribonuclease HII
Operon_765	lilo_1240	LILO_RS06465	GTP-binding protein
Operon_766	lilo_1241	LILO_RS06470	hypothetical protein
Operon_767	lilo_1242	LILO_RS06475	hypothetical protein
Operon_768	lilo_1243	LILO_RS06480	transcription regulator
	lilo_1244	LILO_RS06485	Xaa-Pro aminopeptidase
Operon_769	lilo_1245	LILO_RS06490	hypothetical protein
Operon_770	lilo_1246	lilo_1246	hypothetical protein
Operon_771	lilo_1248	LILO_RS06505	hypothetical secreted protein
	lilo_1249	lilo_1249	hypothetical protein
Operon_772	lilo_1250	LILO_RS06515	hypothetical protein
Operon_773	lilo_1251	LILO_RS06520	AguA protein
	lilo_1252	LILO_RS06525	putative polysaccharide deacetylase
	lilo_1253	LILO_RS06530	endoglucanase Y
	lilo_1254	LILO_RS06535	hypothetical protein
	lilo_1255	LILO_RS06540	hypothetical protein
Operon_774	lilo_1256	LILO_RS06545	sugar ABC transporter substrate-binding protein
	lilo_1257	LILO_RS06550	sialic acid-specific 9-O-acetylesterase
Operon_775	lilo_1258	LILO_RS06555	hypothetical protein
Operon_776	lilo_1259	LILO_RS06560	cation transport protein

Operon_777	lilo_1260	LILO_RS06565	phosphoglycerate mutase
Operon_778	lilo_1261	LILO_RS06570	putative hydrolase, haloacid dehalogenase family
Operon_779	lilo_1262	LILO_RS06575	putative hydrolase
	lilo_1263	LILO_RS06580	maltose O-acetyltransferase
	lilo_1264	LILO_RS06585	putative lactoylglutathione lyase
Operon_780	lilo_1265	LILO_RS06590	transcriptional regulator/sugar kinase , xylose operon regulator
Operon_781	lilo_1266	LILO_RS06595	indole-3-pyruvate decarboxylase
Operon_782	lilo_1267	LILO_RS06600	MarR family transcriptional regulator
	lilo_1268	LILO_RS06605	LysR family transcription regulator
Operon_783	lilo_1269	LILO_RS06610	hypothetical protein
Operon_784	lilo_1270	LILO_RS06615	hypothetical protein
Operon_785	lilo_1271	LILO_RS06625	short chain dehydrogenase
	lilo_1272	LILO_RS06630	transcription regulator
Operon_786	lilo_1273	LILO_RS06635	glycosyltransferase related enzyme
	lilo_1274	LILO_RS06640	phosphotransferase system, fructose-specific IIC component
	lilo_1275	LILO_RS06645	fusion of IIA, IIB and IIC component of mannitol/fructose-specific phosphotransferase system mannitol/fructose-specific
Operon_787	lilo_1276	LILO_RS06650	fructose/tagatose bisphosphate aldolase
Operon_788	lilo_1277	lilo_1277	hypothetical protein
Operon_789	lilo_1278	LILO_RS06660	hypothetical protein
Operon_790	lilo_1279	LILO_RS06665	hypothetical protein
Operon_791	lilo_1280	LILO_RS06670	arsenical resistance operon trans-acting repressor arsD
	lilo_1281	LILO_RS06675	arsenate reductase
Operon_792	lilo_1282	LILO_RS06680	para-aminobenzoate synthase component I
	lilo_1283	LILO_RS06685	para-aminobenzoate synthetase component II
Operon_793	lilo_1284	lilo_1284	ornithine cyclodeaminase, mu-crystallin homolog
Operon_794	lilo_1287	LILO_RS06705	manganese ABC transporter substrate binding protein
	lilo_1288	LILO_RS06710	manganese ABC transporter permease protein
	lilo_1289	LILO_RS06715	manganese ABC transporter ATP binding protein
Operon_795	lilo_1290	LILO_RS06720	hypothetical protein
Operon_796	lilo_1291	LILO_RS06725	hypothetical protein
Operon_797	lilo_1292	LILO_RS06730	hypothetical protein
Operon_798	lilo_1293	LILO_RS06735	ATP-dependent dsDNA exonuclease
	lilo_1294	LILO_RS06740	exonuclease SbcD
Operon_799	lilo_1295	LILO_RS06745	ketopantoate reductase
Operon_800	lilo_1296	LILO_RS06750	hypothetical protein
Operon_801	lilo_1297	lilo_1297	hypothetical protein
Operon_802	lilo_1298	LILO_RS06755	cell wall surface anchor family protein
Operon_803	lilo_1299	LILO_RS06760	hypothetical protein
Operon_804	lilo_1300	LILO_RS06765	fibronectin-binding protein
	lilo_1301	LILO_RS06770	hypothetical protein
Operon_805	lilo_1302	LILO_RS06775	sugar ABC transporter ATP binding protein
	lilo_1303	LILO_RS06780	transport and binding protein, carbohydrates
	lilo_1304	LILO_RS06785	uncharacterized ABC-type transport system, permeasecomponent

Operon_806	lilo_1305	LILO_RS06790	L-lactate dehydrogenase
Operon_807	lilo_1306	LILO_RS06795	pyruvate kinase
	lilo_1307	LILO_RS06800	6-phosphofructokinase
	lilo_1308	LILO_RS06805	hypothetical protein
Operon_808	lilo_1309	LILO_RS06810	N-acetylglucosamine-6-phosphate deacetylase
Operon_809	lilo_1310	LILO_RS06815	hypothetical protein
Operon_810	lilo_1311	LILO_RS06820	hypothetical protein
Operon_811	lilo_1312	LILO_RS06825	glycerol-3-phosphate dehydrogenase
	lilo_1313	LILO_RS06830	UTP-glucose-1-phosphate uridylyltransferase
Operon_812	lilo_1314	LILO_RS06835	hypothetical protein
	lilo_1315	LILO_RS06840	hypothetical protein
	lilo_1316	LILO_RS06845	heptaprenyl diphosphate synthase component II
	lilo_1317	LILO_RS06850	glucose-inhibited division protein GidB
Operon_813	lilo_1318	LILO_RS06855	hypothetical protein
	lilo_1319	LILO_RS06860	orotidine-phosphate decarboxylase
Operon_814	lilo_1320	LILO_RS06865	dihydroorotate dehydrogenase B
	lilo_1321	LILO_RS06870	dihydroorotate dehydrogenase electron transfer subunit
Operon_815	lilo_1322	LILO_RS06875	drug-export protein
Operon_816	lilo_1323	LILO_RS06880	hypothetical protein
Operon_817	lilo_1324	LILO_RS06885	hypothetical protein
	lilo_1325	LILO_RS06890	hypothetical protein
Operon_818	lilo_1326	LILO_RS06895	pseudouridine synthase
Operon_819	lilo_1328	LILO_RS06905	hypothetical protein
	lilo_1329	LILO_RS06910	hypothetical protein
	lilo_1330	LILO_RS06915	hypothetical protein
	lilo_1331	LILO_RS06920	hypothetical protein
	lilo_1332	LILO_RS06925	hypothetical protein
Operon_820	lilo_1333	LILO_RS06930	carbamoyl-phosphate synthase large chain
Operon_821	lilo_1334	LILO_RS06935	glutathione peroxidase
Operon_822	lilo_1335	LILO_RS06940	N-acetylmuramidase
Operon_823	lilo_1336	LILO_RS06945	cation-transporting ATPase
Operon_824	lilo_1337	LILO_RS06950	hypothetical protein
Operon_825	lilo_1338	LILO_RS06955	hypothetical protein
Operon_826	lilo_1339	LILO_RS06960	hypothetical protein
Operon_827	lilo_1340	LILO_RS06965	hypothetical protein
Operon_828	lilo_1341	LILO_RS06970	hypothetical protein
Operon_829	lilo_1342	lilo_1342	hypothetical protein
Operon_830	lilo_1343	LILO_RS06975	arsenate reductase
Operon_831	lilo_1344	lilo_1344	hypothetical protein
Operon_832	lilo_1345	lilo_1345	membrane protein
Operon_833	lilo_1346	lilo_1346	hypothetical protein
Operon_834	lilo_1348	lilo_1348	ABC-type amino acid transport/signal transduction system, periplasmic component/domain
Operon_835	lilo_1349	lilo_1349	transposase
Operon_836	lilo_1350	LILO_RS06995	hypothetical protein
Operon_837	lilo_1351	LILO_RS07000	two-component system regulator

	lilo_1352	LILO_RS07005	sensor protein kinase
Operon_838	lilo_1353	LILO_RS07010	hypothetical protein
Operon_839	lilo_1354	LILO_RS07015	GntR family transcriptional regulator
Operon_840	lilo_1355	LILO_RS07020	basic membrane protein A
Operon_841	lilo_1356	LILO_RS07025	cytidine deaminase
	lilo_1357	LILO_RS07030	deoxyribose-phosphate aldolase
	lilo_1358	LILO_RS07035	hypothetical protein
	lilo_1359	LILO_RS07040	pyrimidine-nucleoside phosphorylase
	lilo_1360	LILO_RS07045	hypothetical protein
Operon_842	lilo_1361	LILO_RS07050	pantothenate kinase
Operon_843	lilo_1362	LILO_RS07055	hypothetical protein
	lilo_1363	LILO_RS07060	cation transporter
Operon_844	lilo_1364	LILO_RS07065	transcription regulator
Operon_845	lilo_1365	LILO_RS07070	hypothetical protein
	lilo_1366	LILO_RS07075	predicted phosphoadenosine phosphosulfate sulfotransferase
Operon_846	lilo_1367	LILO_RS07080	transcription regulator
Operon_847	lilo_1368	LILO_RS07085	hypothetical protein
Operon_848	lilo_1369	LILO_RS07090	betaine ABC transporter permease and substrate binding protein
	lilo_1370	LILO_RS07095	betaine ABC transporter ATP binding protein
Operon_849	lilo_1371	LILO_RS07100	GntR family transcription regulator
Operon_850	lilo_1372	LILO_RS07105	lipopolysaccharide biosynthesis protein
	lilo_1373	LILO_RS07110	lipopolysaccharide biosynthesis protein
	lilo_1374	LILO_RS07115	hypothetical protein
Operon_851	lilo_1375	LILO_RS07120	hypothetical protein
Operon_852	lilo_1376	LILO_RS07125	hypothetical protein
Operon_853	lilo_1377	LILO_RS07130	beta-glucosidase
	lilo_1378	LILO_RS07135	beta-glucoside-specific PTS system IIABC component
	lilo_1379	LILO_RS07140	beta-glucoside operon antiterminator
Operon_854	lilo_1380	LILO_RS07145	tryptophan synthase alpha chain
	lilo_1381	LILO_RS07150	tryptophan synthase beta chain
	lilo_1382	LILO_RS07155	hypothetical protein
	lilo_1383	LILO_RS07160	phosphorybosyl-anthranilate isomerase
	lilo_1384	LILO_RS07165	indole-3-glycerol phosphate synthase
	lilo_1385	LILO_RS07170	predicted lactoylglutathione lyase
	lilo_1386	LILO_RS07180	anthranilate phosphoribosyltransferase
	lilo_1387	LILO_RS07185	anthranilate synthase component II
	lilo_1388	LILO_RS07190	anthranilate synthase component I
Operon_855	lilo_1389	LILO_RS07195	hypothetical protein
	lilo_1390	LILO_RS07200	transcription regulator
Operon_856	lilo_1391	LILO_RS07210	hypothetical protein
Operon_857	lilo_1392	LILO_RS07215	hypothetical protein
	lilo_1393	LILO_RS07220	hypothetical protein
Operon_858	lilo_1394	LILO_RS07225	ferric uptake regulator
	lilo_1395	LILO_RS07230	hypothetical protein
Operon_859	lilo_1396	LILO_RS07235	hypothetical protein

Operon_860	lilo_1397	LILO_RS07240	transcription regulator
Operon_861	lilo_1398	LILO_RS07245	oxidoreductase, short-chain dehydrogenase/reductasefamily protein
	lilo_1399	LILO_RS07250	1-deoxyxylulose-5-phosphate synthase
Operon_862	lilo_1400	LILO_RS07255	transcription regulator
Operon_863	lilo_1401	LILO_RS07260	cationic transporter
Operon_864	lilo_1402	LILO_RS07265	hypothetical protein
Operon_865	lilo_1403	LILO_RS07270	sugar transport symporter
Operon_866	lilo_1404	LILO_RS07275	GMP synthase
Operon_867	lilo_1405	LILO_RS07280	fructokinase
Operon_868	lilo_1406	LILO_RS07285	sugar kinase
	lilo_1407	LILO_RS07290	beta-glucosidase
	lilo_1408	LILO_RS07295	hypothetical protein
Operon_869	lilo_1409	LILO_RS07300	endo-beta-N-acetylglucosaminidase
	lilo_1410	LILO_RS07305	alpha 1-6-glucosidase
	lilo_1411	LILO_RS07310	lacto-N-biosidase
Operon_870	lilo_1412	LILO_RS07315	sugar ABC transporter substrate-binding protein
	lilo_1413	LILO_RS07320	sugar ABC transporter permease protein
	lilo_1414	LILO_RS07325	sugar ABC transporter substrate-binding protein
Operon_871	lilo_1415	LILO_RS07330	sugar hydrolase
	lilo_1416	LILO_RS07335	hypothetical protein
Operon_872	lilo_1417	LILO_RS07340	transcription regulator
	lilo_1418	LILO_RS07345	sugar hydrolase
Operon_873	lilo_1419	LILO_RS07350	putative phosphoketolase
Operon_874	lilo_1420	LILO_RS07355	D-xylose proton-symporter
	lilo_1421	lilo_1421	hypothetical protein
Operon_875	lilo_1424	LILO_RS07375	putative acetyltransferase
Operon_876	lilo_1425	LILO_RS07380	beta-1,4-xylosidase
	lilo_1426	LILO_RS07385	xyloside transporter
Operon_1400	lilo_1427	LILO_RS07390	aldose 1-epimerase
Operon_1401	lilo_1428	LILO_RS07395	xylulose kinase
	lilo_1429	LILO_RS07400	xylose isomerase
Operon_877	lilo_1430	LILO_RS07405	xylose operon regulator
Operon_878	lilo_1431	LILO_RS07410	phosphoribosylaminoimidazole carboxylase ATPase subunit
	lilo_1432	LILO_RS07415	phosphoribosylaminoimidazole carboxylase catalytic subunit
	lilo_1433	LILO_RS07425	phosphoribosylamine-glycine ligase
Operon_879	lilo_1434	LILO_RS07430	transcription regulator
Operon_880	lilo_1435	LILO_RS07435	transport protein
Operon_881	lilo_1436	LILO_RS11755	putative transcriptional regulator
Operon_882	lilo_1437	LILO_RS07445	multidrug transport protein
Operon_883	lilo_1438	LILO_RS07450	hypothetical protein
Operon_884	lilo_1439	LILO_RS07455	phosphoribosylaminoimidazolecarboxamide formyltransferase / IMP cyclohydrolase
Operon_885	lilo_1440	LILO_RS07460	hypoxanthine-guanine phosphoribosyltransferase
Operon_886	lilo_1441	LILO_RS07465	oxidoreductase
Operon_887	lilo_1442	LILO_RS07470	transcription regulator

Operon_888	lilo_1443	LILO_RS07475	ABC transporter ATP binding and permease protein
Operon_889	lilo_1444	LILO_RS07480	phosphoribosylglycinamide formyltransferase
	lilo_1445	LILO_RS07485	phosphoribosyl-aminoimidazole synthetase
Operon_890	lilo_1446	LILO_RS07490	type I site-specific deoxyribonuclease, HsdR subunit
Operon_891	lilo_1447	lilo_1447	hypothetical protein
Operon_892	lilo_1448	LILO_RS07500	type I restriction-modification system specificity subunit
	lilo_1449	LILO_RS07505	preprotein translocase subunit SecA
Operon_893	lilo_1450	LILO_RS07510	hypothetical protein
Operon_894	lilo_1451	lilo_1451	beta-phosphoglucomutase
	lilo_1452	lilo_1452	hypothetical protein
Operon_895	lilo_1453	LILO_RS07520	site-specific DNA-methyltransferase (adenine-specific), HsdM subunit
Operon_896	lilo_1454	LILO_RS07525	ClpB protein
	lilo_1455	LILO_RS07530	putative secreted protein
Operon_897	lilo_1456	LILO_RS07535	hypothetical protein
Operon_898	lilo_1457	LILO_RS07540	NADH dehydrogenase
Operon_899	lilo_1458	LILO_RS07545	phosphoribosylpyrophosphate amidotransferase
Operon_900	lilo_1459	LILO_RS07550	phosphoribosylformylglycinamidine synthase II
	lilo_1460	LILO_RS07555	phosphoribosylformylglycinamidine synthetase I
	lilo_1461	LILO_RS07560	hypothetical protein
	lilo_1462	LILO_RS07565	phosphoribosylaminoimidazole-succinocarboxamide synthetase
Operon_903	lilo_1463	LILO_RS07570	hypothetical protein
	lilo_1464	LILO_RS07575	hypothetical protein
	lilo_1465	LILO_RS07580	carboxymuconolactone decarboxylase family protein
	lilo_1466	LILO_RS07585	hypothetical protein
	lilo_1467	LILO_RS07590	oxidoreductase
Operon_904	lilo_1468	LILO_RS07595	transporter
	lilo_1469	LILO_RS07600	transcription regulator
Operon_905	lilo_1470	LILO_RS07605	thymidylate synthase
Operon_906	lilo_1471	LILO_RS07610	alcohol dehydrogenase
Operon_907	lilo_1472	LILO_RS07615	hypothetical protein
Operon_908	lilo_1473	LILO_RS07620	hypothetical protein
Operon_909	lilo_1474	LILO_RS07625	dihydroorotate dehydrogenase A
Operon_910	lilo_1475	LILO_RS07630	peptide methionine sulfoxide reductase
Operon_911	lilo_1476	LILO_RS07635	oxidoreductase
	lilo_1477	LILO_RS07640	hypothetical protein
	lilo_1478	LILO_RS07645	ABC transporter ATP binding protein
Operon_912	lilo_1479	LILO_RS07650	putative HTH-type transcriptional regulator CmhR
Operon_913	lilo_1480	LILO_RS07655	oxidoreductase
Operon_914	lilo_1481	LILO_RS07660	hypothetical protein
Operon_915	lilo_1482	LILO_RS07665	dipeptidase
Operon_916	lilo_1483	LILO_RS07670	poly(A) polymerase
Operon_917	lilo_1484	LILO_RS07675	hypothetical protein
Operon_918	lilo_1485	LILO_RS07680	dihydrodipicolinate reductase
Operon_919	lilo_1486	LILO_RS07685	hypothetical protein
	lilo_1487	LILO_RS07690	hypothetical protein

Operon_920	lilo_1488	LILO_RS07695	tRNA methyltransferase
	lilo_1489	LILO_RS07700	16S rRNA-processing protein RimM
Operon_921	lilo_1490	LILO_RS07705	hypothetical protein
Operon_922	lilo_1491	LILO_RS07710	ferrohelatase
Operon_923	lilo_1492	LILO_RS07715	hypothetical protein
Operon_924	lilo_1493	LILO_RS07720	30S ribosomal protein S16
Operon_925	lilo_1494	LILO_RS07725	hydroxymethylglutaryl-CoA reductase
Operon_926	lilo_1495	LILO_RS07730	acetyl coenzyme A acetyltransferase
Operon_927	lilo_1496	LILO_RS07735	hydroxymethylglutaryl-CoA synthase
Operon_928	lilo_1497	LILO_RS07740	glucosamine-6-P isomerase
Operon_929	lilo_1498	lilo_1498	hypothetical protein
	lilo_1499	lilo_1499	hypothetical protein
Operon_930	lilo_1500	LILO_RS07750	S-adenosylmethionine tRNA ribosyltransferase
Operon_931	lilo_1501	LILO_RS07755	transcription regulator
Operon_932	lilo_1502	LILO_RS07760	hypothetical protein
Operon_933	lilo_1503	LILO_RS07765	hypothetical protein
	lilo_1504	LILO_RS07770	hypothetical protein
	lilo_1505	LILO_RS07775	hypothetical protein
	lilo_1506	LILO_RS07780	cell surface protein
	lilo_1507	LILO_RS07785	hypothetical protein
Operon_934	lilo_1508	LILO_RS07790	hypothetical protein
Operon_935	lilo_1509	LILO_RS07795	hypothetical protein
Operon_936	lilo_1510	LILO_RS07800	hypothetical protein
	lilo_1511	LILO_RS07805	GTP-binding protein Obg
Operon_937	lilo_1512	LILO_RS07810	cell division protein FtsQ
	lilo_1513	LILO_RS07815	UDP-N-acetylglucosamine--N-acetylmuramyl-(pentapeptide) pyrophosphoryl-undecaprenol N-acetylglucosamine transferase
	lilo_1514	LILO_RS07820	UDP-N-acetylmuramoylalanine D-glutamate ligase
Operon_939	lilo_1515	LILO_RS07825	nitrogen regulatory protein P-II
	lilo_1516	LILO_RS07830	ammonium transporter
Operon_940	lilo_1517	LILO_RS07835	sensor protein kinase
	lilo_1518	LILO_RS07840	two-component system regulator
Operon_941	lilo_1519	LILO_RS07845	hypothetical protein
Operon_942	lilo_1520	LILO_RS07850	50S ribosomal protein L31
Operon_943	lilo_1521	LILO_RS07855	hypothetical protein
Operon_944	lilo_1522	LILO_RS07860	hypothetical protein
	lilo_1523	LILO_RS07865	surface antigen
Operon_945	lilo_1524	LILO_RS07870	hypothetical protein
Operon_946	lilo_1525	LILO_RS07875	hypothetical protein
Operon_947	lilo_1526	LILO_RS07880	carbamoyl-phosphate synthase small chain
	lilo_1527	LILO_RS07885	aspartate carbamoyltransferase catalytic chain
Operon_948	lilo_1528	LILO_RS07890	uracil permease
	lilo_1529	LILO_RS07895	pyrimidine operon attenuation protein / uracil phosphoribosyltransferase
Operon_949	lilo_1530	lilo_1530	hypothetical protein
Operon_950	lilo_1531	LILO_RS07905	transcription regulator

	lilo_1532	LILO_RS07910	cation transport protein
Operon_951	lilo_1533	LILO_RS07915	glutamate-5-semialdehyde dehydrogenase
	lilo_1534	LILO_RS07920	glutamate 5-kinase
Operon_952	lilo_1535	LILO_RS07925	hypothetical protein
Operon_953	lilo_1536	lilo_1536	hypothetical protein
Operon_954	lilo_1537	LILO_RS07940	branched-chain amino acid transport protein AzlC
Operon_955	lilo_1538	LILO_RS07945	hypothetical protein
Operon_956	lilo_1539	LILO_RS07950	spermidine acetyltransferase
Operon_957	lilo_1540	LILO_RS07955	signal recognition particle protein Ffh
Operon_958	lilo_1541	LILO_RS07960	hypothetical protein
Operon_959	lilo_1542	LILO_RS07965	hypothetical protein
Operon_960	lilo_1543	LILO_RS07970	cation transport ATPase
Operon_961	lilo_1544	LILO_RS07975	dihydrodipicolinate synthase
	lilo_1545	LILO_RS07980	hypothetical protein
	lilo_1546	LILO_RS07985	aspartate-semialdehyde dehydrogenase
Operon_962	lilo_1547	LILO_RS07990	transporter
Operon_963	lilo_1548	LILO_RS07995	hypothetical protein
Operon_964	lilo_1549	LILO_RS08000	transketolase
Operon_965	lilo_1550	LILO_RS08005	PTS system, unknown pentitol phosphotransferase enzyme IIC component
Operon_966	lilo_1551	LILO_RS08015	transcriptional antiterminator, BglG family
Operon_967	lilo_1552	LILO_RS08020	2-dehydro-3-deoxyphosphogluconate aldolase / 4-hydroxy-2-oxoglutarate aldolase
	lilo_1553	LILO_RS08025	2-dehydro-3-deoxygluconokinase
	lilo_1554	LILO_RS08030	glucuronate isomerase
	lilo_1555	LILO_RS08035	hypothetical protein
Operon_968	lilo_1556	LILO_RS08040	Na-galactoside symporter
Operon_969	lilo_1557	LILO_RS08045	D-mannonate dehydratase
Operon_970	lilo_1558	LILO_RS08050	fructuronate reductase
Operon_971	lilo_1559	LILO_RS08055	GntR family transcription regulator
Operon_972	lilo_1560	LILO_RS08060	multidrug transporter
Operon_973	lilo_1561	LILO_RS08070	cold shock protein CspD
Operon_974	lilo_1562	LILO_RS08075	fibrinogen-binding protein
Operon_975	lilo_1563	lilo_1563	chain length regulator
Operon_976	lilo_1564	LILO_RS08080	arsenate reductase
Operon_977	lilo_1565	LILO_RS08085	lyzozyme M1 (1,4-beta-N-acetylmuramidase)
Operon_978	lilo_1566	LILO_RS08095	hypothetical protein
	lilo_1567	LILO_RS11760	hypothetical protein
	lilo_1568	LILO_RS08110	hypothetical protein
	lilo_1569	LILO_RS08115	hypothetical protein
	lilo_1570	LILO_RS08120	SLT domain protein
	lilo_1571	LILO_RS08125	hypothetical protein
	lilo_1572	LILO_RS08130	hypothetical protein
Operon_979	lilo_1573	LILO_RS08135	major tail protein
	lilo_1574	LILO_RS08140	hypothetical protein
	lilo_1575	LILO_RS08145	hypothetical protein
	lilo_1576	LILO_RS08150	hypothetical protein

	lilo_1577	LILO_RS08155	hypothetical protein
	lilo_1578	LILO_RS08160	major head protein
	lilo_1579	LILO_RS08165	hypothetical protein
Operon_980	lilo_1580	LILO_RS08170	NAD+--asparagine ADP-ribosyltransferase
	lilo_1581	LILO_RS08175	predicted ribosomal protein
Operon_981	lilo_1582	LILO_RS08180	hypothetical protein
	lilo_1583	LILO_RS08185	terminase, large subunit, PBSX family
Operon_982	lilo_1584	LILO_RS08200	prophage pi1 protein 31
Operon_983	lilo_1585	lilo_1585	putative teicoplanin resistance protein
Operon_984	lilo_1586	lilo_1586	prophage pi2 protein 25
Operon_985	lilo_1587	lilo_1587	hypothetical protein
Operon_986	lilo_1588	lilo_1588	prophage pi2 protein 25
	lilo_1589	LILO_RS08205	hypothetical protein
Operon_987	lilo_1590	lilo_1590	hypothetical protein
Operon_988	lilo_1591	LILO_RS08220	prophage pi2 protein 24
	lilo_1592	LILO_RS08225	putative replication initiator protein
	lilo_1593	LILO_RS08230	hypothetical protein
	lilo_1594	lilo_1594	putative teichoic acid/polysaccharide export protein
	lilo_1595	LILO_RS08240	O6-methylguanine-DNA methyltransferase
	lilo_1596	lilo_1596	hypothetical protein
	lilo_1597	lilo_1597	hypothetical protein
	lilo_1598	LILO_RS08270	prophage pi3 protein 39
	lilo_1599	LILO_RS08275	prophage pi1 protein 15
Operon_989	lilo_1600	LILO_RS08285	replisome organizer
Operon_990	lilo_1601	LILO_RS08290	single-strand binding protein
	lilo_1602	LILO_RS08295	prophage replication protein
	lilo_1603	LILO_RS08300	hypothetical protein
Operon_991	lilo_1604	LILO_RS08305	hypothetical protein
Operon_992	lilo_1605	LILO_RS08310	6-phosphogluconolactonase
Operon_993	lilo_1606	LILO_RS08315	hypothetical protein
	lilo_1607	LILO_RS08320	hypothetical protein
	lilo_1608	LILO_RS08325	hypothetical protein
	lilo_1609	lilo_1609	hypothetical protein
Operon_994	lilo_1610	LILO_RS08335	alkylphosphonate ABC transporter permease component
Operon_995	lilo_1611	LILO_RS08340	cI-like repressor
	lilo_1612	LILO_RS08345	prophage pi3 protein 58
Operon_996	lilo_1613	LILO_RS08350	prophage pi3 protein 59
Operon_997	lilo_1614	LILO_RS08355	putative integrase
Operon_998	lilo_1615	LILO_RS08360	ribose ABC transporter substrate binding protein
	lilo_1616	LILO_RS08365	ribose ABC transporter permease protein
	lilo_1617	LILO_RS08370	ribose ABC transporter ATP binding protein
	lilo_1618	LILO_RS08375	ribose ABC transporter permease protein
	lilo_1619	LILO_RS08380	ribokinase
	lilo_1620	LILO_RS08385	ribose operon repressor
Operon_999	lilo_1621	LILO_RS08390	adenylosuccinate lyase
Operon_1000	lilo_1622	LILO_RS08395	3-dehydroquinate dehydratase

Operon_1001	lilo_1623	LILO_RS08405	hypothetical protein
Operon_1002	lilo_1624	LILO_RS08410	thioredoxin
Operon_1003	lilo_1625	LILO_RS08415	DNA mismatch repair protein
	lilo_1626	LILO_RS08420	hypothetical protein
Operon_1004	lilo_1627	LILO_RS08425	thioredoxin reductase
Operon_1005	lilo_1628	LILO_RS08430	catabolite control protein A
Operon_1006	lilo_1629	LILO_RS08435	proline dipeptidase
Operon_1007	lilo_1630	LILO_RS08440	hypothetical protein
Operon_1008	lilo_1631	LILO_RS08445	hypothetical protein
Operon_1009	lilo_1632	LILO_RS08455	hypothetical protein
Operon_1010	lilo_1633	LILO_RS08460	putative DNA polymerase III, delta subunit
	lilo_1634	LILO_RS08470	cell division protein FtsK
Operon_1011	lilo_1635	LILO_RS08475	putative hydrolase
Operon_1012	lilo_1636	LILO_RS08480	hypothetical protein
Operon_1013	lilo_1637	LILO_RS08485	phosphate acetyltransferase
Operon_1014	lilo_1638	LILO_RS08490	uridine kinase
Operon_1015	lilo_1639	LILO_RS08495	oxidoreductase
Operon_1016	lilo_1640	LILO_RS08505	hypothetical protein
Operon_1017	lilo_1641	LILO_RS08510	hypothetical protein
Operon_1018	lilo_1642	LILO_RS08515	hypothetical protein
Operon_1019	lilo_1643	LILO_RS08520	hypothetical protein
	lilo_1644	LILO_RS08525	hypothetical protein
Operon_1020	lilo_1645	LILO_RS08530	hypothetical protein
	lilo_1646	LILO_RS08535	hypothetical protein
Operon_1021	lilo_1647	LILO_RS08540	transcription regulator
	lilo_1648	LILO_RS08545	ubiquinone/menaquinone biosynthesis methyltransferase
Operon_1022	lilo_1649	LILO_RS08550	phage infection protein
Operon_1023	lilo_1650	LILO_RS08555	phospho-beta-glucosidase
Operon_1024	lilo_1651	LILO_RS08560	hypothetical protein
Operon_1025	lilo_1652	LILO_RS08565	1-deoxyxylulose-5-phosphate synthase
Operon_1026	lilo_1653	LILO_RS08570	sensor protein kinase
	lilo_1654	LILO_RS08575	two-component system regulator
Operon_1027	lilo_1655	LILO_RS08580	transcription regulator
Operon_1028	lilo_1656	LILO_RS08585	maltosephosphorylase
	lilo_1657	LILO_RS08590	alpha-glucosidase
	lilo_1658	LILO_RS08595	alpha-amylase
	lilo_1659	LILO_RS08600	galactoside O-acetyltransferase
	lilo_1660	LILO_RS08605	oligo-1,6-glucosidase
Operon_1029	lilo_1661	LILO_RS08610	neopullulanase
Operon_1030	lilo_1662	LILO_RS08615	maltose ABC transporter substrate binding protein
	lilo_1663	LILO_RS08620	maltose transport system permease protein MalF
	lilo_1664	LILO_RS08625	maltose ABC transporter permease protein
Operon_1031	lilo_1665	LILO_RS08630	hypothetical protein
Operon_1032	lilo_1666	lilo_1666	hypothetical protein
	lilo_1667	LILO_RS08640	hypothetical protein
Operon_1033	lilo_1668	LILO_RS08645	hypothetical protein

Operon_1034	lilo_1669	LILO_RS08650	hypothetical protein
Operon_1035	lilo_1670	LILO_RS08655	hypothetical protein
Operon_1036	lilo_1671	LILO_RS08660	transcription regulator
Operon_1037	lilo_1672	LILO_RS08665	NADH-dependent oxidoreductase
Operon_1038	lilo_1673	LILO_RS08670	carbamate kinase
	lilo_1674	LILO_RS08675	hypothetical protein
	lilo_1675	LILO_RS08680	amino acid antiporter
	lilo_1676	LILO_RS08685	ornithine carbamoyltransferase
Operon_1039	lilo_1677	LILO_RS08690	two-component system regulator
Operon_1040	lilo_1678	LILO_RS08695	transcription regulator
Operon_1041	lilo_1679	LILO_RS08700	cytidine monophosphate kinase
Operon_1042	lilo_1681	LILO_RS08710	ferredoxin
Operon_1043	lilo_1682	LILO_RS08715	mannose-specific PTS system component IIAB
	lilo_1683	LILO_RS08720	mannose-specific PTS system component IIC
	lilo_1684	LILO_RS08725	mannose-specific PTS system component IID
	lilo_1685	LILO_RS08730	hypothetical protein
Operon_1045	lilo_1686	LILO_RS08735	hypothetical protein
Operon_1046	lilo_1687	LILO_RS08740	hypothetical protein
Operon_1047	lilo_1688	LILO_RS08745	seryl-tRNA synthetase
Operon_1048	lilo_1689	LILO_RS08750	hypothetical protein
	lilo_1690	LILO_RS08755	major facilitator superfamily permease
	lilo_1691	LILO_RS08760	ABC-2 type transporter
	lilo_1692	LILO_RS08765	ABC transporter related
	lilo_1693	lilo_1693	hypothetical protein
Operon_1049	lilo_1694	LILO_RS08780	hypothetical protein
	lilo_1695	LILO_RS08785	hypothetical protein
Operon_1052	lilo_1696	LILO_RS08790	phosphate transport system regulator
	lilo_1697	LILO_RS08795	phosphate ABC transporter ATP-binding protein
	lilo_1698	LILO_RS08800	phosphate ABC transporter ATP-binding protein
	lilo_1699	LILO_RS08805	phosphate transport system permease protein
	lilo_1700	LILO_RS08810	phosphate ABC transporter permease protein
	lilo_1701	LILO_RS08815	phosphate ABC transporter substrate binding protein
	lilo_1702	LILO_RS08820	phosphate ABC transporter substrate binding protein
Operon_1055	lilo_1703	LILO_RS08825	tmRNA-binding protein SmpB
	lilo_1704	LILO_RS08830	hypothetical protein
Operon_1056	lilo_1705	LILO_RS08835	alanyl-tRNA synthetase
Operon_1057	lilo_1706	LILO_RS08840	maturation protein
Operon_1058	lilo_1707	LILO_RS08845	hypothetical protein
	lilo_1708	LILO_RS08850	oligoendopeptidase F
	lilo_1709	LILO_RS08855	competence protein CoiA
Operon_1059	lilo_1710	LILO_RS08860	hypothetical protein
Operon_1060	lilo_1711	LILO_RS08865	hypothetical protein
Operon_1061	lilo_1712	LILO_RS08870	hypothetical protein
Operon_1062	lilo_1713	LILO_RS08875	hypothetical protein
Operon_1063	lilo_1714	LILO_RS08880	oxidoreductase
	lilo_1715	LILO_RS08885	iron-binding oxidase subunit

	lilo_1716	LILO_RS08890	hypothetical protein
Operon_1064	lilo_1717	LILO_RS08895	transport permease
Operon_1065	lilo_1718	LILO_RS08900	hypothetical protein
Operon_1066	lilo_1719	LILO_RS08905	hypothetical protein
Operon_1067	lilo_1720	LILO_RS08910	30S ribosomal protein S20
Operon_1068	lilo_1721	LILO_RS08915	exodeoxyribonuclease V alpha chain
	lilo_1722	LILO_RS08920	phosphoglycerate mutase
	lilo_1723	LILO_RS08925	prephenate dehydratase
	lilo_1724	LILO_RS08930	shikimate kinase
	lilo_1725	LILO_RS08935	3-phosphoshikimate 1-carboxyvinyltransferase
Operon_1069	lilo_1726	LILO_RS08940	prephenate dehydrogenase
	lilo_1727	LILO_RS08945	sensor protein kinase
	lilo_1728	LILO_RS08950	two-component system regulator
	lilo_1729	LILO_RS08955	hypothetical protein
Operon_1070	lilo_1730	LILO_RS08960	ABC transporter permease and substrate binding protein
	lilo_1731	LILO_RS08965	ABC transporter ATP-binding protein
	lilo_1732	LILO_RS08970	hypothetical protein
Operon_1071	lilo_1733	LILO_RS08975	chorismate synthase
	lilo_1734	LILO_RS08980	hypothetical protein
	lilo_1735	LILO_RS08985	hypothetical protein
Operon_1072	lilo_1736	LILO_RS08990	hypothetical protein
Operon_1073	lilo_1737	LILO_RS08995	3-dehydroquinate synthase
	lilo_1738	LILO_RS09000	shikimate 5-dehydrogenase
Operon_1074	lilo_1739	LILO_RS09005	hypothetical protein
Operon_1075	lilo_1740	LILO_RS09010	glutamine ABC transporter permease and substrate binding protein
	lilo_1741	LILO_RS09015	glutamine ABC transporter ATP-binding protein
Operon_1076	lilo_1742	lilo_1742	hypothetical protein
	lilo_1743	LILO_RS09025	hypothetical protein
Operon_1077	lilo_1744	LILO_RS09030	ATP synthase epsilon subunit
Operon_1078	lilo_1745	LILO_RS09035	ATP synthase alpha subunit
Operon_1079	lilo_1746	LILO_RS09040	ATP synthase gamma subunit
	lilo_1747	LILO_RS09045	ATP synthase alpha subunit
Operon_1080	lilo_1748	LILO_RS09050	ATP synthase delta subunit
	lilo_1749	LILO_RS09055	ATP synthase subunit b
	lilo_1750	LILO_RS09060	ATP synthase subunit a
	lilo_1751	LILO_RS09065	F0F1-type ATP synthase C subunit
Operon_1081	lilo_1752	lilo_1752	hypothetical protein
Operon_1082	lilo_1753	LILO_RS09070	lipase
Operon_1083	lilo_1754	LILO_RS09075	competence protein ComEC
	lilo_1755	LILO_RS09080	competence protein ComEA
	lilo_1756	LILO_RS09085	ABC transporter permease protein
	lilo_1757	LILO_RS09090	sodium transport system ATP-binding protein
Operon_1084	lilo_1758	LILO_RS09095	hypothetical protein
Operon_1085	lilo_1759	LILO_RS09100	acyltransferase
Operon_1086	lilo_1760	LILO_RS09105	cation transporter

Operon_1087	lilo_1761	LILO_RS09110	transcriptional regulator TenA
	lilo_1762	LILO_RS09115	hypothetical protein
	lilo_1763	LILO_RS09120	bifunctional protein BirA
	lilo_1764	LILO_RS09125	putative biotin biosynthesis protein BioY
Operon_1088	lilo_1765	LILO_RS09130	hypothetical protein
	lilo_1766	LILO_RS09135	acetyl-CoA C-acetyltransferase
	lilo_1767	LILO_RS09140	hypothetical protein
	lilo_1768	LILO_RS09145	hypothetical protein
	lilo_1769	LILO_RS09150	3-oxoacyl-acyl carrier protein reductase
	lilo_1770	LILO_RS09155	2-nitropropane deoxygenase
Operon_1089	lilo_1771	LILO_RS09160	hypothetical protein
Operon_1090	lilo_1772	LILO_RS09165	NifU protein
Operon_1091	lilo_1773	LILO_RS09170	hypothetical protein
	lilo_1774	LILO_RS09175	putative aminotransferase
	lilo_1775	LILO_RS09180	hypothetical protein
	lilo_1776	LILO_RS09185	ABC transporter ATP-binding protein
Operon_1092	lilo_1777	LILO_RS09190	polysacharide biosynthesis protein
	lilo_1778	LILO_RS09195	regulatory protein
Operon_1093	lilo_1779	LILO_RS09200	glutamate ABC transporter ATP-binding protein
	lilo_1780	LILO_RS09205	glutamate ABC transporter permease protein
Operon_1094	lilo_1781	LILO_RS09210	transcription regulator
Operon_1095	lilo_1782	LILO_RS09215	DNA-directed RNA polymerase beta' chain
	lilo_1783	LILO_RS09220	DNA-directed RNA polymerase beta chain
Operon_1096	lilo_1784	LILO_RS09225	transcription regulator
Operon_1097	lilo_1785	LILO_RS09230	transcription regulator
Operon_1098	lilo_1786	LILO_RS09235	neutral endopeptidase
Operon_1099	lilo_1787	LILO_RS09240	hypothetical protein
Operon_1100	lilo_1788	LILO_RS09245	hypothetical protein
Operon_1101	lilo_1789	LILO_RS09250	UDP-MurNac-tripeptide synthetase
Operon_1102	lilo_1790	LILO_RS09260	alcohol dehydrogenase
Operon_1103	lilo_1791	LILO_RS09265	ATP-dependent DNA helicase RecQ
Operon_1104	lilo_1792	LILO_RS09270	putative amino acid permease
	lilo_1793	LILO_RS09275	peptidase T
	lilo_1794	LILO_RS09280	hypothetical protein
Operon_1377	lilo_1795	LILO_RS09285	inorganic pyrophosphatase
Operon_1105	lilo_1796	LILO_RS09290	pyruvate-formate lyase activating enzyme
Operon_1106	lilo_1797	LILO_RS09295	transport protein
Operon_1107	lilo_1798	LILO_RS09300	permease
Operon_1108	lilo_1800	LILO_RS09315	aldo/keto reductase
Operon_1109	lilo_1801	LILO_RS09320	universal stress protein UspA related nucleotide-binding protein
Operon_1110	lilo_1802	LILO_RS09325	hypothetical protein
Operon_1111	lilo_1803	LILO_RS09330	hypothetical protein
Operon_1112	lilo_1804	lilo_1804	prophage ps3 protein 4
Operon_1113	lilo_1805	LILO_RS09335	-
Operon_1114	lilo_1805	LILO_RS09335	hypothetical protein
	lilo_1806	LILO_RS09340	prophage ps1 protein 1

Operon_1115	lilo_1807	LILO_RS09345	prophage ps2 protein 18
	lilo_1808	LILO_RS09350	major head protein precursor
Operon_1116	lilo_1809	LILO_RS09355	prophage ps2 protein 16
	lilo_1810	LILO_RS09360	oxidoreductase
Operon_1117	lilo_1811	LILO_RS09365	prophage ps2 protein 15
	lilo_1812	LILO_RS09370	prophage ps2 protein 14
	lilo_1813	LILO_RS09375	hypothetical protein
	lilo_1814	LILO_RS09380	hypothetical protein
	lilo_1815	lilo_1815	putative metalloendopeptidase, glycoprotease family
	lilo_1816	LILO_RS09385	prophage ps1 protein 13
Operon_1118	lilo_1817	LILO_RS09390	hypothetical protein
Operon_1119	lilo_1818	LILO_RS09395	transcriptional regulator, xre family
Operon_1120	lilo_1819	LILO_RS09400	integrase
Operon_1121	lilo_1820	LILO_RS09405	hypothetical protein
Operon_1122	lilo_1821	LILO_RS09410	hypothetical protein
Operon_1123	lilo_1822	LILO_RS09415	excinuclease ABC subunit A
Operon_1124	lilo_1823	LILO_RS09420	hypothetical protein
Operon_1125	lilo_1824	LILO_RS09425	alpha-ribazole-5'-phosphate phosphatase
	lilo_1825	LILO_RS09430	putative endonuclease
Operon_1126	lilo_1826	LILO_RS09435	amino acid permease
Operon_1127	lilo_1827	LILO_RS09440	oxidoreductase
	lilo_1828	LILO_RS09445	hypothetical protein
	lilo_1829	LILO_RS09450	hypothetical protein
	lilo_1830	LILO_RS09455	hypothetical protein
Operon_1128	lilo_1831	LILO_RS09460	asparaginyl-tRNA synthetase
	lilo_1832	LILO_RS09465	hypothetical protein
	lilo_1833	LILO_RS09470	aspartate aminotransferase
	lilo_1834	LILO_RS09475	hypothetical protein
Operon_1129	lilo_1835	LILO_RS09480	ATP-dependent helicase DinG
Operon_1130	lilo_1836	LILO_RS09485	hypothetical protein
Operon_1131	lilo_1837	LILO_RS09490	transport and binding protein
Operon_1132	lilo_1838	LILO_RS09495	penicillin acylase
Operon_1133	lilo_1839	LILO_RS09500	tyrosine phosphatase
Operon_1135	lilo_1840	LILO_RS09505	oligopeptide ABC transporter substrate binding protein
	lilo_1841	LILO_RS09510	oligopeptide ABC transporter permease protein
	lilo_1842	LILO_RS09515	oligopeptide ABC transporter permease protein
	lilo_1843	LILO_RS09520	oligopeptide ABC transporter ATP binding protein
	lilo_1844	LILO_RS09525	oligopeptide ABC transporter ATP binding protein
Operon_1137	lilo_1845	LILO_RS09530	50S ribosomal protein L20
	lilo_1846	LILO_RS09535	50S ribosomal protein L35
Operon_1138	lilo_1847	LILO_RS09540	translation initiation factor IF-3
Operon_1139	lilo_1848	lilo_1848	hypothetical protein
	lilo_1849	LILO_RS09545	hypothetical protein
Operon_1140	lilo_1850	lilo_1850	hypothetical protein
Operon_1141	lilo_1851	LILO_RS09555	glucose inhibited division protein GidA
Operon_1142	lilo_1852	LILO_RS09560	hypothetical protein

Operon_1143	lilo_1853	LILO_RS09565	hypothetical protein
Operon_1144	lilo_1854	LILO_RS09570	hypothetical protein
	lilo_1855	LILO_RS09575	cysteinyl-tRNA synthetase
	lilo_1856	LILO_RS09580	hypothetical protein
	lilo_1857	LILO_RS09585	serine acetyltransferase
Operon_1145	lilo_1858	LILO_RS09590	hypothetical protein
Operon_1146	lilo_1859	LILO_RS09595	polyribonucleotide nucleotidyltransferase
Operon_1147	lilo_1860	LILO_RS09600	hypothetical protein
Operon_1148	lilo_1861	LILO_RS09605	ribose-phosphate pyrophosphokinase
Operon_1149	lilo_1862	LILO_RS09610	hypothetical protein
Operon_1150	lilo_1863	LILO_RS09615	pyridoxal-phosphate dependent aminotransferase NifS
	lilo_1864	LILO_RS09620	hypothetical protein
Operon_1151	lilo_1865	LILO_RS09625	elongation factor EF-Tu
Operon_1152	lilo_1866	LILO_RS09630	hypothetical protein
Operon_1153	lilo_1867	LILO_RS09635	isoleucyl-tRNA synthetase
Operon_1154	lilo_1868	LILO_RS09640	cell division protein
	lilo_1869	LILO_RS09645	hypothetical protein
	lilo_1870	LILO_RS09650	YggT family protein
	lilo_1871	LILO_RS09655	hypothetical protein
	lilo_1872	LILO_RS09660	hypothetical protein
	lilo_1873	LILO_RS09665	cell division protein FtsZ
	lilo_1874	LILO_RS09670	cell division protein FtsA
Operon_1155	lilo_1875	LILO_RS09675	hypothetical protein
	lilo_1876	LILO_RS09680	tRNA-guanosine methyltransferase
Operon_1156	lilo_1877	LILO_RS09685	hypothetical protein
Operon_1157	lilo_1878	LILO_RS09690	oxidoreductase
	lilo_1879	LILO_RS09695	transmembrane efflux protein
Operon_1158	lilo_1880	LILO_RS09700	LysR family transcription regulator
	lilo_1881	LILO_RS09705	transcription regulator
Operon_1159	lilo_1882	LILO_RS09710	PepC protein
Operon_1160	lilo_1883	LILO_RS09715	hypothetical protein
	lilo_1884	LILO_RS09720	5'-methylthioadenosine/S-adenosylhomocysteine nucleosidase
	lilo_1885	LILO_RS09725	hypothetical protein
	lilo_1886	LILO_RS09730	ADP-ribose pyrophosphatase
	lilo_1887	LILO_RS09735	glucosamine-1-phosphate N-acetyltransferase / UDP-N-acetylglucosamine pyrophosphorylase
Operon_1161	lilo_1888	LILO_RS09740	pyrroline-5-carboxylate reductase
Operon_1162	lilo_1889	LILO_RS09745	30S ribosomal protein S15
Operon_1163	lilo_1890	LILO_RS09750	serine/threonine protein kinase
	lilo_1891	LILO_RS09755	putative PP2C protein phosphatase
	lilo_1892	LILO_RS09760	rRNA methylase
Operon_1164	lilo_1893	LILO_RS09765	hypothetical protein
	lilo_1894	LILO_RS09775	hypothetical protein
	lilo_1895	LILO_RS09780	hypothetical protein
Operon_1165	lilo_1896	LILO_RS09785	methyonyl-tRNA formyltransferase
	lilo_1897	LILO_RS09790	hypothetical protein

	lilo_1898	LILO_RS09795	hypothetical protein
	lilo_1899	LILO_RS09800	hypothetical protein
Operon_1166	lilo_1900	LILO_RS09805	primosomal protein N'
Operon_1167	lilo_1901	LILO_RS09810	DNA-directed RNA polymerase subunit omega
Operon_1168	lilo_1902	LILO_RS09815	guanylate kinase
Operon_1169	lilo_1903	LILO_RS09820	hypothetical protein
Operon_1170	lilo_1904	LILO_RS09825	hypothetical protein
Operon_1171	lilo_1905	lilo_1905	hypothetical protein
	lilo_1906	LILO_RS09835	S-adenosylmethionine synthetase
Operon_1172	lilo_1907	LILO_RS09840	cyclopropane fatty acid synthase related methyltransferase
Operon_1173	lilo_1908	LILO_RS09845	acetyl-CoA carboxylase ligase / biotin operon repressor bifunctional protein
Operon_1174	lilo_1909	LILO_RS09850	hypothetical protein
	lilo_1910	LILO_RS09855	hypothetical protein
	lilo_1911	LILO_RS09860	hypothetical protein
Operon_1175	lilo_1912	LILO_RS09865	N-acetylmuramidase
Operon_1176	lilo_1913	LILO_RS09870	fructose-bisphosphate aldolase
Operon_1177	lilo_1914	LILO_RS09930	hypothetical protein
Operon_1178	lilo_1915	LILO_RS09935	theronyl-tRNA synthetase
Operon_1179	lilo_1916	lilo_1916	maltose phosphorylase
Operon_1180	lilo_1917	LILO_RS09940	hypothetical protein
	lilo_1918	LILO_RS09945	putative Mn and Fe transporters
Operon_1181	lilo_1919	LILO_RS09950	hypothetical protein
Operon_1182	lilo_1920	LILO_RS09955	Upp protein
Operon_1183	lilo_1921	LILO_RS09960	Na+/H+ antiporter
	lilo_1922	LILO_RS09965	aminotransferase
Operon_1184	lilo_1923	LILO_RS09970	cystathionine gamma-synthase
	lilo_1924	LILO_RS09975	homoserine O-succinyltransferase
Operon_1185	lilo_1925	LILO_RS09980	hypothetical protein
	lilo_1926	LILO_RS09985	hypothetical protein
Operon_1186	lilo_1927	LILO_RS09990	galactose mutarotase related enzyme
Operon_1187	lilo_1928	LILO_RS09995	putative CMP-binding factor
Operon_1188	lilo_1929	LILO_RS10000	hypothetical protein
Operon_1189	lilo_1930	LILO_RS10005	ribulose-phosphate 3-epimerase
Operon_1190	lilo_1931	LILO_RS10010	hypothetical protein
	lilo_1932	LILO_RS10020	hypothetical protein
Operon_1191	lilo_1933	LILO_RS10025	hypothetical protein
Operon_1192	lilo_1934	LILO_RS10030	phenylalanyl-tRNA synthetase beta chain
	lilo_1935	LILO_RS10035	phenylalanyl-tRNA synthetase alpha chain
Operon_1193	lilo_1936	LILO_RS10040	phenolic acid decarboxylase
Operon_1194	lilo_1937	LILO_RS10045	hypothetical protein
Operon_1195	lilo_1938	LILO_RS10050	chitinase
	lilo_1939	LILO_RS10055	chitin binding protein
Operon_1196	lilo_1940	LILO_RS10060	adenylosuccinate synthase
Operon_1197	lilo_1941	LILO_RS10065	transport protein
Operon_1198	lilo_1942	LILO_RS10070	hypothetical protein

Operon_1199	lilo_1943	lilo_1943	hypothetical protein
Operon_1200	lilo_1944	LILO_RS10080	hypothetical protein
Operon_1201	lilo_1945	LILO_RS10085	hypothetical protein
Operon_1202	lilo_1946	LILO_RS10090	hypothetical protein
Operon_1203	lilo_1947	LILO_RS10095	hypothetical protein
Operon_1204	lilo_1948	LILO_RS10100	putative heat shock protein
	lilo_1949	LILO_RS10105	hypothetical protein
Operon_1205	lilo_1950	LILO_RS10110	oxidoreductase
Operon_1206	lilo_1951	LILO_RS10120	hypothetical protein
Operon_1207	lilo_1952	LILO_RS10125	hypothetical protein
	lilo_1953	LILO_RS10130	hypothetical protein
	lilo_1954	LILO_RS10135	aspartyl-tRNA synthetase
	lilo_1955	LILO_RS10140	diadenosine tetraphosphate (Ap4A) hydrolase relatedHIT family hydrolase
	lilo_1956	LILO_RS10145	histidyl-tRNA synthetase
Operon_1208	lilo_1957	LILO_RS10150	hypothetical protein
Operon_1209	lilo_1958	LILO_RS10155	hypothetical protein
Operon_1210	lilo_1959	LILO_RS10160	hypothetical protein
Operon_1211	lilo_1960	LILO_RS10165	CDP-diacylglycerol-phosphate phosphatidyltransferase
	lilo_1961	LILO_RS10170	hypothetical protein
Operon_1212	lilo_1962	LILO_RS10175	putative protease
	lilo_1963	LILO_RS10180	putative protease
Operon_1213	lilo_1964	LILO_RS10185	hypothetical protein
	lilo_1965	LILO_RS10190	RecF protein
Operon_1214	lilo_1966	LILO_RS10195	gamma-carboxymuconolactone decarboxylase
Operon_1215	lilo_1967	LILO_RS10200	transcription regulator
Operon_1216	lilo_1968	LILO_RS10205	hypothetical protein
Operon_1217	lilo_1969	LILO_RS10210	UDP-glucose 4-epimerase
	lilo_1970	LILO_RS10215	galactose-1-phosphate uridylyltransferase
Operon_1218	lilo_1971	LILO_RS10220	galactokinase
	lilo_1972	LILO_RS10225	aldose 1-epimerase
Operon_1219	lilo_1973	LILO_RS10230	galactose permease
Operon_1220	lilo_1974	LILO_RS10235	transcription regulator
Operon_1221	lilo_1975	LILO_RS10240	oxidoreductase of aryl-alcohol dehydrogenase family
Operon_1222	lilo_1976	LILO_RS10245	transcriptional regulator
	lilo_1977	LILO_RS10250	dehydrogenase
Operon_1223	lilo_1978	LILO_RS10255	protease
Operon_1224	lilo_1979	LILO_RS10260	hypothetical protein
Operon_1225	lilo_1980	LILO_RS10270	protease
Operon_1226	lilo_1981	LILO_RS10275	hypothetical protein
Operon_1227	lilo_1982	LILO_RS10280	rRNA (guanine-N1-)-methyltransferase, putative
Operon_1228	lilo_1983	LILO_RS10285	putative copper homeostasis protein
Operon_1229	lilo_1984	LILO_RS10290	ABC transporter permease protein
	lilo_1985	LILO_RS10295	ABC transporter ATP binding protein
	lilo_1986	LILO_RS10300	putative glycerophosphoryl diester phosphodiesterase
Operon_1230	lilo_1987	LILO_RS10305	putative cell-cycle regulation histidine triad

	lilo_1988	LILO_RS10310	hypothetical protein
Operon_1231	lilo_1989	LILO_RS10315	hypothetical protein
Operon_1233	lilo_1990	LILO_RS10320	50S ribosomal protein L1
	lilo_1991	LILO_RS10325	50S ribosomal protein L11
Operon_1234	lilo_1992	LILO_RS10330	metal transporting ATPase
Operon_1235	lilo_1993	LILO_RS10335	transcriptional regulator
Operon_1236	lilo_1994	LILO_RS10340	hypothetical protein
	lilo_1995	LILO_RS10345	peptide methionine sulfoxide reductase
Operon_1237	lilo_1996	LILO_RS10350	hypothetical protein
	lilo_1997	LILO_RS10355	hypothetical protein
Operon_1238	lilo_1998	LILO_RS10360	ribosome recycling factor
Operon_1239	lilo_1999	LILO_RS10365	UMP-kinase
Operon_1240	lilo_2000	LILO_RS10370	hypothetical protein
Operon_1241	lilo_2001	LILO_RS10375	acetate kinase
Operon_1242	lilo_2002	LILO_RS10380	acetate kinase
Operon_1243	lilo_2003	LILO_RS10385	hypothetical protein
Operon_1244	lilo_2004	LILO_RS10390	hypothetical protein
Operon_1245	lilo_2005	LILO_RS10395	GTP-binding protein TypA/BipA
Operon_1246	lilo_2006	LILO_RS10400	hypothetical protein
	lilo_2007	LILO_RS10405	hypothetical protein
	lilo_2008	LILO_RS10410	hypothetical protein
Operon_1247	lilo_2009	LILO_RS10415	ABC transporter ATP binding protein
	lilo_2010	LILO_RS10420	hypothetical protein
	lilo_2011	LILO_RS10425	hypothetical protein
Operon_1248	lilo_2012	LILO_RS10430	hypothetical protein
Operon_1249	lilo_2013	LILO_RS10435	hypothetical protein
	lilo_2014	LILO_RS10440	glucose kinase
	lilo_2015	LILO_RS10445	hypothetical protein
Operon_1250	lilo_2016	LILO_RS10450	non-heme iron-binding ferritin
Operon_1251	lilo_2017	LILO_RS10455	leader peptidase (prepilin peptidase) / N-methyltransferase
Operon_1252	lilo_2018	LILO_RS10460	DNA polymerase IV
Operon_1253	lilo_2019	LILO_RS10465	hypothetical protein
Operon_1254	lilo_2020	LILO_RS10470	arginine/ornitine antiporter
	lilo_2021	LILO_RS10475	hypothetical protein
	lilo_2022	LILO_RS10480	carbamate kinase
	lilo_2023	LILO_RS10485	carbamate kinase
	lilo_2024	LILO_RS10490	arginine/ornithine antiporter
Operon_1378	lilo_2025	LILO_RS10495	ornithine carbamoyltransferase
	lilo_2026	LILO_RS10500	arginine deiminase
Operon_1259	lilo_2027	LILO_RS10510	arginyl-tRNA synthetase
Operon_1260	lilo_2028	LILO_RS10515	arginine catabolic regulator
Operon_1261	lilo_2029	LILO_RS10520	UDP-N-acetylmuramate-alanine ligase
Operon_1262	lilo_2030	LILO_RS10525	hypothetical protein
Operon_1263	lilo_2031	LILO_RS10530	SWI/SNF family helicase
Operon_1264	lilo_2032	LILO_RS10535	hypothetical protein
Operon_1265	lilo_2033	LILO_RS10540	pyruvate oxidase

Operon_1266	lilo_2034	LILO_RS10545	hypothetical protein
Operon_1267	lilo_2035	lilo_2035	hypothetical protein
Operon_1268	lilo_2036	LILO_RS10555	hypothetical protein
Operon_1269	lilo_2037	LILO_RS10560	glycerol uptake facilitator related permease
Operon_1270	lilo_2038	LILO_RS10565	X-prolyl dipeptidyl aminopeptidase
	lilo_2039	LILO_RS10570	hypothetical protein
Operon_1271	lilo_2040	LILO_RS10575	amino acid ABC transporter substrate binding protein
Operon_1272	lilo_2041	LILO_RS10580	glutamyl-tRNA synthetase
Operon_1273	lilo_2042	LILO_RS10585	hypothetical protein
Operon_1274	lilo_2043	LILO_RS10590	hypothetical protein
Operon_1275	lilo_2044	LILO_RS10595	hypothetical protein
Operon_1276	lilo_2045	LILO_RS10600	hypothetical protein
	lilo_2046	LILO_RS10605	hypothetical protein
	lilo_2047	LILO_RS10610	hypothetical protein
Operon_1277	lilo_2048	LILO_RS10615	hypothetical protein
Operon_1278	lilo_2049	LILO_RS10620	hypothetical protein
Operon_1279	lilo_2050	LILO_RS10625	hypothetical protein
Operon_1280	lilo_2051	LILO_RS10630	DNA repair protein
Operon_1281	lilo_2052	LILO_RS10635	hypothetical protein
Operon_1282	lilo_2053	LILO_RS10650	50S ribosomal protein L17
	lilo_2054	LILO_RS10655	DNA-directed RNA polymerase subunit alpha
	lilo_2055	LILO_RS10660	30S ribosomal protein S11
	lilo_2056	LILO_RS10665	30S ribosomal protein S13
	lilo_2057	LILO_RS10670	50S ribosomal protein L36
	lilo_2058	LILO_RS10675	translation initiation factor IF-1
Operon_1283	lilo_2059	LILO_RS10680	adenylate kinase
	lilo_2060	LILO_RS10685	preprotein translocase SecY subunit
Operon_1284	lilo_2061	LILO_RS10690	50S ribosomal protein L15
	lilo_2062	LILO_RS10695	50S ribosomal protein L30
	lilo_2063	LILO_RS10700	30S ribosomal protein S5
	lilo_2064	LILO_RS10705	50S ribosomal protein L18
Operon_1285	lilo_2065	LILO_RS10710	50S ribosomal protein L6
Operon_1286	lilo_2066	LILO_RS10715	30S ribosomal protein S8
Operon_1287	lilo_2067	LILO_RS10720	hypothetical protein
Operon_1288	lilo_2068	LILO_RS10725	30S ribosomal protein S14
	lilo_2069	LILO_RS10730	50S ribosomal protein L5
	lilo_2070	LILO_RS10735	50S ribosomal protein L24
Operon_1289	lilo_2071	LILO_RS10740	50S ribosomal protein L14
	lilo_2072	LILO_RS10745	30S ribosomal protein S17
	lilo_2073	LILO_RS10750	50S ribosomal protein L29
	lilo_2074	LILO_RS10755	50S ribosomal protein L16
	lilo_2075	LILO_RS10760	30S ribosomal protein S3
	lilo_2076	LILO_RS10765	50S ribosomal protein L22
	lilo_2077	LILO_RS10770	30S ribosomal protein S19
Operon_1290	lilo_2078	LILO_RS10775	50S ribosomal protein L2
	lilo_2079	LILO_RS10780	50S ribosomal protein L23

	lilo_2080	LILO_RS10785	50S ribosomal protein L4
	lilo_2081	LILO_RS10790	50S ribosomal protein L3
	lilo_2082	LILO_RS10795	30S ribosomal protein S10
Operon_1291	lilo_2083	LILO_RS10800	large conductance mechanosensitive channel protein
Operon_1292	lilo_2084	LILO_RS10805	hypothetical protein
	lilo_2085	LILO_RS10810	threonine synthase
Operon_1293	lilo_2086	LILO_RS10815	transcription antitermination protein
Operon_1294	lilo_2087	LILO_RS10820	preprotein translocase SecE subunit
Operon_1295	lilo_2088	LILO_RS10825	50S ribosomal protein L33
Operon_1296	lilo_2089	LILO_RS10830	hypothetical protein
Operon_1297	lilo_2090	LILO_RS10835	penicillin-binding protein 2a
Operon_1298	lilo_2091	LILO_RS10840	hypothetical protein
	lilo_2092	LILO_RS10845	hypothetical protein
Operon_1299	lilo_2093	LILO_RS10850	FMN-binding protein
Operon_1300	lilo_2094	LILO_RS10855	pseudouridine synthase
Operon_1301	lilo_2095	LILO_RS10860	ABC-type Mn2+/Zn2+ transport system, permease component
	lilo_2096	LILO_RS10865	zinc ABC transporter ATP binding protein
	lilo_2097	LILO_RS10870	zinc ABC transporter substrate binding protein
	lilo_2098	LILO_RS10875	zinc transport transcription regulator
Operon_1303	lilo_2099	LILO_RS10880	hypothetical protein
	lilo_2100	LILO_RS10885	putative competence protein
	lilo_2101	LILO_RS10890	hypothetical protein
	lilo_2102	LILO_RS10895	competence protein ComGD
	lilo_2103	lilo_2103	competence protein ComGC
	lilo_2104	LILO_RS10905	competence protein ComGB
	lilo_2105	LILO_RS10910	competence protein ComGA
Operon_1304	lilo_2106	LILO_RS10920	DNA polymerase III, alpha chain
Operon_1305	lilo_2107	LILO_RS10925	hypothetical protein
	lilo_2108	LILO_RS10930	NADH oxidase
	lilo_2109	LILO_RS10935	prolyl-tRNA synthetase
	lilo_2110	LILO_RS10940	hypothetical zinc metalloprotease
	lilo_2111	LILO_RS10945	phosphatidate cytidylyltransferase
	lilo_2112	LILO_RS10950	undecaprenyl pyrophosphate synthetase
Operon_1306	lilo_2113	LILO_RS10955	preprotein translocase YajC subunit
Operon_1307	lilo_2114	LILO_RS10960	hypothetical protein
	lilo_2115	LILO_RS10965	hypothetical protein
Operon_1308	lilo_2116	LILO_RS10970	exported serine protease
Operon_1309	lilo_2117	LILO_RS10975	glycosyltransferase
Operon_1310	lilo_2118	LILO_RS10980	lipopolysaccharide biosynthesis protein
Operon_1311	lilo_2119	LILO_RS10985	hypothetical protein
Operon_1312	lilo_2120	LILO_RS10990	hypothetical protein
Operon_1313	lilo_2121	LILO_RS10995	hypothetical protein
Operon_1314	lilo_2122	LILO_RS11000	DNA polymerase I
Operon_1315	lilo_2123	LILO_RS11010	transcriptional regulator
Operon_1316	lilo_2124	LILO_RS11035	competence regulator
Operon_1317	lilo_2125	LILO_RS11040	septation ring formation regulator

Operon_1318	lilo_2126	LILO_RS11045	elongation factor EF-Ts
Operon_1319	lilo_2127	LILO_RS11050	30S ribosomal protein S2
Operon_1320	lilo_2128	LILO_RS11055	3-carboxymuconate cyclase
Operon_1321	lilo_2129	LILO_RS11060	alcohol dehydrogenase / acetaldehyde dehydrogenase
Operon_1322	lilo_2130	LILO_RS11065	hypothetical protein
	lilo_2131	LILO_RS11070	hypothetical protein
	lilo_2132	LILO_RS11075	radical SAM protein
	lilo_2133	lilo_2133	radical SAM protein
Operon_1323	lilo_2134	LILO_RS11085	Rgg/GadR/MutR family transcriptional regulator
Operon_1324	lilo_2135	LILO_RS11090	PDZ domain-containing protein
	lilo_2136	LILO_RS11095	pantetheine-phosphate adenylyltransferase
	lilo_2137	LILO_RS11100	methyltransferase
Operon_1325	lilo_2138	LILO_RS11105	MF superfamily multidrug transporter
	lilo_2139	LILO_RS11110	MF superfamily multidrug transporter
Operon_1326	lilo_2140	LILO_RS11115	glucose-6-phosphate isomerase
Operon_1327	lilo_2141	LILO_RS11120	hypothetical protein
Operon_1328	lilo_2142	LILO_RS11125	hypothetical protein
Operon_1329	lilo_2143	LILO_RS11130	MarR family transcriptional regulator
Operon_1330	lilo_2144	LILO_RS11135	hypothetical protein
Operon_1331	lilo_2145	LILO_RS11140	valyl-tRNA synthetase
Operon_1332	lilo_2146	LILO_RS11145	fibronectin-binding protein
Operon_1333	lilo_2147	LILO_RS11150	hypothetical protein
	lilo_2148	LILO_RS11155	hypothetical protein
	lilo_2149	LILO_RS11160	GNAT family acetyltransferase
Operon_1334	lilo_2150	LILO_RS11165	tRNA-binding protein
Operon_1335	lilo_2151	LILO_RS11170	23S rRNA (uracil-5-)-methyltransferase RumA
Operon_1336	lilo_2152	LILO_RS11175	Mucus-binding protein, LPXTG-anchored
Operon_1337	lilo_2153	LILO_RS11180	regulatory protein recX
Operon_1338	lilo_2154	LILO_RS11205	gluconate transport protein
Operon_1339	lilo_2155	LILO_RS11210	gluconate kinase/ gluconokinase
	lilo_2156	LILO_RS11215	6-phosphogluconate dehydrogenase
Operon_1340	lilo_2157	LILO_RS11220	RpiR family transcriptional regulator
	lilo_2158	LILO_RS11225	hypothetical protein
Operon_1341	lilo_2159	LILO_RS11230	metallo-phosphoesterase
Operon_1342	lilo_2160	LILO_RS11235	30S ribosomal protein S18
Operon_1343	lilo_2161	LILO_RS11240	single-strand binding protein
	lilo_2162	LILO_RS11245	30S ribosomal protein S6
Operon_1344	lilo_2163	LILO_RS11250	undecaprenyl-diphosphatase
Operon_1345	lilo_2164	LILO_RS11255	hypothetical protein
Operon_1346	lilo_2165	LILO_RS11260	hypothetical protein
Operon_1347	lilo_2166	LILO_RS11265	lysine specific permease
Operon_1348	lilo_2167	LILO_RS11270	DNA polymerase III, subunits beta and tau
	lilo_2168	LILO_RS11275	aminoglycoside phosphotransferase
	lilo_2169	LILO_RS11280	GAF domain-containing protein
Operon_1349	lilo_2170	LILO_RS11285	putative chorismate mutase
Operon_1350	lilo_2171	LILO_RS11290	hypothetical protein

Operon_1351	lilo_2172	LILO_RS11295	hypothetical protein
Operon_1352	lilo_2173	LILO_RS11300	glutamine synthetase
	lilo_2174	LILO_RS11305	glutamine synthetase repressor/MerR family transcriptional regulator
Operon_1353	lilo_2175	LILO_RS11310	protein-tyrosine phosphatase
	lilo_2176	LILO_RS11315	ABC transporter ATP binding protein
	lilo_2177	LILO_RS11320	ABC transporter permease protein
Operon_1354	lilo_2178	LILO_RS11325	hypothetical protein
Operon_1355	lilo_2179	LILO_RS11330	Rgg/GadR/MutR family transcriptional regulator
Operon_1356	lilo_2180	LILO_RS11335	Holliday junction DNA helicase B
Operon_1357	lilo_2181	LILO_RS11340	DNA helicase RuvA
	lilo_2182	LILO_RS11345	DNA mismatch repair protein MutL
Operon_1358	lilo_2183	LILO_RS11350	hypothetical protein
Operon_1359	lilo_2184	LILO_RS11355	mismatch repair protein MutS
Operon_1360	lilo_2185	LILO_RS11360	hypothetical protein
Operon_1361	lilo_2186	LILO_RS11365	3-hydroxyisobutyrate dehydrogenase
	lilo_2187	lilo_2187	hypothetical protein
Operon_1362	lilo_2188	LILO_RS11385	permease
Operon_1363	lilo_2189	LILO_RS11390	hypothetical protein
Operon_1364	lilo_2190	LILO_RS11395	glucose-6-phosphate 1-dehydrogenase
Operon_1365	lilo_2191	LILO_RS11400	HAD superfamily hydrolase
Operon_1366	lilo_2192	LILO_RS11405	DnaJ protein
Operon_1367	lilo_2193	LILO_RS11410	hypothetical protein
	lilo_2194	LILO_RS11415	aspartate racemase
	lilo_2195	LILO_RS11420	ATP-binding protein
	lilo_2196	LILO_RS11425	asparagine synthetase
Operon_1368	lilo_2197	LILO_RS11430	hypothetical protein
Operon_1369	lilo_2198	LILO_RS11435	rod shape-determining protein MreD
	lilo_2199	LILO_RS11440	rod shape-determining protein MreC
Operon_1371	lilo_2200	LILO_RS11445	MutT/nudix family hydrolase
Operon_1372	lilo_2201	LILO_RS11450	ribose 5-phosphate isomerase A
Operon_1373	lilo_2202	LILO_RS11455	Crp family transcriptional regulator
Operon_1374	lilo_2203	LILO_RS11460	MFS superfamily transporter
	lilo_2204	LILO_RS11465	universal stress protein A
	lilo_2205	LILO_RS11470	hypothetical protein
Operon_1375	lilo_2206	LILO_RS11475	activator of (R)-2-hydroxyglutaryl-CoA dehydratase
Operon_1379	lilo_2207	LILO_RS11480	transcription regulator
Operon_1380	lilo_2209	LILO_RS11490	hypothetical protein
	lilo_2208	LILO_RS11485	ribosomal small subunit pseudouridine synthase A
Operon_1381	lilo_2210	LILO_RS11495	GTP-binding protein ThdF
Operon_1382	lilo_2212	LILO_RS11505	ATP-dependent DNA helicase
	lilo_2211	LILO_RS11500	hypothetical protein
Operon_1383	lilo_2213	LILO_RS11510	alkylphosphonate uptake protein
Operon_1384	lilo_2214	LILO_RS11515	glyceraldehyde 3-phosphate dehydrogenase
Operon_1385	lilo_2215	LILO_RS11525	hypothetical protein
Operon_1386	lilo_2216	LILO_RS11530	cation-transporting ATPase
Operon_1387	lilo_2217	LILO_RS11535	transcriptional regulator

Operon_1388	lilo_2218	LILO_RS11540	oxidoreductase
Operon_1389	lilo_2219	LILO_RS11545	transporter
Operon_1390	lilo_2221	LILO_RS11605	50S ribosomal protein L13
	lilo_2220	LILO_RS11600	30S ribosomal protein S9
Operon_1391	lilo_2222	LILO_RS11610	hypothetical protein
	lilo_2223	LILO_RS11615	ABC transporter ATP-binding protein
Operon_1392	lilo_2224	LILO_RS11620	ribonuclease HIII
	lilo_2225	LILO_RS11625	signal peptidase I
Operon_1393	lilo_2226	LILO_RS11630	regulator of purine biosynthetic genes
Operon_1394	lilo_2227	LILO_RS11635	elongation factor EF-G
Operon_1395	lilo_2229	LILO_RS11645	30S ribosomal protein S12
	lilo_2228	LILO_RS11640	30S ribosomal protein S7
Operon_1396	lilo_2230	LILO_RS11650	D-alanyl-D-alanine carboxypeptidase
Operon_1397	lilo_2231	LILO_RS11655	transporter
Operon_1398	lilo_2232	LILO_RS11660	hypothetical protein
Operon_1399	lilo_2233	LILO_RS11665	hypothetical protein

Table S3. *L. lactis* IO-1 Regulon list.

Predicted TF	RegulonID	# members	Members
	**Regulon_1**	6	Operon_96; Operon_131; Operon_584; Operon_741; Operon_1038; Operon_1047
	**Regulon_2**	8	Operon_123; Operon_125; Operon_197; Operon_259; Operon_860; Operon_883; Operon_920; Operon_1277
	**Regulon_3**	19	Operon_4; Operon_131; Operon_101; Operon_126; Operon_187; Operon_213; Operon_303; Operon_312; Operon_373; Operon_421; Operon_541; Operon_557; Operon_617; Operon_650; Operon_873; Operon_939; Operon_1250; Operon_1301; Operon_1375
	**Regulon_4**	11	Operon_24; Operon_1375; Operon_131; Operon_92; Operon_127; Operon_619; Operon_1215; Operon_1217; Operon_1322; Operon_1332; Operon_1333
	**Regulon_5**	2	Operon_130; Operon_340
	**Regulon_6**	12	Operon_144; Operon_151; Operon_170; Operon_367; Operon_410; Operon_562; Operon_573; Operon_575; Operon_620; Operon_866; Operon_1010; Operon_1043
	**Regulon_7**	5	Operon_254; Operon_403; Operon_474; Operon_578; Operon_1187
	**Regulon_8**	11	Operon_218; Operon_584; Operon_225; Operon_528; Operon_874; Operon_900; Operon_905; Operon_1141; Operon_1238; Operon_1239; Operon_1361
	**Regulon_9**	10	Operon_9; Operon_131; Operon_186; Operon_192; Operon_419; Operon_537; Operon_582; Operon_933; Operon_1071; Operon_1176
**XylR**	**Regulon_10**	3	Operon_10; Operon_877;Operon_1401;Operon_876
	**Regulon_11**	3	Operon_400; Operon_612; Operon_624
	**Regulon_12**	3	Operon_603; Operon_606; Operon_1352
	**Regulon_13**	14	Operon_88; Operon_620; Operon_116; Operon_214; Operon_220; Operon_261; Operon_321; Operon_395; Operon_413; Operon_549; Operon_937; Operon_1132; Operon_1281; Operon_1295
	**Regulon_14**	2	Operon_221; Operon_1037
	**Regulon_15**	13	Operon_94; Operon_164; Operon_236; Operon_263; Operon_326; Operon_443; Operon_735; Operon_799; Operon_876; Operon_1075; Operon_1273; Operon_1279; Operon_1299
	**Regulon_16**	3	Operon_768; Operon_1017; Operon_1065
	**Regulon_17**	3	Operon_328; Operon_768; Operon_764
	**Regulon_18**	4	Operon_1157; Operon_1212; Operon_1293; Operon_1360
	**Regulon_19**	3	Operon_266; Operon_436; Operon_1026
	**Regulon_20**	2	Operon_351; Operon_1021
	**Regulon_21**	9	Operon_129; Operon_420; Operon_434; Operon_635; Operon_637; Operon_649; Operon_849; Operon_1048; Operon_1204
	**Regulon_22**	3	Operon_447; Operon_1021; Operon_459
	**Regulon_23**	3	Operon_260; Operon_437; Operon_1030
	**Regulon_24**	32	Operon_5; Operon_420; Operon_84; Operon_1030; Operon_95; Operon_119; Operon_154; Operon_161; Operon_178; Operon_200; Operon_204; Operon_278; Operon_338; Operon_431; Operon_458; Operon_480; Operon_484; Operon_503; Operon_634; Operon_645; Operon_755; Operon_904; Operon_909; Operon_1040;

			Operon_1127; Operon_1143; Operon_1160; Operon_1163; Operon_1182; Operon_1190; Operon_1196; Operon_1205
	**Regulon_25**	4	Operon_294; Operon_439; Operon_571; Operon_687
	**Regulon_26**	3	Operon_808; Operon_924; Operon_928
	**Regulon_27**	6	Operon_201; Operon_321; Operon_1233; Operon_431; Operon_1327; Operon_131
	**Regulon_28**	3	Operon_432; Operon_910; Operon_1189
	**Regulon_29**	8	Operon_270; Operon_350; Operon_440; Operon_502; Operon_506; Operon_875; Operon_925; Operon_1016
	**Regulon_30**	4	Operon_628; Operon_278; Operon_1150; Operon_1229
	**Regulon_31**	3	Operon_587; Operon_618; Operon_1272
	**Regulon_32**	6	Operon_539; Operon_628; Operon_1014; Operon_1272; Operon_1064; Operon_1313
	**Regulon_33**	4	Operon_43; Operon_632; Operon_971; Operon_1219
	**Regulon_34**	7	Operon_22; Operon_618; Operon_608; Operon_629; Operon_989; Operon_1165; Operon_1167
	**Regulon_35**	3	Operon_515; Operon_630; Operon_818
	**Regulon_36**	3	Operon_533; Operon_837; Operon_1115
	**Regulon_37**	3	Operon_319; Operon_993; Operon_1393
	**Regulon_38**	3	Operon_315; Operon_993; Operon_566
	**Regulon_39**	5	Operon_318; Operon_791; Operon_995; Operon_1061; Operon_1103
	**Regulon_40**	4	Operon_314; Operon_458; Operon_940; Operon_1169
	**Regulon_41**	4	Operon_206; Operon_88; Operon_317; Operon_1221
	**Regulon_42**	3	Operon_190; Operon_999; Operon_1098
	**Regulon_43**	2	Operon_591; Operon_908
	**Regulon_44**	6	Operon_27; Operon_97; Operon_761; Operon_1151; Operon_1193; Operon_1321
	**Regulon_45**	2	Operon_1303; Operon_1381
	**Regulon_46**	2	Operon_482; Operon_1193
	**Regulon_47**	4	Operon_182; Operon_185; Operon_1095; Operon_1206
	**Regulon_48**	4	Operon_180; Operon_918; Operon_1094; Operon_1305
	**Regulon_49**	3	Operon_177; Operon_746; Operon_1191
	**Regulon_50**	2	Operon_759; Operon_1349
	**Regulon_51**	3	Operon_850; Operon_1191; Operon_1268
**BusR**	**Regulon_52**	4	Operon_830; Operon_661; Operon_848
	**Regulon_53**	3	Operon_344; Operon_1144; Operon_1347
	**Regulon_54**	4	Operon_653; Operon_1007; Operon_1320; Operon_1357
	**Regulon_55**	3	Operon_342; Operon_590; Operon_1013
	**Regulon_56**	3	Operon_153; Operon_679; Operon_942
	**Regulon_57**	4	Operon_85; Operon_1014; Operon_583; Operon_1009
	**Regulon_58**	2	Operon_155; Operon_1307
	**Regulon_59**	4	Operon_159; Operon_406; Operon_453; Operon_1137
	**Regulon_60**	7	Operon_182; Operon_211; Operon_217; Operon_407; Operon_816; Operon_1254; Operon_1296
	**Regulon_61**	4	Operon_167; Operon_554; Operon_740; Operon_1344
	**Regulon_62**	3	Operon_273; Operon_1154; Operon_1257
	**Regulon_63**	2	Operon_65; Operon_1342
	**Regulon_64**	3	Operon_840; Operon_1143; Operon_1342
	**Regulon_65**	3	Operon_227; Operon_905; Operon_1164

	**Regulon_66**	7	Operon_192; Operon_453; Operon_279; Operon_643; Operon_926; Operon_1138; Operon_1263
	**Regulon_67**	5	Operon_274; Operon_659; Operon_1146; Operon_1155
	**Regulon_68**	4	Operon_592; Operon_926; Operon_827; Operon_1178
	**Regulon_69**	4	Operon_460; Operon_561; Operon_884; Operon_1096
	**Regulon_70**	3	Operon_734; Operon_460; Operon_1052
	**Regulon_71**	2	Operon_972; Operon_350
	**Regulon_72**	5	Operon_76; Operon_99; Operon_257; Operon_970; Operon_1324
	**Regulon_73**	3	Operon_46; Operon_883; Operon_969
	**Regulon_74**	3	Operon_75; Operon_257; Operon_369
	**Regulon_75**	2	Operon_415; Operon_828; Operon_1340
	**Regulon_76**	3	Operon_272; Operon_823; Operon_1090
	**Regulon_77**	4	Operon_176; Operon_828; Operon_531; Operon_822
	**Regulon_78**	4	Operon_389; Operon_692; Operon_753; Operon_1086
	**Regulon_79**	4	Operon_343; Operon_365; Operon_747; Operon_1218
	**Regulon_80**	3	Operon_752; Operon_1230
	**Regulon_81**	4	Operon_462; Operon_532; Operon_1228; Operon_1336
	**Regulon_82**	3	Operon_277; Operon_405; Operon_1359
	**Regulon_83**	4	Operon_51; Operon_541; Operon_404; Operon_912
	**Regulon_84**	3	Operon_552; Operon_389; Operon_1213
	**Regulon_85**	4	Operon_402; Operon_550; Operon_551; Operon_756
	**Regulon_86**	4	Operon_267; Operon_335; Operon_392; Operon_401
	**Regulon_87**	3	Operon_62; Operon_337; Operon_1227
	**Regulon_88**	3	Operon_1100; Operon_307; Operon_312
	**Regulon_89**	3	Operon_48; Operon_322; Operon_324
	**Regulon_90**	4	Operon_327; Operon_836; Operon_961; Operon_1252
**CodY**	**Regulon_91**	15	Operon_1159;Operon_1184;Operon_192;Operon_316;Operon_417;Operon_661;Operon_682;Operon_686;Operon_757;Operon_83;Operon_939;Operon_961;Operon_761;Operon_1191; Operon_350
	**Regulon_92**	14	Operon_56; Operon_190; Operon_491; Operon_497; Operon_1261; Operon_937;Operon_197;Operon_584;Operon_33;Operon_3; Operon_261;Operon_653;Operon_1083;Operon_1085
	**Regulon_93**	4	Operon_501; Operon_259; Operon_538; Operon_1211
	**Regulon_94**	3	Operon_438; Operon_601; Operon_1003
	**Regulon_95**	3	Operon_449; Operon_689; Operon_998
	**Regulon_96**	5	Operon_269; Operon_419; Operon_397; Operon_1182; Operon_1259
	**Regulon_97**	2	Operon_861; Operon_1161
	**Regulon_98**	4	Operon_276; Operon_284; Operon_856; Operon_862
	**Regulon_99**	4	Operon_268; Operon_1254; Operon_858; Operon_867
	**Regulon_100**	3	Operon_1055; Operon_539; Operon_1253
	**Regulon_101**	4	Operon_379; Operon_75; Operon_677; Operon_629
	**Regulon_102**	6	Operon_231; Operon_380; Operon_656; Operon_682; Operon_810; Operon_811
	**Regulon_103**	5	Operon_8; Operon_158; Operon_376; Operon_625; Operon_731
	**Regulon_104**	2	Operon_293; Operon_1367

	**Regulon_105**	1	Operon_1085
	**Regulon_106**	3	Operon_399; Operon_8; Operon_1315
	**Regulon_107**	2	Operon_463; Operon_663
	**Regulon_108**	3	Operon_282; Operon_662; Operon_1201
	**Regulon_109**	4	Operon_90; Operon_661; Operon_553; Operon_1341
	**Regulon_110**	4	Operon_23; Operon_1239; Operon_558; Operon_1092
	**Regulon_111**	2	Operon_194; Operon_557
	**Regulon_112**	4	Operon_203; Operon_553; Operon_518; Operon_1104
	**Regulon_113**	4	Operon_210; Operon_698; Operon_777; Operon_1074
	**Regulon_114**	4	Operon_17; Operon_33; Operon_202; Operon_425
	**Regulon_115**	4	Operon_25; Operon_320; Operon_455; Operon_479
	**Regulon_116**	3	Operon_77; Operon_633; Operon_1166
	**Regulon_117**	3	Operon_483; Operon_631; Operon_1368
	**Regulon_118**	3	Operon_193; Operon_488; Operon_640
	**Regulon_119**	4	Operon_744; Operon_213; Operon_780; Operon_988
	**Regulon_120**	3	Operon_165; Operon_211; Operon_855
	**Regulon_121**	3	Operon_1162; Operon_1296; Operon_1325
	**Regulon_122**	10	Operon_508; Operon_400; Operon_685; Operon_617; Operon_977; Operon_1060; Operon_1148; Operon_1288; Operon_1290; Operon_1304
	**Regulon_123**	4	Operon_207; Operon_753; Operon_843; Operon_1245
	**Regulon_124**	7	Operon_235; Operon_419; Operon_159; Operon_464; Operon_1229; Operon_841; Operon_1297
	**Regulon_125**	4	Operon_611; Operon_235; Operon_646; Operon_1350
	**Regulon_126**	5	Operon_681; Operon_969; Operon_46; Operon_1280; Operon_611
	**Regulon_127**	5	Operon_280; Operon_273; Operon_920; Operon_512; Operon_279
	**Regulon_128**	3	Operon_78; Operon_168; Operon_1174
	**Regulon_129**	3	Operon_40; Operon_285; Operon_913
	**Regulon_130**	4	Operon_32; Operon_104; Operon_253; Operon_1309
	**Regulon_131**	3	Operon_368; Operon_1293; Operon_1184
	**Regulon_132**	3	Operon_37; Operon_43; Operon_915
	**Regulon_133**	3	Operon_35; Operon_438; Operon_38
	**Regulon_134**	3	Operon_36; Operon_287; Operon_1306
	**Regulon_135**	7	Operon_348; Operon_611; Operon_35; Operon_615; Operon_650; Operon_1343; Operon_1245
	**Regulon_136**	2	Operon_798; Operon_192
**PerR**	**Regulon_137**	1	Operon_858
	**Regulon_138**	3	Operon_1369; Operon_207; Operon_192
	**Regulon_139**	5	Operon_199; Operon_223; Operon_877; Operon_927; Operon_1241
	**Regulon_140**	4	Operon_1004; Operon_199; Operon_204; Operon_1164
	**Regulon_141**	3	Operon_68; Operon_230; Operon_1339
	**Regulon_142**	2	Operon_795; Operon_1062
	**Regulon_143**	3	Operon_329; Operon_1341; Operon_1379
	**Regulon_144**	3	Operon_10; Operon_802; Operon_1068
	**Regulon_145**	3	Operon_616; Operon_802; Operon_674
	**Regulon_146**	3	Operon_375; Operon_293; Operon_683

	**Regulon_147**	4	Operon_150; Operon_921; Operon_932; Operon_1214
	**Regulon_148**	3	Operon_147; Operon_1357; Operon_1002
	**Regulon_149**	4	Operon_808; Operon_1151; Operon_931; Operon_1120
	**Regulon_150**	2	Operon_930; Operon_1149
	**Regulon_151**	3	Operon_1039; Operon_1089; Operon_1188
	**Regulon_152**	3	Operon_57; Operon_1148; Operon_387
	**Regulon_153**	4	Operon_1242; Operon_677; Operon_1329; Operon_1089
	**Regulon_154**	3	Operon_758; Operon_1025; Operon_1153
**BirA**	**Regulon_155**	3	Operon_1088;Operon_1087
	**Regulon_156**	4	Operon_585; Operon_613; Operon_960; Operon_1059
	**Regulon_157**	4	Operon_79; Operon_95; Operon_498; Operon_959
	**Regulon_158**	4	Operon_228; Operon_959; Operon_964; Operon_1275
	**Regulon_159**	7	Operon_114; Operon_964; Operon_173; Operon_1176; Operon_247; Operon_648; Operon_1314
	**Regulon_160**	3	Operon_243; Operon_78; Operon_412
	**Regulon_161**	4	Operon_429; Operon_253; Operon_639; Operon_254
	**Regulon_162**	5	Operon_83; Operon_1348; Operon_86; Operon_195; Operon_954
	**Regulon_163**	6	Operon_7; Operon_83; Operon_336; Operon_569; Operon_1057; Operon_1292
	**Regulon_164**	3	Operon_87; Operon_791; Operon_346
	**Regulon_165**	5	Operon_21; Operon_657; Operon_757; Operon_786; Operon_1237
	**Regulon_166**	3	Operon_516; Operon_722; Operon_779
	**Regulon_167**	2	Operon_437; Operon_774
	**Regulon_168**	3	Operon_59; Operon_193; Operon_872
	**Regulon_169**	3	Operon_919; Operon_329; Operon_758
	**Regulon_170**	4	Operon_626; Operon_755; Operon_1271; Operon_1167
	**Regulon_171**	3	Operon_535; Operon_4; Operon_589
	**Regulon_172**	3	Operon_529; Operon_530; Operon_842
	**Regulon_173**	2	Operon_1323; Operon_549
	**Regulon_174**	3	Operon_157; Operon_518; Operon_896
	**Regulon_175**	4	Operon_208; Operon_517; Operon_1287; Operon_1316
	**Regulon_176**	4	Operon_289; Operon_302; Operon_349; Operon_433
	**Regulon_177**	3	Operon_81; Operon_338; Operon_430
	**Regulon_178**	2	Operon_341
	**Regulon_179**	4	Operon_205; Operon_344; Operon_806; Operon_847
	**Regulon_180**	2	Operon_140; Operon_820
	**Regulon_181**	2	Operon_853; Operon_585
	**Regulon_182**	3	Operon_762; Operon_868; Operon_1121
	**Regulon_183**	3	Operon_391; Operon_294; Operon_648
	**Regulon_184**	2	Operon_118; Operon_908
	**Regulon_186**	1	Operon_841
	**Regulon_187**	1	Operon_135
	**Regulon_188**	1	Operon_906
	**Regulon_189**	1	Operon_29
	**Regulon_190**	1	Operon_1218
	**Regulon_191**	2	Operon_424; Operon_944

	**Regulon_192**	1	Operon_464
	**Regulon_193**	2	Operon_115; Operon_862
	**Regulon_194**	1	Operon_255
	**Regulon_195**	1	Operon_960
	**Regulon_196**	2	Operon_584; Operon_514
	**Regulon_197**	2	Operon_3; Operon_739
	**Regulon_198**	3	Operon_245; Operon_1196; Operon_660
	**Regulon_199**	1	Operon_885
	**Regulon_200**	2	Operon_232; Operon_25
	**Regulon_201**	2	Operon_306; Operon_408
	**Regulon_202**	1	Operon_805
	**Regulon_203**	1	Operon_1282
	**Regulon_204**	1	Operon_1374
	**Regulon_205**	2	Operon_577; Operon_654
	**Regulon_206**	1	Operon_265
	**Regulon_207**	2	Operon_39; Operon_1135
	**Regulon_208**	2	Operon_1131; Operon_843
	**Regulon_209**	1	Operon_645
	**Regulon_210**	2	Operon_107; Operon_485
	**Regulon_211**	3	Operon_240; Operon_1359; Operon_617
**AdcR**	**Regulon_212**	2	Operon_529; Operon_1301
	**Regulon_213**	3	Operon_152; Operon_962; Operon_1027
	**Regulon_214**	1	Operon_1129
	**Regulon_215**	2	Operon_339; Operon_1140
	**Regulon_216**	1	Operon_1181
	**Regulon_217**	6	Operon_44; Operon_392; Operon_1154; Operon_1175; Operon_432; Operon_1324
	**Regulon_218**	4	Operon_171; Operon_1102; Operon_1278; Operon_1338
	**Regulon_219**	4	Operon_52; Operon_108; Operon_887; Operon_1298
	**Regulon_220**	4	Operon_559; Operon_998; Operon_567; Operon_1026
	**Regulon_221**	3	Operon_295; Operon_1280; Operon_781
	**Regulon_222**	3	Operon_139; Operon_1057; Operon_461
	**Regulon_223**	4	Operon_137; Operon_322; Operon_473; Operon_1369
	**Regulon_224**	4	Operon_142; Operon_79; Operon_435; Operon_1156
	**Regulon_225**	2	Operon_623; Operon_1314
	**Regulon_226**	4	Operon_149; Operon_144; Operon_333; Operon_1024
	**Regulon_227**	3	Operon_138; Operon_737; Operon_898
	**Regulon_228**	4	Operon_509; Operon_1106; Operon_1395
	**Regulon_229**	2	Operon_120; Operon_1395
	**Regulon_230**	3	Operon_106; Operon_1369; Operon_132
	**Regulon_231**	7	Operon_112; Operon_106; Operon_588; Operon_1261; Operon_651; Operon_1268; Operon_1069
	**Regulon_232**	3	Operon_15; Operon_118; Operon_103
	**Regulon_233**	4	Operon_398; Operon_1175; Operon_1168; Operon_1184
	**Regulon_234**	3	Operon_237; Operon_1169; Operon_316
	**Regulon_235**	2	Operon_1; Operon_933
	**Regulon_236**	3	Operon_229; Operon_1203; Operon_1222

	**Regulon_237**	4	Operon_670; Operon_237; Operon_936; Operon_1317
	**Regulon_238**	4	Operon_100; Operon_99; Operon_417; Operon_1228
	**Regulon_239**	3	Operon_446; Operon_97; Operon_1334
	**Regulon_240**	4	Operon_234; Operon_112; Operon_473; Operon_563; Operon_687
	**Regulon_241**	2	Operon_347; Operon_951
	**Regulon_242**	3	Operon_2; Operon_644; Operon_1018
	**Regulon_243**	3	Operon_355; Operon_1095
	**Regulon_244**	4	Operon_67; Operon_927; Operon_353; Operon_1099
	**Regulon_245**	3	Operon_64; Operon_312; Operon_911
	**Regulon_246**	3	Operon_356; Operon_657; Operon_449
	**Regulon_247**	3	Operon_776; Operon_211; Operon_1045
	**Regulon_248**	4	Operon_815; Operon_1327; Operon_1045; Operon_861
	**Regulon_249**	3	Operon_767; Operon_369; Operon_792
	**Regulon_250**	2	Operon_360; Operon_451
	**Regulon_251**	3	Operon_1331; Operon_904; Operon_451
	**Regulon_252**	5	Operon_647; Operon_644; Operon_652; Operon_1237; Operon_1082
	**Regulon_253**	3	Operon_973; Operon_646; Operon_1128
	**Regulon_254**	2	Operon_418; Operon_1353
	**Regulon_255**	3	Operon_903; Operon_1132; Operon_1366
	**Regulon_256**	5	Operon_923; Operon_1069; Operon_81; Operon_1008; Operon_1201
	**Regulon_257**	2	Operon_1130; Operon_1127
	**Regulon_258**	2	Operon_1067; Operon_903
	**Regulon_259**	4	Operon_311; Operon_170; Operon_1036; Operon_550
	**Regulon_260**	3	Operon_565; Operon_973; Operon_1022
	**Regulon_261**	3	Operon_41; Operon_142; Operon_169
	**Regulon_262**	4	Operon_172; Operon_820; Operon_313; Operon_1135
	**Regulon_263**	4	Operon_1042; Operon_501; Operon_395; Operon_149
	**Regulon_265**	4	Operon_382; Operon_488; Operon_500; Operon_1029
	**Regulon_264**	1	Operon_174
	**Regulon_266**	3	Operon_1033; Operon_850; Operon_174
	**Regulon_267**	5	Operon_496; Operon_1029; Operon_1033; Operon_1174; Operon_1343
	**Regulon_268**	3	Operon_422; Operon_672; Operon_809
	**Regulon_269**	4	Operon_870; Operon_1204; Operon_672; Operon_349
	**Regulon_270**	3	Operon_416; Operon_1106; Operon_168
	**Regulon_271**	3	Operon_790; Operon_165; Operon_1195
	**Regulon_272**	3	Operon_505; Operon_178; Operon_1243
	**Regulon_273**	5	Operon_45; Operon_1039; Operon_50; Operon_1150; Operon_690
	**Regulon_274**	2	Operon_824; Operon_1356
	**Regulon_275**	3	Operon_426; Operon_415; Operon_39
	**Regulon_276**	5	Operon_345; Operon_913; Operon_1195; Operon_1300; Operon_1060
	**Regulon_277**	4	Operon_55; Operon_1180; Operon_1194; Operon_1269
	**Regulon_278**	2	Operon_978; Operon_1123
	**Regulon_279**	3	Operon_1028; Operon_698; Operon_1047

	**Regulon_280**	4	Operon_1192; Operon_38; Operon_1065; Operon_1336
	**Regulon_281**	4	Operon_42; Operon_921; Operon_1128; Operon_1130
	**Regulon_282**	3	Operon_487; Operon_912; Operon_489
	**Regulon_283**	3	Operon_54; Operon_216; Operon_917
	**Regulon_284**	3	Operon_55; Operon_509; Operon_978
	**Regulon_285**	6	Operon_283; Operon_149; Operon_690; Operon_393; Operon_1301; Operon_813
	**Regulon_286**	3	Operon_309; Operon_514; Operon_1353
	**Regulon_287**	5	Operon_1000; Operon_309; Operon_1109; Operon_1249; Operon_1337
	**Regulon_288**	4	Operon_181; Operon_292; Operon_467; Operon_817
	**Regulon_289**	2	Operon_1362; Operon_1109
	**Regulon_290**	2	Operon_495; Operon_1288
	**Regulon_291**	3	Operon_543; Operon_500; Operon_909
	**Regulon_292**	3	Operon_494; Operon_543; Operon_199
	**Regulon_293**	1	Operon_777
	**Regulon_294**	4	Operon_359; Operon_528; Operon_1280; Operon_1073
	**Regulon_295**	3	Operon_277; Operon_1229; Operon_284
	**Regulon_296**	5	Operon_297; Operon_661; Operon_477; Operon_880; Operon_1041
**MleR**	**Regulon_297**	1	Operon_536
	**Regulon_298**	5	Operon_465; Operon_1003; Operon_940; Operon_947; Operon_1283
	**Regulon_299**	3	Operon_950; Operon_533; Operon_842
	**Regulon_300**	3	Operon_510; Operon_240; Operon_1104
	**Regulon_301**	3	Operon_621;Operon_1289;Operon_586
	**Regulon_302**	4	Operon_98;Operon_1105;Operon_1396;Operon_773
	**Regulon_326**	2	Operon_362;Operon_586
	**Regulon_305**	4	Operon_362;Operon_364;Operon_745;Operon_608
	**Regulon_306**	5	Operon_1254;Operon_362;Operon_497;Operon_366;Operon_32
	**Regulon_307**	4	Operon_29;Operon_686;Operon_1201;Operon_362
**ArgR**	**Regulon_308**	5	Operon_1259;Operon_1093;Operon_457;Operon_53;Operon_1254
**CcpA**	**Regulon_309**	30	Operon_133;Operon_1006;Operon_1378;Operon_807;Operon_964;Operon_1043;Operon_1241;Operon_1242;Operon_1220;Operon_11;Operon_842;Operon_187;Operon_1014;Operon_1023;Operon_1176;Operon_573;Operon_1005;Operon_1030;Operon_342;Operon_1320;Operon_1102;Operon_998;Operon_347;Operon_1326;Operon_394;Nan;Operon_853;Operon_1080;Operon_568;Operon_533;Operon_870
**CmbR**	**Regulon_310**	10	Operon_29;Operon_297;Operon_555;Operon_180;Operon_1271;Operon_443;Operon_276;Operon_742;Operon_1184;Operon_735
**CopR**	**Regulon_312**	5	Operon_1228;Operon_494;Operon_32;Operon_475;Operon_216
**FabT**	**Regulon_313**	4	Operon_438;Operon_293;Operon_294;Operon_683
**GlnR**	**Regulon_314**	3	Operon_1352;Operon_939;Operon_1075
**HomR**	**Regulon_315**	2	Operon_742;Operon_443
**HrcA**	**Regulon_316**	4	Operon_1366;Operon_566;Operon_227;Operon_1204
**MntR**	**Regulon_317**	2	Operon_794;Operon_743

**MtlR**	**Regulon_319**	1	Operon_10
**NagR**	**Regulon_320**	4	Operon_928;Operon_591;Operon_808; Operon_924
**NrdR**	**Regulon_321**	2	Operon_578;Operon_145
**MntR**	**Regulon_322**	3	Operon_858;Operon_1301;Operon_743
**PurR**	**Regulon_323**	16	Operon_553;Operon_1196;Operon_495;Operon_314;Operon_884;Operon_840;Operon_889;Operon_900;Operon_878;Operon_1377;Operon_653;Operon_899;Operon_1061;Operon_885;Operon_1393;Operon_1394
**Rex**	**Regulon_324**	7	Operon_601;Operon_126;Operon_1176;Operon_806;Operon_1321;Operon_343;Operon_147
**YthA**	**Regulon_325**	10	Operon_608;Operon_947;Operon_53;Operon_292;Operon_289;Operon_1075;Operon_1144;Operon_364;Operon_366;Operon_586

Table S4. Detail information on *L. lactis* IO-1 predicted Regulons.

**Regulon_92**			
Operon_56	lilo_0091	LILO_RS00515	ribonuclease P protein component
	lilo_0092	LILO_RS00520	preprotein translocase subunit YidC
Operon_190	lilo_0307	LILO_RS01640	RecM protein
	lilo_0308	LILO_RS01645	D-alanyl-alanine synthetase A
	lilo_0309	LILO_RS01650	UDP-N-acetylmuramoylalanyl-D-glutamyl-2,6-diaminopimelate--D-alanyl-D-alanine ligase
Operon_491	lilo_0784	LILO_RS04155	hypothetical protein
Operon_497	lilo_0792	LILO_RS04195	glycerate kinase
	lilo_0793	LILO_RS04200	geranyltranstransferase/ dimethylallyltransferase
	lilo_0794	LILO_RS04205	rRNA methylase
	lilo_0795	LILO_RS04210	transcriptional repressor, arginine deiminase pathway
Operon_1261	lilo_2029	LILO_RS10520	UDP-N-acetylmuramate-alanine ligase
Operon_937	lilo_1512	LILO_RS07810	cell division protein FtsQ
	lilo_1513	LILO_RS07815	UDP-N-acetylglucosamine--N-acetylmuramyl-(pentapeptide) pyrophosphoryl-undecaprenol N-acetylglucosamine transferase
	lilo_1514	LILO_RS07820	UDP-N-acetylmuramoylalanine D-glutamate ligase
Operon_197	lilo_0320	LILO_RS01705	formamidopyrimidine-DNA glycosylase
Operon_584	lilo_0932	LILO_RS04890	topoisomerase IV subunit B
Operon_33	lilo_0054	LILO_RS00315	50S ribosomal protein L33
	lilo_0055	LILO_RS00320	50S ribosomal protein L32
Operon_3	lilo_0003	LILO_RS00020	ATP-dependent nuclease subunit B
	lilo_0004	LILO_RS00025	ATP-dependent nuclease subunit A
	lilo_0005	LILO_RS00030	hypothetical protein
Operon_261	lilo_0401	LILO_RS02125	tRNA pseudouridine synthase A
	lilo_0402	LILO_RS02130	phosphomethylpyrimidine kinase
	lilo_0403	LILO_RS02135	hypothetical protein
	lilo_0404	LILO_RS02140	hypothetical protein
	lilo_0405	LILO_RS02145	serine--pyruvate aminotransferase
Operon_653	lilo_1046	LILO_RS05450	GMP reductase
Operon_1083	lilo_1754	LILO_RS09075	competence protein ComEC
	lilo_1755	LILO_RS09080	competence protein ComEA
	lilo_1756	LILO_RS09085	ABC transporter permease protein
	lilo_1757	LILO_RS09090	sodium transport system ATP-binding protein
Operon_1085	lilo_1759	LILO_RS09100	acyltransferase
**Regulon_93**			
Operon_501	lilo_0799	LILO_RS04230	transporter
	lilo_0800	LILO_RS04235	hypothetical protein
Operon_259	lilo_0398	LILO_RS02105	ATP/GTP hydrolase
	lilo_0399	LILO_RS02110	GNAT family acetyltransferase
Operon_538	lilo_0854	LILO_RS04495	hypothetical protein
	lilo_0855	LILO_RS04500	HAD superfamily hydrolase
Operon_1211	lilo_1960	LILO_RS10165	CDP-diacylglycerol-phosphate phosphatidyltransferase

	lilo_1961	LILO_RS10170	hypothetical protein
**Regulon_96**			
Operon_269	lilo_0418	LILO_RS02215	HU-like DNA-binding protein
Operon_419	lilo_0674	LILO_RS03605	signaling protein
	lilo_0675	LILO_RS03610	50S ribosomal protein L9
	lilo_0676	LILO_RS03615	replicative DNA helicase
Operon_397	lilo_0637	LILO_RS03430	cytochrome bd-I oxidase subunit I
	lilo_0638	LILO_RS03435	cytochrome d ubiquinol oxidase subunit II
Operon_1182	lilo_1920	LILO_RS09955	Upp protein
Operon_1259	lilo_2027	LILO_RS10510	arginyl-tRNA synthetase
**Regulon_98**			
Operon_276	lilo_0429	LILO_RS02270	cysteine synthase
Operon_284	lilo_0441	LILO_RS02420	DNA primase
	lilo_0442	lilo_0442	major RNA polymerase sigma factor
Operon_856	lilo_1391	LILO_RS07210	hypothetical protein
Operon_862	lilo_1400	LILO_RS07255	transcription regulator
**Regulon_99**			
Operon_268	lilo_0414	LILO_RS02195	DegV family fatty acid-binding protein
	lilo_0415	LILO_RS02200	lipase/acylhydrolase
	lilo_0416	LILO_RS02205	beta-N-acetylhexosaminidase
	lilo_0417	LILO_RS02210	hypothetical protein
Operon_1254	lilo_2020	LILO_RS10470	arginine/ornitine antiporter
	lilo_2021	LILO_RS10475	hypothetical protein
	lilo_2022	LILO_RS10480	carbamate kinase
	lilo_2023	LILO_RS10485	carbamate kinase
	lilo_2024	LILO_RS10490	arginine/ornithine antiporter
Operon_858	lilo_1394	LILO_RS07225	ferric uptake regulator
	lilo_1395	LILO_RS07230	hypothetical protein
Operon_867	lilo_1405	LILO_RS07280	fructokinase
**Regulon_101**			
Operon_379	lilo_0594	LILO_RS03200	ABC transporter permease protein
	lilo_0595	LILO_RS03205	ABC transporter ATP-binding protein
Operon_75	lilo_0116	LILO_RS00645	queuine tRNA-ribosyltransferase
Operon_677	lilo_1088	LILO_RS05670	two-component sensor kinase YycG
Operon_629	lilo_1006	LILO_RS05245	ABC transporter ATP-binding protein
	lilo_1007	LILO_RS05250	ABC transporter permease protein
**Regulon_102**			
Operon_231	lilo_0361	LILO_RS01910	dTMP kinase
Operon_380	lilo_0596	LILO_RS03215	hypothetical protein
	lilo_0597	LILO_RS03220	Cro/CI family transcriptional regulator
Operon_656	lilo_1050	LILO_RS05470	putative RNA methylase
Operon_682	lilo_1094	LILO_RS05710	histidinol-phosphate aminotransferase
	lilo_1095	LILO_RS05715	ATP phosphoribosyltransferase regulatory subunit

	lilo_1096	LILO_RS05720	ATP phosphoribosyltransferase
	lilo_1097	LILO_RS05725	histidinol dehydrogenase
	lilo_1098	LILO_RS05730	SAM-dependent methyltransferase
	lilo_1099	LILO_RS05735	imidazoleglycerol-phosphate dehydratase
	lilo_1100	LILO_RS05740	kanamycin kinase
	lilo_1101	LILO_RS05745	amidotransferase
	lilo_1102	LILO_RS05750	phosphoribosylformimino-5-aminoimidazole carboxamideribotide isomerase
	lilo_1103	LILO_RS05755	imidazoleglycerol-phosphate synthase cyclase
	lilo_1104	LILO_RS05760	phosphoribosyl-AMP cyclohydrolase / phosphoribosyl-ATP pyrophosphohydrolase
	lilo_1105	LILO_RS05765	histidinol phosphatase
Operon_810	lilo_1311	LILO_RS06820	hypothetical protein
Operon_811	lilo_1312	LILO_RS06825	glycerol-3-phosphate dehydrogenase
	lilo_1313	LILO_RS06830	UTP-glucose-1-phosphate uridylyltransferase
**Regulon_103**			
Operon_8	lilo_0017	LILO_RS00095	beta-lactamase-type transpeptidase
	lilo_0018	LILO_RS00100	cell cycle protein/ tRNA(Ile)-lysidine synthetase
	lilo_0019	lilo_0019	hypoxantine-guanine phosphorybosyltransferase
Operon_158	lilo_0244	LILO_RS01335	mRNA degradation ribonuclease, metallo-beta-lactamase superfamily
Operon_376	lilo_0587	LILO_RS03165	UDP-N-acetylglucosamine 2-epimerase
	lilo_0588	LILO_RS03170	hypothetical protein
Operon_625	lilo_1000	LILO_RS05215	chloride channel protein
Operon_731	lilo_1176	LILO_RS06120	hypothetical protein
	lilo_1177	LILO_RS06125	hypothetical protein
	lilo_1178	LILO_RS06130	hypothetical protein
	lilo_0584	LILO_RS03150	N5-carboxyethyl-ornithine synthase
**Regulon_109**			
Operon_90	lilo_0136	LILO_RS00745	GNAT family acetyltransferase
Operon_661	lilo_1061	LILO_RS05525	homoserine dehydrogenase
	lilo_1062	LILO_RS05530	homoserine kinase
Operon_553	lilo_0883	LILO_RS04645	formyltetrahydrofolate synthetase
Operon_1341	lilo_2159	LILO_RS11230	metallo-phosphoesterase
**Regulon_110**			
Operon_23	lilo_0040	LILO_RS00245	tryptophanyl-tRNA synthetase
Operon_1239	lilo_1999	LILO_RS10365	UMP-kinase
Operon_558	lilo_0890	LILO_RS04680	protein-export protein SecG
Operon_1092	lilo_1777	LILO_RS09190	polysacharide biosynthesis protein
	lilo_1778	LILO_RS09195	regulatory protein
**Regulon_112**			
Operon_203	lilo_0328	LILO_RS01745	hypothetical protein
Operon_553	lilo_0883	LILO_RS04645	formyltetrahydrofolate synthetase
Operon_518	lilo_0820	LILO_RS04330	hypothetical protein

Operon_1104	lilo_1792	LILO_RS09270	putative amino acid permease
	lilo_1793	LILO_RS09275	peptidase T
	lilo_1794	LILO_RS09280	hypothetical protein
**Regulon_113**			
Operon_210	lilo_0338	LILO_RS01795	predicted membrane protein
Operon_698	lilo_1131	LILO_RS05900	IS3/IS911transposase, N-terminal fragment
Operon_777	lilo_1260	LILO_RS06565	phosphoglycerate mutase
Operon_1074	lilo_1739	LILO_RS09005	hypothetical protein
**Regulon_114**			
Operon_17	lilo_0031	LILO_RS00205	DNA repair protein RecO
Operon_33	lilo_0054	LILO_RS00315	50S ribosomal protein L33
	lilo_0055	LILO_RS00320	50S ribosomal protein L32
Operon_202	lilo_0327	LILO_RS01740	hypothetical protein
Operon_425	lilo_0381	LILO_RS02010	cellobiose-specific PTS system IIC component
	lilo_0382	LILO_RS02015	6-phospho-beta-glucosidase
**Regulon_115**			
Operon_25	lilo_0042	LILO_RS00255	amino acid permease
Operon_320	lilo_0495	LILO_RS02700	membrane-bound transport protein
Operon_455	lilo_0734	LILO_RS03905	queuosine transporter
Operon_479	lilo_0768	LILO_RS04075	tRNA (5-methylaminomethyl-2-thiouridylate)-methyltransferase
**Regulon_119**			
Operon_744	lilo_1195	LILO_RS06215	cation-transporting P-ATPase
Operon_213	lilo_0341	LILO_RS01810	C4-dicarboxylate transporter/malic acid transporter
Operon_780	lilo_1265	LILO_RS06590	transcriptional regulator/sugar kinase , xylose operon regulator
Operon_988	lilo_1591	LILO_RS08220	prophage pi2 protein 24
	lilo_1592	LILO_RS08225	putative replication initiator protein
	lilo_1593	LILO_RS08230	hypothetical protein
	lilo_1594	lilo_1594	putative teichoic acid/polysaccharide export protein
	lilo_1595	LILO_RS08240	O6-methylguanine-DNA methyltransferase
	lilo_1596	lilo_1596	hypothetical protein
	lilo_1597	lilo_1597	hypothetical protein
	lilo_1598	LILO_RS08270	prophage pi3 protein 39
	lilo_1599	LILO_RS08275	prophage pi1 protein 15
**Regulon_122**			
Operon_508	lilo_0809	LILO_RS04280	GTP pyrophosphokinase, RelA/SpoT superfamily
Operon_400	lilo_0645	LILO_RS03465	multidrug resistance ABC transporter ATP binding and permease protein
Operon_685	lilo_1109	LILO_RS05785	3-isopropylmalate dehydratase large subunit
	lilo_1110	LILO_RS05790	3-isopropylmalate dehydratase small subunit
	lilo_1111	LILO_RS05795	ABC transporter ATP binding protein
Operon_617	lilo_0986	LILO_RS05145	phosphate starvation inducible protein
	lilo_0987	LILO_RS05150	MutT/nudix family phosphohydrolase

Operon_977	lilo_1565	LILO_RS08085	lyzozyme M1 (1,4-beta-N-acetylmuramidase)
Operon_1060	lilo_1711	LILO_RS08865	hypothetical protein
Operon_1148	lilo_1861	LILO_RS09605	ribose-phosphate pyrophosphokinase
Operon_1288	lilo_2068	LILO_RS10725	30S ribosomal protein S14
	lilo_2069	LILO_RS10730	50S ribosomal protein L5
	lilo_2070	LILO_RS10735	50S ribosomal protein L24
Operon_1290	lilo_2078	LILO_RS10775	50S ribosomal protein L2
	lilo_2079	LILO_RS10780	50S ribosomal protein L23
	lilo_2080	LILO_RS10785	50S ribosomal protein L4
	lilo_2081	LILO_RS10790	50S ribosomal protein L3
	lilo_2082	LILO_RS10795	30S ribosomal protein S10
Operon_1304	lilo_2106	LILO_RS10920	DNA polymerase III, alpha chain
**Regulon_123**			
Operon_207	lilo_0334	LILO_RS01775	NADPH-dependent FMN reductase
	lilo_0335	LILO_RS01780	thiamine biosynthesis lipoprotein
Operon_753	lilo_1215	LILO_RS06345	ABC transporter ATP binding and permease protein
	lilo_1216	LILO_RS06350	ABC transporter ATP binding and permease protein
	lilo_1217	LILO_RS06355	hypothetical protein
	lilo_1218	LILO_RS06360	hypothetical protein
Operon_843	lilo_1362	LILO_RS07055	hypothetical protein
	lilo_1363	LILO_RS07060	cation transporter
Operon_1245	lilo_2005	LILO_RS10395	GTP-binding protein TypA/BipA
**Regulon_124**			
Operon_235	lilo_0368	LILO_RS01945	diphosphomevalonate decarboxylase
	lilo_0369	LILO_RS01950	phosphomevalonate kinase
	lilo_0370	LILO_RS01955	isopentenyl-diphosphate delta-isomerase
Operon_419	lilo_0674	LILO_RS03605	signaling protein
	lilo_0675	LILO_RS03610	50S ribosomal protein L9
	lilo_0676	LILO_RS03615	replicative DNA helicase
Operon_159	lilo_0245	LILO_RS01340	predicted RNA binding protein, contains RRM domain
Operon_464	lilo_0751	LILO_RS03990	ribose-phosphate pyrophosphokinase
Operon_1229	lilo_1984	LILO_RS10290	ABC transporter permease protein
	lilo_1985	LILO_RS10295	ABC transporter ATP binding protein
	lilo_1986	LILO_RS10300	putative glycerophosphoryl diester phosphodiesterase
Operon_841	lilo_1356	LILO_RS07025	cytidine deaminase
	lilo_1357	LILO_RS07030	deoxyribose-phosphate aldolase
	lilo_1358	LILO_RS07035	hypothetical protein
	lilo_1359	LILO_RS07040	pyrimidine-nucleoside phosphorylase
	lilo_1360	LILO_RS07045	hypothetical protein
Operon_1297	lilo_2090	LILO_RS10835	penicillin-binding protein 2a
**Regulon_125**			
Operon_611	lilo_0973	LILO_RS05080	DNA replication protein DnaD

	lilo_0974	LILO_RS05085	endonuclease III
	lilo_0975	LILO_RS05090	hypothetical protein
	lilo_0976	LILO_RS05095	NIF3 (NGG1p interacting factor 3) family protein
Operon_235	lilo_0368	LILO_RS01945	diphosphomevalonate decarboxylase
	lilo_0369	LILO_RS01950	phosphomevalonate kinase
	lilo_0370	LILO_RS01955	isopentenyl-diphosphate delta-isomerase
Operon_646	lilo_1036	LILO_RS05400	penicillin acylase
	lilo_1037	LILO_RS05405	hypothetical protein
Operon_1350	lilo_1350	LILO_RS06995	hypothetical protein
**Regulon_126**			
Operon_681	lilo_1093	LILO_RS05705	ribonuclease
Operon_969	lilo_1557	LILO_RS08045	D-mannonate dehydratase
Operon_46	lilo_0077	LILO_RS00435	phosphocarrier protein HPr
	lilo_0078	LILO_RS00440	phosphotransferase system, enzyme I/ phosphoenolpyruvate-protein phosphotransferase
Operon_1280	lilo_2051	LILO_RS10630	DNA repair protein
Operon_611	lilo_0973	LILO_RS05080	DNA replication protein DnaD
	lilo_0974	LILO_RS05085	endonuclease III
	lilo_0975	LILO_RS05090	hypothetical protein
	lilo_0976	LILO_RS05095	NIF3 (NGG1p interacting factor 3) family protein
**Regulon_127**			
Operon_280	lilo_0435	LILO_RS02300	arsenate reductase family protein
Operon_273	lilo_0426	LILO_RS02255	pyridoxal-phosphate dependent aminotransferase
Operon_920	lilo_0449	LILO_RS02460	polypeptide deformylase
Operon_512	lilo_0814	LILO_RS04305	nicotinamide mononucleotide transporter
Operon_279	lilo_0434	LILO_RS02295	hypothetical protein
**Regulon_130**			
Operon_32	lilo_0050	LILO_RS00295	hypothetical protein
	lilo_0051	LILO_RS00300	alpha/beta hydrolase
	lilo_0052	LILO_RS00305	glyoxalase family protein
	lilo_0053	LILO_RS00310	flavin reductase family protein
Operon_104	lilo_0153	LILO_RS00835	dihydroxyacetone kinase family protein
	lilo_0154	LILO_RS00840	Gls24 family general stress protein
Operon_253	lilo_0391	LILO_RS02065	GntR family transcriptional regulator
Operon_1309	lilo_2117	LILO_RS10975	glycosyltransferase
**Regulon_135**			
Operon_348	lilo_0533	LILO_RS02895	dephospho-CoA kinase
	lilo_0534	LILO_RS02900	multidrug resistance efflux pump
	lilo_0535	LILO_RS02905	50S ribosomal protein L33
Operon_611	lilo_0973	LILO_RS05080	DNA replication protein DnaD
	lilo_0974	LILO_RS05085	endonuclease III
	lilo_0975	LILO_RS05090	hypothetical protein
	lilo_0976	LILO_RS05095	NIF3 (NGG1p interacting factor 3) family protein

Operon_35	lilo_0057	LILO_RS00330	chromosome partitioning protein
	lilo_0058	LILO_RS00335	chromosome segregation helicase
	lilo_0059	LILO_RS00340	acetyl transferase
Operon_615	lilo_0982	LILO_RS05125	50S ribosomal protein L21
	lilo_0983	LILO_RS05130	hypothetical protein
	lilo_0984	LILO_RS05135	50S ribosomal protein L27
Operon_650	lilo_1042	LILO_RS05430	dCMP deaminase
Operon_1343	lilo_2161	LILO_RS11240	single-strand binding protein
	lilo_2162	LILO_RS11245	30S ribosomal protein S6
Operon_1245	lilo_2005	LILO_RS10395	GTP-binding protein TypA/BipA
**Regulon_139**			
Operon_199	lilo_0322	LILO_RS01715	amino acid permease
	lilo_0323	LILO_RS01720	amino acid permease
Operon_223	lilo_0366	LILO_RS01935	hypothetical protein
Operon_877	lilo_1430	LILO_RS07405	xylose operon regulator
Operon_927	lilo_1496	LILO_RS07735	hydroxymethylglutaryl-CoA synthase
Operon_1241	lilo_2001	LILO_RS10375	acetate kinase
**Regulon_140**			
Operon_1004	lilo_1627	LILO_RS08425	thioredoxin reductase
Operon_199	lilo_0322	LILO_RS01715	amino acid permease
	lilo_0323	LILO_RS01720	amino acid permease
Operon_204	lilo_0329	LILO_RS01750	GTP pyrophosphokinase
	lilo_0330	LILO_RS01755	probable inorganic polyphosphate/ATP-NAD kinase
	lilo_0331	LILO_RS01760	pseudouridine synthase
Operon_1164	lilo_1893	LILO_RS09765	hypothetical protein
	lilo_1894	LILO_RS09775	hypothetical protein
	lilo_1895	LILO_RS09780	hypothetical protein
**Regulon_147**			
Operon_150	lilo_0236	LILO_RS01285	putative 2,3,4,5-tetrahydropyridine-2-carboxylate N-succinyltransferase
Operon_921	lilo_1490	LILO_RS07705	hypothetical protein
Operon_932	lilo_1502	LILO_RS07760	hypothetical protein
Operon_1214	lilo_1966	LILO_RS10195	gamma-carboxymuconolactone decarboxylase
**Regulon_149**			
Operon_808	lilo_1309	LILO_RS06810	N-acetylglucosamine-6-phosphate deacetylase
Operon_1151	lilo_1865	LILO_RS09625	elongation factor EF-Tu
Operon_931	lilo_1501	LILO_RS07755	transcription regulator
Operon_1120	lilo_1819	LILO_RS09400	integrase
**Regulon_153**			
Operon_1242	lilo_2002	LILO_RS10380	acetate kinase
Operon_677	lilo_1087	LILO_RS05665	histidine kinase
	lilo_1088	LILO_RS05670	two-component sensor kinase YycG
Operon_1329	lilo_2143	LILO_RS11130	MarR family transcriptional regulator

Operon_1089	lilo_1771	LILO_RS09160	hypothetical protein
**Regulon_156**			
Operon_585	lilo_0933	LILO_RS04895	CorA family Mg2+/Co2+ transporter
Operon_613	lilo_0978	LILO_RS05105	HAD superfamily hydrolase
	lilo_0979	LILO_RS05110	dGTP triphosphohydrolase
Operon_960	lilo_1543	LILO_RS07970	cation transport ATPase
Operon_1059	lilo_1710	LILO_RS08860	hypothetical protein
**Regulon_157**			
Operon_79	lilo_0120	LILO_RS00665	DNA/RNA non-specific endonuclease
Operon_95	lilo_0142	LILO_RS00780	5'-nucleotidase
	lilo_0143	LILO_RS00785	hypothetical protein
Operon_498	lilo_0796	LILO_RS04215	DNA repair protein RecN
Operon_959	lilo_1542	LILO_RS07965	hypothetical protein
**Regulon_158**			
Operon_228	lilo_0357	LILO_RS01890	60 KD chaperonin
Operon_959	lilo_1542	LILO_RS07965	hypothetical protein
Operon_964	lilo_1549	LILO_RS08000	transketolase
Operon_1275	lilo_2044	LILO_RS10595	hypothetical protein
**Regulon_159**			
Operon_114	lilo_0184	LILO_RS01005	GTP-binding protein HflX
	lilo_0185	LILO_RS01010	CRS1/YhbY family RNA binding protein
	lilo_0186	LILO_RS01015	nicotinate-nucleotide adenylyltransferase
	lilo_0187	LILO_RS01020	HAD superfamily hydrolase
	lilo_0188	LILO_RS01025	GNAT family acetyltransferase
Operon_964	lilo_1126	LILO_RS05870	RecA protein
	lilo_1127	LILO_RS05880	SOS responce UmuC protein
Operon_173	lilo_0271	LILO_RS01460	UDP-N-acetylglucosamine 1-carboxyvinyltransferase
	lilo_0272	LILO_RS01465	hypothetical protein
Operon_1176	lilo_1913	LILO_RS09870	fructose-bisphosphate aldolase
Operon_247	lilo_0384	LILO_RS02030	DNA ligase
Operon_648	lilo_1040	LILO_RS05420	oxygen-independent coproporphyrogen III oxidase
Operon_1314	lilo_2122	LILO_RS11000	DNA polymerase I
**Regulon_161**			
Operon_429	lilo_0689	LILO_RS03690	hypothetical protein
	lilo_0690	LILO_RS03695	sortase, putative
	lilo_0691	LILO_RS03700	hypothetical protein
Operon_253	lilo_0391	LILO_RS02065	GntR family transcriptional regulator
Operon_639	lilo_1022	LILO_RS05330	ATP-dependent helicase PcrA
	lilo_1023	LILO_RS05335	mutator protein MutT
	lilo_1024	LILO_RS05340	DNA-3-methyladenine glycosidase I
Operon_254	lilo_0392	LILO_RS02070	PTS system, beta-glucosides-specific IIA component
**Regulon_162**			
Operon_83	lilo_0125	LILO_RS00690	transcriptional regulator

Operon_1348	lilo_2167	LILO_RS11270	DNA polymerase III, subunits beta and tau
	lilo_2168	LILO_RS11275	aminoglycoside phosphotransferase
	lilo_2169	LILO_RS11280	GAF domain-containing protein
Operon_86	lilo_0131	LILO_RS00720	MatE family Na+ driven multidrug efflux pump
Operon_195	lilo_0318	LILO_RS01695	GTP-binding protein Era
Operon_954	lilo_1537	LILO_RS07940	branched-chain amino acid transport protein AzlC
**Regulon_163**			
Operon_7	lilo_0014	LILO_RS00080	S4 domain-containing RNA-binding protein
	lilo_0015	LILO_RS00085	septum formation initiator
	lilo_0016	LILO_RS00090	hypothetical protein
Operon_83	lilo_0125	LILO_RS00690	transcriptional regulator
Operon_336	lilo_0515	LILO_RS02800	hypothetical protein
Operon_569	lilo_0912	LILO_RS04790	hypothetical protein
Operon_1057	lilo_1706	LILO_RS08840	maturation protein
Operon_1292	lilo_2084	LILO_RS10805	hypothetical protein
	lilo_2085	LILO_RS10810	threonine synthase
**Regulon_165**			
Operon_21	lilo_0038	LILO_RS00235	glycerophosphoryl diester phosphodiesterase
Operon_657	lilo_1051	LILO_RS05475	dihydropholate reductase
	lilo_1052	LILO_RS05480	hypothetical protein
Operon_757	lilo_1230	LILO_RS06420	glutamate synthase (NADPH) small chain
	lilo_1231	LILO_RS06425	glutamate synthase (NADPH) large chain
Operon_786	lilo_1273	LILO_RS06635	glycosyltransferase related enzyme
	lilo_1274	LILO_RS06640	phosphotransferase system, fructose-specific IIC component
	lilo_1275	LILO_RS06645	fusion of IIA, IIB and IIC component of mannitol/fructose-specific phosphotransferase system mannitol/fructose-specific
Operon_1237	lilo_1996	LILO_RS10350	hypothetical protein
	lilo_1997	LILO_RS10355	hypothetical protein
**Regulon_170**			
Operon_626	lilo_1001	LILO_RS05220	nicotinate-nucleotide adenylyltransferase
Operon_755	lilo_1228	LILO_RS06410	hypothetical protein
Operon_1271	lilo_2040	LILO_RS10575	amino acid ABC transporter substrate binding protein
Operon_1167	lilo_1901	LILO_RS09810	DNA-directed RNA polymerase subunit omega
**Regulon_175**			
Operon_208	lilo_0336	LILO_RS01785	lysyl-tRNA synthetase
Operon_517	lilo_0819	LILO_RS04325	ABC transporter phage infection protein
Operon_1287	lilo_2067	LILO_RS10720	hypothetical protein
Operon_1316	lilo_2124	LILO_RS11035	competence regulator
**Regulon_176**			
Operon_289	lilo_0448	LILO_RS02455	glyceraldehyde 3-phosphate dehydrogenase
Operon_302	lilo_0466	LILO_RS02550	carotenoid biosynthetic protein CrtK
Operon_349	lilo_0536	LILO_RS02910	cell division protein FtsW

	lilo_0537	LILO_RS02915	pyruvate carboxylase
Operon_433	lilo_0696	LILO_RS03725	hypothetical protein
	lilo_0697	LILO_RS03730	50S ribosomal protein L7AE
	lilo_0698	LILO_RS03735	translation initiation factor IF-2
**Regulon_179**			
Operon_205	lilo_0332	LILO_RS01765	peptidyl-prolyl cis-trans isomerase
Operon_344	lilo_0527	LILO_RS02865	ABC transporter ATP-binding protein
	lilo_0528	LILO_RS02870	ABC transporter permease protein
	lilo_0529	LILO_RS02875	TetR family transcriptional regulator
Operon_806	lilo_1305	LILO_RS06790	L-lactate dehydrogenase
Operon_847	lilo_1368	LILO_RS07085	hypothetical protein
**Regulon_217**			
Operon_44	lilo_0075	LILO_RS00425	preprotein translocase SecA subunit
Operon_392	lilo_0626	LILO_RS03380	elongation factor EF-P
Operon_1154	lilo_1868	LILO_RS09640	cell division protein
	lilo_1869	LILO_RS09645	hypothetical protein
	lilo_1870	LILO_RS09650	YggT family protein
	lilo_1871	LILO_RS09655	hypothetical protein
	lilo_1872	LILO_RS09660	hypothetical protein
	lilo_1873	LILO_RS09665	cell division protein FtsZ
	lilo_1874	LILO_RS09670	cell division protein FtsA
Operon_1175	lilo_1912	LILO_RS09865	N-acetylmuramidase
Operon_432	lilo_0695	LILO_RS03720	transcription termination protein NusA
Operon_1324	lilo_2135	LILO_RS11090	PDZ domain-containing protein
	lilo_2136	LILO_RS11095	pantetheine-phosphate adenylyltransferase
	lilo_2137	LILO_RS11100	methyltransferase
**Regulon_218**			
Operon_171	lilo_0268	LILO_RS01445	PadR family transcriptional regulator
Operon_1102	lilo_1790	LILO_RS09260	alcohol dehydrogenase
Operon_1278	lilo_2049	LILO_RS10620	hypothetical protein
Operon_1338	lilo_2154	LILO_RS11205	gluconate transport protein
**Regulon_219**			
Operon_52	lilo_0085	LILO_RS00480	glycosil transferase
	lilo_0086	LILO_RS00485	hypothetical protein
	lilo_0068	LILO_RS00385	glycosil transferase
Operon_108	lilo_0164	LILO_RS00890	rhamnosyltransferase
	lilo_0165	LILO_RS00895	rhamnosyltransferase
	lilo_0166	LILO_RS00900	polysaccharide ABC transporter permease protein
	lilo_0167	LILO_RS00905	polysaccharide ABC transporter ATP-binding protein
	lilo_0168	LILO_RS00910	predicted membrane protein
	lilo_0169	LILO_RS00915	glycosyltransferase/ beta-1,3-N-acetylglucosaminyltransferase
	lilo_0170	LILO_RS00920	lipopolysaccharide biosynthesis protein/ bifunctional alpha-L-Rha alpha-1,2-L-rhamnosyltransferase/alpha-

			L-Rha alpha-1,3-L-rhamnosyltransferase
Operon_887	lilo_1442	LILO_RS07470	transcription regulator
Operon_1298	lilo_2091	LILO_RS10840	hypothetical protein
	lilo_2092	LILO_RS10845	hypothetical protein
**Regulon_220**			
Operon_559	lilo_0891	LILO_RS04685	ribonuclease
	lilo_0892	LILO_RS04690	isochorismatase family protein
Operon_998	lilo_1615	LILO_RS08360	ribose ABC transporter substrate binding protein
	lilo_1616	LILO_RS08365	ribose ABC transporter permease protein
	lilo_1617	LILO_RS08370	ribose ABC transporter ATP binding protein
	lilo_1618	LILO_RS08375	ribose ABC transporter permease protein
	lilo_1619	LILO_RS08380	ribokinase
	lilo_1620	LILO_RS08385	ribose operon repressor
Operon_567	lilo_0904	LILO_RS04750	myosin-crossreactive antigen
	lilo_0905	LILO_RS04755	amino acid aminohydrolase
Operon_1026	lilo_1981	LILO_RS10275	hypothetical protein
**Regulon_221**			
Operon_295	lilo_0456	LILO_RS02500	OxaA/YidC family membrane protein
Operon_1280	lilo_2051	LILO_RS10630	DNA repair protein
Operon_781	lilo_1266	LILO_RS06595	indole-3-pyruvate decarboxylase
**Regulon_223**			
Operon_137	lilo_0217	LILO_RS01175	ABC transporter ATP binding protein
	lilo_0218	LILO_RS01180	ABC transporter ATP binding and permease protein
Operon_322	lilo_0497	LILO_RS02710	mathionine aminopeptidase
	lilo_0498	LILO_RS02715	putative tRNA-processing ribonuclease BN
Operon_473	lilo_1194	LILO_RS06210	iron-dependent repressor
Operon_1369	lilo_2198	LILO_RS11435	rod shape-determining protein MreD
	lilo_2199	LILO_RS11440	rod shape-determining protein MreC
**Regulon_224**			
Operon_142	lilo_0224	LILO_RS01210	amidase/ cysteine hydrolase, isochorismatse family
Operon_79	lilo_0120	LILO_RS00665	DNA/RNA non-specific endonuclease
Operon_435	lilo_0700	LILO_RS03745	mannose-6-phosphate isomerase
Operon_1156	lilo_1877	LILO_RS09685	hypothetical protein
**Regulon_226**			
Operon_149	lilo_0233	LILO_RS01265	cobalt ABC transporter ATP-binding protein
	lilo_0234	LILO_RS01270	cobalt ABC transporter permease
	lilo_0235	LILO_RS01275	thiol-disulfide isomerase and thioredoxin
Operon_144	lilo_0227	LILO_RS01225	N-acetylmuramidase
Operon_333	lilo_0512	LILO_RS02780	tRNA isopentenyltransferase
Operon_1024	lilo_1651	LILO_RS08560	hypothetical protein
**Regulon_231**			
Operon_112	lilo_0181	LILO_RS00990	IMP dehydrogenase
Operon_106	lilo_0156	LILO_RS00850	hypothetical protein

Operon_588	lilo_0940	LILO_RS04930	hypothetical protein
Operon_1261	lilo_2029	LILO_RS10520	UDP-N-acetylmuramate-alanine ligase
Operon_651	lilo_1043	LILO_RS05435	acyl-ACP thioesterase
	lilo_1044	LILO_RS05440	N-acetylglucosamine catabolic protein
Operon_1268	lilo_2036	LILO_RS10555	hypothetical protein
Operon_1069	lilo_1726	LILO_RS08940	prephenate dehydrogenase
	lilo_1727	LILO_RS08945	sensor protein kinase
	lilo_1728	LILO_RS08950	two-component system regulator
	lilo_1729	LILO_RS08955	hypothetical protein
**Regulon_233**			
Operon_398	lilo_0639	LILO_RS03440	cytochrome D ABC transporter ATP binding and permease protein
	lilo_0640	LILO_RS03445	cytochrome D ABC transporter ATP binding and permease protein
Operon_1175	lilo_1912	LILO_RS09865	N-acetylmuramidase
Operon_1168	lilo_1902	LILO_RS09815	guanylate kinase
Operon_1184	lilo_1861	LILO_RS09605	ribose-phosphate pyrophosphokinase
**Regulon_237**			
Operon_670	lilo_1077	LILO_RS05615	cardiolipin synthase
Operon_237	lilo_0372	LILO_RS01965	carbon starvation protein
Operon_936	lilo_1510	LILO_RS07800	hypothetical protein
	lilo_1511	LILO_RS07805	GTP-binding protein Obg
Operon_1317	lilo_2125	LILO_RS11040	septation ring formation regulator
**Regulon_238**			
Operon_100	lilo_0148	LILO_RS00810	CAAX amino terminal protease family protein
Operon_99	lilo_0147	LILO_RS00805	1,4-dihydroxy-2-naphthoate polyprenyltransferase
Operon_417	lilo_0672	LILO_RS03595	aspartate kinase
Operon_1228	lilo_1983	LILO_RS10285	putative copper homeostasis protein
**Regulon_240**			
Operon_234	lilo_0367	LILO_RS01940	mevalonate kinase
Operon_112	lilo_0181	LILO_RS00990	IMP dehydrogenase
Operon_473	lilo_0760	LILO_RS04035	NADH dehydrogenase
Operon_563	lilo_0896	LILO_RS04710	hypothetical protein
Operon_687	lilo_1117	LILO_RS05825	alpha-acetolactate decarboxylase
	lilo_1118	LILO_RS05830	regulatory protein AldR
**Regulon_244**			
Operon_67	lilo_0108	LILO_RS00600	1,4-alpha-glucan branching enzyme
Operon_927	lilo_1496	LILO_RS07735	hydroxymethylglutaryl-CoA synthase
Operon_353	lilo_0543	LILO_RS02945	arsenate reductase family protein
Operon_1099	lilo_1787	LILO_RS09240	hypothetical protein
**Regulon_248**			
Operon_815	lilo_1322	LILO_RS06875	drug-export protein
Operon_1327	lilo_2141	LILO_RS11120	hypothetical protein
Operon_1045	lilo_1686	LILO_RS08735	hypothetical protein

Operon_861	lilo_1398	LILO_RS07245	oxidoreductase, short-chain dehydrogenase/reductasefamily protein
	lilo_1399	LILO_RS07250	1-deoxyxylulose-5-phosphate synthase
**Regulon_252**			
Operon_647	lilo_1038	LILO_RS05410	hypothetical protein
	lilo_1039	LILO_RS05415	hypothetical protein
Operon_644	lilo_1031	LILO_RS05375	hypothetical protein
	lilo_1032	LILO_RS05380	N-acetylmuramic acid-6-phosphate etherase
	lilo_1033	LILO_RS05385	sucrose-specific PTS system IIBC component
	lilo_1034	LILO_RS05390	RpiR family transcriptional regulator
Operon_652	lilo_1045	LILO_RS05445	hypothetical protein
Operon_1237	lilo_1996	LILO_RS10350	hypothetical protein
	lilo_1997	LILO_RS10355	hypothetical protein
Operon_1082	lilo_1753	LILO_RS09070	lipase
**Regulon_256**			
Operon_923	lilo_1492	LILO_RS07715	hypothetical protein
Operon_1069	lilo_1726	LILO_RS08940	prephenate dehydrogenase
	lilo_1727	LILO_RS08945	sensor protein kinase
	lilo_1728	LILO_RS08950	two-component system regulator
	lilo_1729	LILO_RS08955	hypothetical protein
Operon_81	lilo_0123	LILO_RS00680	heme/copper-type cytochrome/quinol oxidase subunit 1
Operon_1008	lilo_1631	LILO_RS08445	hypothetical protein
Operon_1201	lilo_1945	LILO_RS10085	hypothetical protein
**Regulon_259**			
Operon_311	lilo_0478	LILO_RS02610	hypothetical protein
Operon_170	lilo_0266	LILO_RS01435	MarR family transcriptional regulator
	lilo_0267	LILO_RS01440	co/Zn/cd efflux protein
Operon_1036	lilo_1671	LILO_RS08660	transcription regulator
Operon_550	lilo_0880	LILO_RS04630	purine-nucleoside phosphorylase
**Regulon_262**			
Operon_172	lilo_0269	LILO_RS01450	ABC transporter ATP binding and permease protein
	lilo_0270	LILO_RS01455	ABC transporter ATP binding and permease protein
Operon_820	lilo_1333	LILO_RS06930	carbamoyl-phosphate synthase large chain
Operon_313	lilo_0484	LILO_RS02640	protoporphyrinogen oxidase
	lilo_0485	LILO_RS02645	GNAT family acetyltransferase
	lilo_0486	lilo_0486	Sua5/YciO/YrdC/YwlC family RNA-binding protein
Operon_1135	lilo_1840	LILO_RS09505	oligopeptide ABC transporter substrate binding protein
	lilo_1841	LILO_RS09510	oligopeptide ABC transporter permease protein
	lilo_1842	LILO_RS09515	oligopeptide ABC transporter permease protein
	lilo_1843	LILO_RS09520	oligopeptide ABC transporter ATP binding protein
	lilo_1844	LILO_RS09525	oligopeptide ABC transporter ATP binding protein
**Regulon_263**			

Operon_1042	lilo_1681	LILO_RS08710	ferredoxin
Operon_501	lilo_0799	LILO_RS04230	transporter
	lilo_0800	LILO_RS04235	hypothetical protein
Operon_395	lilo_0635	LILO_RS03425	di-/tripeptide transporter
Operon_149	lilo_0232	LILO_RS01255	cobalt ABC transporter ATP-binding protein
	lilo_0233	LILO_RS01265	cobalt ABC transporter ATP-binding protein
	lilo_0234	LILO_RS01270	cobalt ABC transporter permease
	lilo_0235	LILO_RS01275	thiol-disulfide isomerase and thioredoxin
**Regulon_265**			
Operon_382	lilo_0599	LILO_RS03230	Cro/CI family transcriptional regulator
	lilo_0600	LILO_RS03235	hypothetical protein
	lilo_0601	LILO_RS03240	ribosomal protein L5
	lilo_0602	LILO_RS03245	putative nucleolar protein
	lilo_0603	LILO_RS03250	putative transposon protein
Operon_488	lilo_0780	LILO_RS04135	arsenate reductase family protein
Operon_500	lilo_0798	LILO_RS04225	hypothetical protein
Operon_1029	lilo_1661	LILO_RS08610	neopullulanase
**Regulon_267**			
Operon_496	lilo_0790	LILO_RS04185	exonuclease VII large subunit
	lilo_0791	LILO_RS04190	exonuclease VII small subunit
Operon_1029	lilo_1661	LILO_RS08610	neopullulanase
Operon_1033	lilo_1668	LILO_RS08645	hypothetical protein
Operon_1174	lilo_1909	LILO_RS09850	hypothetical protein
	lilo_1910	LILO_RS09855	hypothetical protein
	lilo_1911	LILO_RS09860	hypothetical protein
Operon_1343	lilo_2161	LILO_RS11240	single-strand binding protein
	lilo_2162	LILO_RS11245	30S ribosomal protein S6
**Regulon_269**			
Operon_870	lilo_1412	LILO_RS07315	sugar ABC transporter substrate-binding protein
	lilo_1413	LILO_RS07320	sugar ABC transporter permease protein
	lilo_1414	LILO_RS07325	sugar ABC transporter substrate-binding protein
Operon_1204	lilo_1948	LILO_RS10100	putative heat shock protein
	lilo_1949	LILO_RS10105	hypothetical protein
Operon_672	lilo_1079	LILO_RS05625	putative N-acylglucosamine-6-phosphate 2-epimerase
Operon_349	lilo_0536	LILO_RS02910	cell division protein FtsW
	lilo_0537	LILO_RS02915	pyruvate carboxylase
**Regulon_273**			
Operon_45	lilo_0076	LILO_RS00430	phospho-2-dehydro-3-deoxyheptonate aldolase
Operon_1039	lilo_1677	LILO_RS08690	two-component system regulator
Operon_50	lilo_0083	LILO_RS00470	SugE protein
Operon_1150	lilo_1863	LILO_RS09615	pyridoxal-phosphate dependent aminotransferase NifS
	lilo_1864	LILO_RS09620	hypothetical protein
Operon_690	lilo_1122	LILO_RS05850	tyrosine recombinase

**Regulon_276**			
Operon_345	lilo_0530	LILO_RS02880	long-chain acyl-CoA synthetase
Operon_913	lilo_1480	LILO_RS07655	oxidoreductase
Operon_1195	lilo_1938	LILO_RS10050	chitinase
	lilo_1939	LILO_RS10055	chitin binding protein
Operon_1300	lilo_2094	LILO_RS10855	pseudouridine synthase
Operon_1060	lilo_1711	LILO_RS08865	hypothetical protein
**Regulon_277**			
Operon_55	lilo_0090	LILO_RS00510	TetR family transcriptional regulator
Operon_1180	lilo_1917	LILO_RS09940	hypothetical protein
	lilo_1918	LILO_RS09945	putative Mn and Fe transporters
Operon_1194	lilo_1937	LILO_RS10045	hypothetical protein
Operon_1269	lilo_2037	LILO_RS10560	glycerol uptake facilitator related permease
**Regulon_280**			
Operon_1192	lilo_1934	LILO_RS10030	phenylalanyl-tRNA synthetase beta chain
	lilo_1935	LILO_RS10035	phenylalanyl-tRNA synthetase alpha chain
Operon_38	lilo_0065	LILO_RS00370	hypothetical protein
	lilo_0066	LILO_RS00375	predicted xylanase/chitin deacetilase
Operon_1065	lilo_1718	LILO_RS08900	hypothetical protein
Operon_1336	lilo_2152	LILO_RS11175	Mucus-binding protein, LPXTG-anchored
**Regulon_281**			
Operon_42	lilo_0072	LILO_RS00410	1-acyl-sn-glycerol-3-phosphate acyltransferase
Operon_921	lilo_1490	LILO_RS07705	hypothetical protein
Operon_1128	lilo_1831	LILO_RS09460	asparaginyl-tRNA synthetase
	lilo_1832	LILO_RS09465	hypothetical protein
	lilo_1833	LILO_RS09470	aspartate aminotransferase
	lilo_1834	LILO_RS09475	hypothetical protein
Operon_1130	lilo_1836	LILO_RS09485	hypothetical protein
**Regulon_285**			
Operon_283	lilo_0440	LILO_RS02325	trigger factor
Operon_149	lilo_0232	LILO_RS01255	cobalt ABC transporter ATP-binding protein
	lilo_0233	LILO_RS01265	cobalt ABC transporter ATP-binding protein
	lilo_0234	LILO_RS01270	cobalt ABC transporter permease
	lilo_0235	LILO_RS01275	thiol-disulfide isomerase and thioredoxin
Operon_690	lilo_1122	LILO_RS05850	tyrosine recombinase
Operon_393	lilo_0627	LILO_RS03385	Gls24 family general stress protein
	lilo_0628	LILO_RS03390	transcription termination protein NusB
Operon_1301	lilo_2095	LILO_RS10860	ABC-type Mn2+/Zn2+ transport system, permease component
	lilo_2096	LILO_RS10865	zinc ABC transporter ATP binding protein
	lilo_2097	LILO_RS10870	zinc ABC transporter substrate binding protein
	lilo_2098	LILO_RS10875	zinc transport transcription regulator
Operon_813	lilo_1318	LILO_RS06855	hypothetical protein

	lilo_1319	LILO_RS06860	orotidine-phosphate decarboxylase
**Regulon_287**			
Operon_1000	lilo_1622	LILO_RS08395	3-dehydroquinate dehydratase
Operon_309	lilo_0476	LILO_RS02600	phage protein
Operon_1109	lilo_1801	LILO_RS09320	universal stress protein UspA related nucleotide-binding protein
Operon_1249	lilo_2013	LILO_RS10435	hypothetical protein
Operon_1337	lilo_2153	LILO_RS11180	regulatory protein recX
**Regulon_288**			
Operon_181	lilo_0290	LILO_RS01555	cobalt ABC transporter permease
	lilo_0291	LILO_RS01560	cobalt ABC transporter ATP-binding protein
	lilo_0292	LILO_RS01565	cobalt ABC transporter permease
Operon_292	lilo_0452	LILO_RS02475	glutamate or arginine ABC transporter substrate binding protein
	lilo_0453	LILO_RS02480	acetylornithine deacetylase
Operon_467	lilo_0754	LILO_RS04005	HTH-type transcriptional regulator
Operon_817	lilo_1324	LILO_RS06885	hypothetical protein
	lilo_1325	LILO_RS06890	hypothetical protein
**Regulon_294**			
Operon_359	lilo_0552	LILO_RS02995	branched-chain amino acid transport system carrier protein
Operon_528	lilo_0835	LILO_RS04410	cell division protein FtsW
	lilo_0836	LILO_RS04415	LytR family transcriptional regulator
Operon_1280	lilo_2051	LILO_RS10630	DNA repair protein
Operon_1073	lilo_1737	LILO_RS08995	3-dehydroquinate synthase
**Regulon_296**			
Operon_297	lilo_0458	LILO_RS02510	hypothetical membrane protein
	lilo_0459	LILO_RS02515	phosphopantothenoylcysteine synthase/decarboxylase
	lilo_0460	LILO_RS02520	phosphopantothenate--cysteine ligase
Operon_661	lilo_1061	LILO_RS05525	homoserine dehydrogenase
	lilo_1062	LILO_RS05530	homoserine kinase
Operon_477	lilo_0766	LILO_RS04065	ABC transporter permease
Operon_880	lilo_1435	LILO_RS07435	transport protein
Operon_1041	lilo_1679	LILO_RS08700	cytidine monophosphate kinase
**Regulon_298**			
Operon_465	lilo_0752	LILO_RS03995	putative regulator
Operon_1003	lilo_1625	LILO_RS08415	DNA mismatch repair protein
	lilo_1626	LILO_RS08420	hypothetical protein
Operon_940	lilo_1517	LILO_RS07835	sensor protein kinase
	lilo_1518	LILO_RS07840	two-component system regulator
Operon_947	lilo_1526	LILO_RS07880	carbamoyl-phosphate synthase small chain
	lilo_1527	LILO_RS07885	aspartate carbamoyltransferase catalytic chain
Operon_1283	lilo_2059	LILO_RS10680	adenylate kinase
	lilo_2060	LILO_RS10685	preprotein translocase SecY subunit

**Regulon_301**			
Operon_621	lilo_0994	LILO_RS05185	VanZ family protein
	lilo_0995	LILO_RS05190	hypothetical protein
Operon_1289	lilo_2071	LILO_RS10740	50S ribosomal protein L14
	lilo_2072	LILO_RS10745	30S ribosomal protein S17
	lilo_2073	LILO_RS10750	50S ribosomal protein L29
	lilo_2074	LILO_RS10755	50S ribosomal protein L16
	lilo_2075	LILO_RS10760	30S ribosomal protein S3
	lilo_2076	LILO_RS10765	50S ribosomal protein L22
	lilo_2077	LILO_RS10770	30S ribosomal protein S19
Operon_586	lilo_0934	LILO_RS04900	diaminohydroxyphosphoribosylaminopyrimidine deaminase / 5-amino-6-(5-phosphoribosylamino)uracil reductase
	lilo_0935	LILO_RS04905	riboflavin synthase alpha chain
	lilo_0936	LILO_RS04910	GTP cyclohydrolase II / 3,4-dihydroxy-2-butanone 4-phosphate synthase
	lilo_0937	LILO_RS04915	riboflavin synthase beta chain
	lilo_1219	LILO_RS06365	hypothetical protein
**Regulon_302**			
Operon_98	lilo_0146	LILO_RS00800	prenyl transferase
Operon_1105	lilo_1796	LILO_RS09290	pyruvate-formate lyase activating enzyme
Operon_1396	lilo_0007	LILO_RS00040	GTP-dependent nucleic acid-binding protein translation factor
Operon_773	lilo_1251	LILO_RS06520	AguA protein
	lilo_1252	LILO_RS06525	putative polysaccharide deacetylase
	lilo_1253	LILO_RS06530	endoglucanase Y
	lilo_1254	LILO_RS06535	hypothetical protein
	lilo_1255	LILO_RS06540	hypothetical protein
**Regulon_305**			
Operon_362	lilo_0556	LILO_RS03015	GTNG_0265 lantibiotic antimicrobial precursor peptinisin Ade
	lilo_0557	LILO_RS03020	could encode enzyme catalyzing modification reactions
	lilo_0558	LILO_RS03025	nisin transport protein
	lilo_0559	LILO_RS03030	could encode enxyme catalyzing modification reactions
Operon_364	lilo_0561	LILO_RS03035	encodes a protein involved in immunity against nisin
	lilo_0562	LILO_RS03040	cleave leader peptide/cell wall-associated serine proteasee proteinase
Operon_745	lilo_1196	LILO_RS06220	D-alanine transfer protein DltD
	lilo_1197	LILO_RS06225	D-alanyl carrier protein
	lilo_1198	LILO_RS06230	peptidoglycan biosynthesis protein
	lilo_1199	LILO_RS06235	D-alanine activating enzyme
	lilo_1200	lilo_1200	D-Ala-teichoic acid biosynthesis protein
Operon_608	lilo_0970	LILO_RS05065	orotate phosphoribosyltransferase
	lilo_0971	LILO_RS05070	dihydroorotase
**Regulon_306**			

Operon_1254	lilo_2020	LILO_RS10470	arginine/ornitine antiporter
	lilo_2021	LILO_RS10475	hypothetical protein
	lilo_2022	LILO_RS10480	carbamate kinase
	lilo_2023	LILO_RS10485	carbamate kinase
	lilo_2024	LILO_RS10490	arginine/ornithine antiporter
Operon_362	lilo_0556	LILO_RS03015	GTNG_0265 lantibiotic antimicrobial precursor peptinisin Ade
	lilo_0557	LILO_RS03020	could encode enzyme catalyzing modification reactions
	lilo_0558	LILO_RS03025	nisin transport protein
	lilo_0559	LILO_RS03030	could encode enxyme catalyzing modification reactions
Operon_497	lilo_0792	LILO_RS04195	glycerate kinase
	lilo_0793	LILO_RS04200	geranyltranstransferase/ dimethylallyltransferase
	lilo_0794	LILO_RS04205	rRNA methylase
	lilo_0795	LILO_RS04210	transcriptional repressor, arginine deiminase pathway
Operon_366	lilo_0565	LILO_RS03055	ABC transporter ATPase component
	lilo_0566	LILO_RS03060	major facilitator superfamily permease
	lilo_0567	LILO_RS03065	two component system histidine kinase
Operon_32	lilo_0050	LILO_RS00295	hypothetical protein
	lilo_0051	LILO_RS00300	alpha/beta hydrolase
	lilo_0052	LILO_RS00305	glyoxalase family protein
	lilo_0053	LILO_RS00310	flavin reductase family protein
**Regulon_307**			
Operon_29	lilo_0047	LILO_RS00280	O-acetylhomoserine (thiol)-lyase
Operon_686	lilo_1112	LILO_RS05800	dihydroxy-acid dehydratase
	lilo_1113	LILO_RS05805	acetolactate synthase large subunit
	lilo_1114	LILO_RS05810	acetolactate synthase small subunit
	lilo_1115	LILO_RS05815	ketol-acid reductoisomerase
	lilo_1116	LILO_RS05820	threonine deaminase
Operon_1201	lilo_1945	LILO_RS10085	hypothetical protein
Operon_362	lilo_0556	LILO_RS03015	GTNG_0265 lantibiotic antimicrobial precursor peptinisin Ade
	lilo_0557	LILO_RS03020	could encode enzyme catalyzing modification reactions
	lilo_0558	LILO_RS03025	nisin transport protein
	lilo_0559	LILO_RS03030	could encode enxyme catalyzing modification reactions
**Regulon_308**			
Operon_1259	lilo_2027	LILO_RS10510	arginyl-tRNA synthetase
Operon_1093	lilo_1779	LILO_RS09200	glutamate ABC transporter ATP-binding protein
	lilo_1780	LILO_RS09205	glutamate ABC transporter permease protein
Operon_457	lilo_0736	LILO_RS03915	N-acetyl-gamma-glutamyl-phosphate reductase
	lilo_0737	LILO_RS03920	glutamate N-acetyltransferase / amino-acid N-acetyltransferase
	lilo_0738	LILO_RS03925	acetylornithine aminotransferase
	lilo_0739	LILO_RS03930	acetylglutamate kinase

	lilo_0740	LILO_RS03935	ornithine carbamoyltransferase
Operon_53	lilo_0087	LILO_RS00495	argininosuccinate synthase
	lilo_0088	LILO_RS00500	argininosuccinate lyase
Operon_1254	lilo_2020	LILO_RS10470	arginine/ornitine antiporter
	lilo_2021	LILO_RS10475	hypothetical protein
	lilo_2022	LILO_RS10480	carbamate kinase
	lilo_2023	LILO_RS10485	carbamate kinase
	lilo_2024	LILO_RS10490	arginine/ornithine antiporter
**Regulon_309**			
Operon_133	lilo_0213	LILO_RS01150	fructose-1,6-bisphosphatase
Operon_1006	lilo_1629	LILO_RS08435	proline dipeptidase
Operon_X	lilo_2025	LILO_RS10495	ornithine carbamoyltransferase
	lilo_2026	LILO_RS10500	arginine deiminase
Operon_807	lilo_1306	LILO_RS06795	pyruvate kinase
	lilo_1307	LILO_RS06800	6-phosphofructokinase
Operon_964	lilo_1549	LILO_RS08000	transketolase
Operon_1043	lilo_1682	LILO_RS08715	mannose-specific PTS system component IIAB
	lilo_1683	LILO_RS08720	mannose-specific PTS system component IIC
	lilo_1684	LILO_RS08725	mannose-specific PTS system component IID
	lilo_1685	LILO_RS08730	hypothetical protein
Operon_1241	lilo_2001	LILO_RS10375	acetate kinase
Operon_1242	lilo_2002	LILO_RS10380	acetate kinase
Operon_1220	lilo_1974	LILO_RS10235	transcription regulator
Operon_842	lilo_1361	LILO_RS07050	pantothenate kinase
Operon_187	lilo_0303	LILO_RS01620	phosphoglycerate mutase
Operon_1014	lilo_1638	LILO_RS08490	uridine kinase
Operon_1023	lilo_1650	LILO_RS08555	phospho-beta-glucosidase
Operon_1176	lilo_1913	LILO_RS09870	fructose-bisphosphate aldolase
Operon_1005	lilo_1628	LILO_RS08430	catabolite control protein A
Operon_1030	lilo_1662	LILO_RS08615	maltose ABC transporter substrate binding protein
	lilo_1663	LILO_RS08620	maltose transport system permease protein MalF
	lilo_1664	LILO_RS08625	maltose ABC transporter permease protein
Operon_1320	lilo_2128	LILO_RS11055	3-carboxymuconate cyclase
Operon_1102	lilo_1790	LILO_RS09260	alcohol dehydrogenase
Operon_998	lilo_1615	LILO_RS08360	ribose ABC transporter substrate binding protein
	lilo_1616	LILO_RS08365	ribose ABC transporter permease protein
	lilo_1617	LILO_RS08370	ribose ABC transporter ATP binding protein
	lilo_1618	LILO_RS08375	ribose ABC transporter permease protein
	lilo_1619	LILO_RS08380	ribokinase
	lilo_1620	LILO_RS08385	ribose operon repressor
Operon_347	lilo_0532	LILO_RS02890	pyruvate-formate lyase
Operon_1326	lilo_2140	LILO_RS11115	glucose-6-phosphate isomerase
Operon_394	lilo_0629	LILO_RS03395	4-alpha-glucanotransferase

	lilo_0630	LILO_RS03400	glucose-1-phosphate adenylyltransferase
	lilo_0631	LILO_RS03405	glucose-1-phosphate adenylyltransferase
	lilo_0632	LILO_RS03410	glycogen synthase
	lilo_0633	LILO_RS03415	glycogen phosphorylase
	lilo_0634	LILO_RS03420	amylopullulanase/ glucan 1,4-alpha-maltohydrolase
Operon_853	lilo_1377	LILO_RS07130	beta-glucosidase
	lilo_1378	LILO_RS07135	beta-glucoside-specific PTS system IIABC component
	lilo_1379	LILO_RS07140	beta-glucoside operon antiterminator
Operon_1080	lilo_1748	LILO_RS09050	ATP synthase delta subunit
	lilo_1749	LILO_RS09055	ATP synthase subunit b
	lilo_1750	LILO_RS09060	ATP synthase subunit a
	lilo_1751	LILO_RS09065	F0F1-type ATP synthase C subunit
Operon_568	lilo_0906	LILO_RS04760	lactose transport regulator
	lilo_0907	LILO_RS04765	putative 1-phosphofructokinase
	lilo_0908	LILO_RS04770	fructose-specific PTS system enzyme IIBC component
Operon_533	lilo_0847	LILO_RS04465	2,3-butanediol dehydrogenase
	lilo_0848	LILO_RS04470	acetoin reductase
Operon_870	lilo_1412	LILO_RS07315	sugar ABC transporter substrate-binding protein
	lilo_1413	LILO_RS07320	sugar ABC transporter permease protein
	lilo_1414	LILO_RS07325	sugar ABC transporter substrate-binding protein
**Regulon_310**			
Operon_29	lilo_0047	LILO_RS00280	O-acetylhomoserine (thiol)-lyase
Operon_297	lilo_0458	LILO_RS02510	hypothetical membrane protein
	lilo_0459	LILO_RS02515	phosphopantothenoylcysteine synthase/decarboxylase
	lilo_0460	LILO_RS02520	phosphopantothenate--cysteine ligase
Operon_555	lilo_0885	LILO_RS04655	amino acid ABC transporter substrate binding protein
Operon_180	lilo_0284	LILO_RS01525	outer membrane lipoprotein precursor
	lilo_0285	LILO_RS01530	outer membrane lipoprotein precursor
	lilo_0286	LILO_RS01535	outer membrane lipoprotein precursor
	lilo_0287	LILO_RS01540	outer membrane lipoprotein precursor
	lilo_0288	LILO_RS01545	amino acid ABC transporter ATP binding protein
	lilo_0289	LILO_RS01550	amino acid ABC trasporter permease protein
Operon_1271	lilo_2040	LILO_RS10575	amino acid ABC transporter substrate binding protein
Operon_443	lilo_0718	LILO_RS03835	cystathionine beta-lyase
Operon_276	lilo_0429	LILO_RS02270	cysteine synthase
Operon_742	lilo_1192	LILO_RS06200	5,10-methylenetetrahydrofolate reductase
	lilo_1193	LILO_RS06205	5-methionine synthase
Operon_1184	lilo_1923	LILO_RS09970	cystathionine gamma-synthase
	lilo_1924	LILO_RS09975	homoserine O-succinyltransferase
Operon_735	lilo_1185	LILO_RS06165	trans-acting positive regulator
**Regulon_311**			
Operon_192	lilo_0310	LILO_RS01655	oligopeptide ABC transporter substrate binding protein

	lilo_0311	LILO_RS01660	oligopeptide ABC transporter substrate binding protein
	lilo_0312	LILO_RS01665	dipeptide transport system permease protein DppB
	lilo_0313	LILO_RS01670	ABC-type dipeptide/oligopeptide/nickel transport system, permease component
	lilo_0314	LILO_RS01675	oligopeptide ABC transporter ATP binding protein
	lilo_0315	LILO_RS01680	oligopeptide ABC transporter ATP binding protein
Operon_757	lilo_1230	LILO_RS06420	glutamate synthase (NADPH) small chain
	lilo_1231	LILO_RS06425	glutamate synthase (NADPH) large chain
Operon_939	lilo_1515	LILO_RS07825	nitrogen regulatory protein P-II
	lilo_1516	LILO_RS07830	ammonium transporter
Operon_686	lilo_1112	LILO_RS05800	dihydroxy-acid dehydratase
	lilo_1113	LILO_RS05805	acetolactate synthase large subunit
	lilo_1114	LILO_RS05810	acetolactate synthase small subunit
	lilo_1115	LILO_RS05815	ketol-acid reductoisomerase
	lilo_1116	LILO_RS05820	threonine deaminase
Operon_192	lilo_0310	LILO_RS01655	oligopeptide ABC transporter substrate binding protein
	lilo_0311	LILO_RS01660	oligopeptide ABC transporter substrate binding protein
	lilo_0312	LILO_RS01665	dipeptide transport system permease protein DppB
	lilo_0313	LILO_RS01670	ABC-type dipeptide/oligopeptide/nickel transport system, permease component
	lilo_0314	LILO_RS01675	oligopeptide ABC transporter ATP binding protein
	lilo_0315	LILO_RS01680	oligopeptide ABC transporter ATP binding protein
Operon_682	lilo_1094	LILO_RS05710	histidinol-phosphate aminotransferase
	lilo_1095	LILO_RS05715	ATP phosphoribosyltransferase regulatory subunit
	lilo_1096	LILO_RS05720	ATP phosphoribosyltransferase
	lilo_1097	LILO_RS05725	histidinol dehydrogenase
	lilo_1098	LILO_RS05730	SAM-dependent methyltransferase
	lilo_1099	LILO_RS05735	imidazoleglycerol-phosphate dehydratase
	lilo_1100	LILO_RS05740	kanamycin kinase
	lilo_1101	LILO_RS05745	amidotransferase
	lilo_1102	LILO_RS05750	phosphoribosylformimino-5-aminoimidazole carboxamideribotide isomerase
	lilo_1103	LILO_RS05755	imidazoleglycerol-phosphate synthase cyclase
	lilo_1104	LILO_RS05760	phosphoribosyl-AMP cyclohydrolase / phosphoribosyl-ATP pyrophosphohydrolase
	lilo_1105	LILO_RS05765	histidinol phosphatase
Operon_961	lilo_1544	LILO_RS07975	dihydrodipicolinate synthase
	lilo_1545	LILO_RS07980	hypothetical protein
	lilo_1546	LILO_RS07985	aspartate-semialdehyde dehydrogenase
Operon_1159	lilo_1882	LILO_RS09710	PepC protein
Operon_661	lilo_1061	LILO_RS05525	homoserine dehydrogenase
	lilo_1062	LILO_RS05530	homoserine kinase
Operon_1184	lilo_1923	LILO_RS09970	cystathionine gamma-synthase
	lilo_1924	LILO_RS09975	homoserine O-succinyltransferase

Operon_1271	lilo_2040	LILO_RS10575	amino acid ABC transporter substrate binding protein
**Regulon_312**			
Operon_1228	lilo_1983	LILO_RS10285	putative copper homeostasis protein
Operon_494	lilo_0787	LILO_RS04170	copper-potassium transporting ATPase B
	lilo_0788	LILO_RS04175	hypothetical protein
Operon_32	lilo_0050	LILO_RS00295	hypothetical protein
	lilo_0051	LILO_RS00300	alpha/beta hydrolase
	lilo_0052	LILO_RS00305	glyoxalase family protein
	lilo_0053	LILO_RS00310	flavin reductase family protein
Operon_475	lilo_0763	LILO_RS04050	copper transport repressor
	lilo_0764	LILO_RS04055	copper chaperone CopZ
	lilo_0765	LILO_RS04060	copper-translocating P-type ATPase
Operon_216	lilo_0344	LILO_RS01825	acyl carrier protein phosphodiesterase
**Regulon_313**			
Operon_438	lilo_0704	LILO_RS03765	malonyl CoA-acyl carrier protein transacylase
	lilo_0705	LILO_RS03770	3-oxoacyl-[acyl-carrier protein] reductase
	lilo_0706	LILO_RS03775	3-oxoacyl-[acyl-carrier-protein] synthase II
	lilo_0707	LILO_RS03780	acetyl-CoA carboxylase biotin carboxyl carrier protein
Operon_293	lilo_0454	LILO_RS02485	hydroxymyristoyl-acyl carrier protein dehydratase
Operon_294	lilo_0455	LILO_RS02490	enoyl-[acyl-carrier protein] reductase (NADH)
Operon_683	lilo_1106	LILO_RS05770	hypothetical protein
**Regulon_316**			
Operon_1366	lilo_2192	LILO_RS11405	DnaJ protein
Operon_566	lilo_0901	LILO_RS04735	heat-inducible transcription repressor HrcA
	lilo_0902	LILO_RS04740	stress responce protein GrpE
	lilo_0903	LILO_RS04745	DnaK protein
Operon_227	lilo_0356	LILO_RS01885	10 KD chaperonin
Operon_1204	lilo_1948	LILO_RS10100	putative heat shock protein
	lilo_1949	LILO_RS10105	hypothetical protein
**Regulon_320**			
Operon_928	lilo_1497	LILO_RS07740	glucosamine-6-P isomerase
Operon_591	lilo_0947	LILO_RS04965	glucosamine--fructose-6-phosphate aminotransferase
Operon_808	lilo_1309	LILO_RS06810	N-acetylglucosamine-6-phosphate deacetylase
Operon_924	lilo_1493	LILO_RS07720	30S ribosomal protein S16
**Regulon_323**			
Operon_553	lilo_0883	LILO_RS04645	formyltetrahydrofolate synthetase
Operon_1196	lilo_1940	LILO_RS10060	adenylosuccinate synthase
Operon_495	lilo_0789	LILO_RS04180	methylenetetrahydrofolate dehydrogenase (NADP+) / methenyltetrahydrofolate cyclohydrolase
Operon_314	lilo_0487	LILO_RS02655	serine hydroxymethyltransferase
Operon_884	lilo_1439	LILO_RS07455	phosphoribosylaminoimidazolecarboxamide formyltransferase / IMP cyclohydrolase
Operon_840	lilo_1355	LILO_RS07020	basic membrane protein A
Operon_889	lilo_1444	LILO_RS07480	phosphoribosylglycinamide formyltransferase

	lilo_1445	LILO_RS07485	phosphoribosyl-aminoimidazole synthetase
Operon_900	lilo_1459	LILO_RS07550	phosphoribosylformylglycinamidine synthase II
	lilo_1460	LILO_RS07555	phosphoribosylformylglycinamidine synthetase I
	lilo_1461	LILO_RS07560	hypothetical protein
	lilo_1462	LILO_RS07565	phosphoribosylaminoimidazole-succinocarboxamide synthetase
Operon_878	lilo_1431	LILO_RS07410	phosphoribosylaminoimidazole carboxylase ATPase subunit
	lilo_1432	LILO_RS07415	phosphoribosylaminoimidazole carboxylase catalytic subunit
	lilo_1433	LILO_RS07425	phosphoribosylamine-glycine ligase
Operon_M	lilo_1795	LILO_RS09285	inorganic pyrophosphatase
Operon_653	lilo_1046	LILO_RS05450	GMP reductase
Operon_899	lilo_1458	LILO_RS07545	phosphoribosylpyrophosphate amidotransferase
Operon_1061	lilo_1712	LILO_RS08870	hypothetical protein
**Regulon_324**			
Operon_601	lilo_0961	LILO_RS05020	redox-sensing transcriptional repressor REX
Operon_126	lilo_2028	LILO_RS10515	arginine catabolic regulator
Operon_1176	lilo_1913	LILO_RS09870	fructose-bisphosphate aldolase
Operon_806	lilo_1305	LILO_RS06790	L-lactate dehydrogenase
Operon_1321	lilo_2129	LILO_RS11060	alcohol dehydrogenase / acetaldehyde dehydrogenase
Operon_343	lilo_0526	LILO_RS02860	enolase
Operon_147	lilo_0231	LILO_RS01245	enolase (phosphopyruvate hydratase)
**Regulon_325**			
Operon_608	lilo_0970	LILO_RS05065	orotate phosphoribosyltransferase
	lilo_0971	LILO_RS05070	dihydroorotase
Operon_947	lilo_1526	LILO_RS07880	carbamoyl-phosphate synthase small chain
	lilo_1527	LILO_RS07885	aspartate carbamoyltransferase catalytic chain
Operon_53	lilo_0087	LILO_RS00495	argininosuccinate synthase
	lilo_0088	LILO_RS00500	argininosuccinate lyase
Operon_292	lilo_0452	LILO_RS02475	glutamate or arginine ABC transporter substrate binding protein
	lilo_0453	LILO_RS02480	acetylornithine deacetylase
Operon_289	lilo_0448	LILO_RS02455	glyceraldehyde 3-phosphate dehydrogenase
Operon_1075	lilo_1740	LILO_RS09010	glutamine ABC transporter permease and substrate binding protein
	lilo_1741	LILO_RS09015	glutamine ABC transporter ATP-binding protein
Operon_1144	lilo_1854	LILO_RS09570	hypothetical protein
	lilo_1855	LILO_RS09575	cysteinyl-tRNA synthetase
	lilo_1856	LILO_RS09580	hypothetical protein
	lilo_1857	LILO_RS09585	serine acetyltransferase
Operon_364	lilo_0561	LILO_RS03035	encodes a protein involved in immunity against nisin
	lilo_0562	LILO_RS03040	cleave leader peptide/cell wall-associated serine proteasee proteinase
Operon_366	lilo_0565	LILO_RS03055	ABC transporter ATPase component
	lilo_0566	LILO_RS03060	major facilitator superfamily permease

	lilo_0567	LILO_RS03065	two component system histidine kinase
Operon_586	lilo_0934	LILO_RS04900	diaminohydroxyphosphoribosylaminopyrimidine deaminase / 5-amino-6-(5-phosphoribosylamino)uracil reductase
	lilo_0935	LILO_RS04905	riboflavin synthase alpha chain
	lilo_0936	LILO_RS04910	GTP cyclohydrolase II / 3,4-dihydroxy-2-butanone 4-phosphate synthase
	lilo_0937	LILO_RS04915	riboflavin synthase beta chain
	lilo_1219	LILO_RS06365	hypothetical protein
**Regulon_326**			
Operon_586	lilo_0934	LILO_RS04900	diaminohydroxyphosphoribosylaminopyrimidine deaminase / 5-amino-6-(5-phosphoribosylamino)uracil reductase
	lilo_0935	LILO_RS04905	riboflavin synthase alpha chain
	lilo_0936	LILO_RS04910	GTP cyclohydrolase II / 3,4-dihydroxy-2-butanone 4-phosphate synthase
	lilo_0937	LILO_RS04915	riboflavin synthase beta chain
	lilo_1219	LILO_RS06365	hypothetical protein
Operon_362	lilo_0556	LILO_RS03015	GTNG_0265 lantibiotic antimicrobial precursor peptinisin Ade
	lilo_0557	LILO_RS03020	could encode enzyme catalyzing modification reactions
	lilo_0558	LILO_RS03025	nisin transport protein
	lilo_0559	LILO_RS03030	could encode enxyme catalyzing modification reactions

Table S5. Detail information on low-score co-regulatory connections of the single-membered regulons of *L. lactis* IO-1.

Operon ID of single-membered regulon	Connected to	CRS*
Operon_10	Operon_786	0.3
Operon_1085	Operon_1084	0.1
	Operon_921	0.2
Operon_1129	Operon_419	0.3
	Operon_639	0.3
	Operon_720	0.1
	Operon_1606	0.1
Operon_1181	Operon_1182	0.2
Operon_1218	Operon_1219	0.3
Operon_1282	Operon_1284	0.1
Operon_135	Operon_126	0.4
	Operon_1014	0.3
	Operon_1024	0.1
Operon_1374	Operon_1375	0.2
Operon_174	Operon_461	0.3
	Operon_465	0.2
Operon_255	Operon_248	0.1
Operon_26	Operon_268	0.4
	Operon_1174	0.2
	Operon_1181	0.1
Operon_265	Operon_159	0.3
Operon_464	Operon_1229	0.3
	Operon_891	0.1
Operon_536	Operon_683	0.4
Operon_645	Operon_755	0.3
	Operon_904	0.3
	Operon_890	0.2
Operon_777	Operon_221	0.1
	Operon_210	0.1
	Operon_698	0.2
Operon_805	Operon_808	0.2
Operon_841	Operon_409	0.1
	Operon_131	0.2
	Operon_1078	0.1
Operon_858	Operon_181	0.4
	Operon_1254	0.3
Operon_885	Operon_891	0.2
Operon_906	Operon_1322	0.3
	Operon_171	0.1
Operon_960	Operon_968	0.3
	Operon_400	0.2

Table S6. Nisin production affected by riboflavin production studied in two conditions

a. Different [riboflavin] in culture medium						
**[Riboflavin] (mg/L)**	**DCW* (g/L)**	**±SD****	**Nisin activity (IU/ml)**	**±SD**	**Nisin Yield (×10^6 IU/g-DCW)**	**±SD**
**1**	0.67	0.001247	596	4.921608	0.889	0.00753
**0.8**	0.65	0.00411	573	2.867442	0.881	0.007108
**0.6**	0.61	0.00216	538	6.377042	0.882	0.010911
**0.4**	0.6	0.009672	525	5.09902	0.875	0.016468
**0.2**	0.56	0.008165	528	8.164966	0.926	0.020039
**0.1**	0.57	0.01654	507	6.944222	0.889	0.02854
**0.007**	0.54	0.004497	476	7.25718	0.881	0.015313
**0.004**	0.53	0.004714	430	3.681787	0.811	0.010017
**0.001**	0.53	0.000943	415	2.94392	0.783	0.005727
**0**	0.51	0.008498	400	4.546061	0.784	0.01582
**b. Assay using riboflavin overproducer mutants**						
**Strains**	**DCW (g/L)**	**±SD**	**Nisin activity (IU/ml)**	**±SD**	**Nisin Yield (×10^6 IU/g-DCW)**	**±SD**
**IO-1**	0.67	0.001247	596	4.921608	0.889	0.00753
**LR01**	0.68	0.00793	425	5.734884	0.625	0.011147
**LR02**	0.64	0.050135	476	5.249339	0.743	0.058837
**LR03**	0.62	0.00776	422	5.09902	0.68	0.011841
**LR04**	0.65	0.0133	425	4.898979	0.654	0.015356
**LR05**	0.66	0.008731	441	4.189935	0.668	0.010882
**LR06**	0.61	0.014142	418	3.399346	0.685	0.016836

** Dry cell weight.*

*** Standard deviation (for nisin yield which can be derived from nisin activity and DCW, the standard deviation was calculated using error propagation method).*

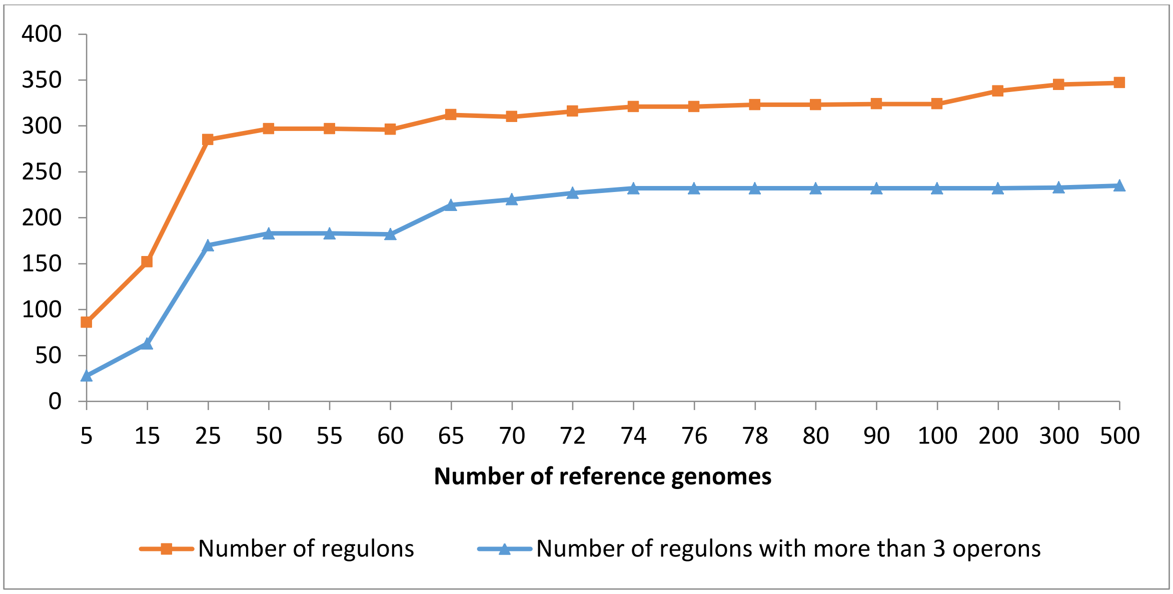

Figure S1. Effect of number of selected reference genomes on the final number of predicted regulons in *L. lactis* IO-1 using phylogenetic footprinting approach. *(A set of 74 genomes were selected due to two main reasons: 1, it was the lowest number of reference genomes resulting in the prediction of maximum number of regulons containing more than 3 operons and also more reference genomes did not meaningfully affect the number of regulons; 2. Higher number of reference genomes increased the false positives in motif prediction due to high divergence in genomes).*
